# Synthesis and Characterization of NUC-7738, an Aryloxy Phosphoramidate of 3′-Deoxyadenosine, as a Potential Anticancer Agent

**DOI:** 10.1021/acs.jmedchem.2c01348

**Published:** 2022-11-23

**Authors:** Michaela Serpi, Valentina Ferrari, Christopher McGuigan, Essam Ghazaly, Chris Pepper

**Affiliations:** † School of Chemistry, Cardiff University Main Building, Park Place, Cardiff CF10 3AT, Wales, U.K.; ‡ School of Pharmacy and Pharmaceutical Sciences, Cardiff University, Cardiff, King Edward VII Avenue, Cardiff CF10 3NB, U.K.; § Centre for Haemato-Oncology, Barts Cancer Institute, Queen Mary University of London, Charterhouse Square, London EC1M 6BQ, U.K.; ∥ Brighton and Sussex Medical School, University of Sussex, Brighton BN1 9PX, U.K.

## Abstract

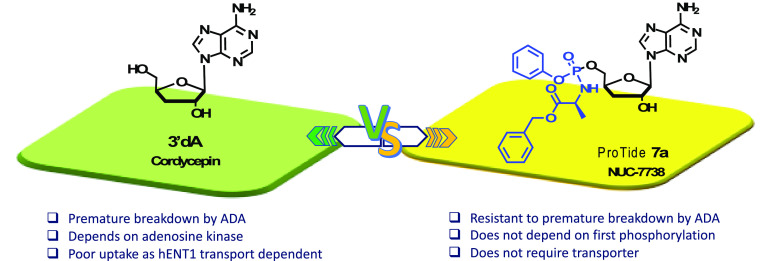

3′-Deoxyadenosine (3′-dA, Cordycepin, **1**) is a nucleoside analogue with anticancer properties, but its clinical development has been hampered due to its deactivation by adenosine deaminase (ADA) and poor cellular uptake due to low expression of the human equilibrative transporter (hENT1). Here, we describe the synthesis and characterization of NUC-7738 (**7a**), a 5′-aryloxy phosphoramidate prodrug of 3′-dA. We show *in vitro* evidence that **7a** is an effective anticancer drug in a panel of solid and hematological cancer cell lines, showing its preferential cytotoxic effects on leukemic stem cells. We found that unlike 3′-dA, the activity of **7a** was independent of hENT1 and kinase activity. Furthermore, it was resistant to ADA metabolic deactivation. Consistent with these findings, **7a** showed increased levels of intracellular 3′-deoxyadenosine triphosphate (3′-dATP), the active metabolite. Mechanistically, levels of intracellular 3′-dATP were strongly associated with *in vitro* potency. NUC-7738 is now in Phase II, dose-escalation study in patients with advanced solid tumors.

## Introduction

**Figure 1:**
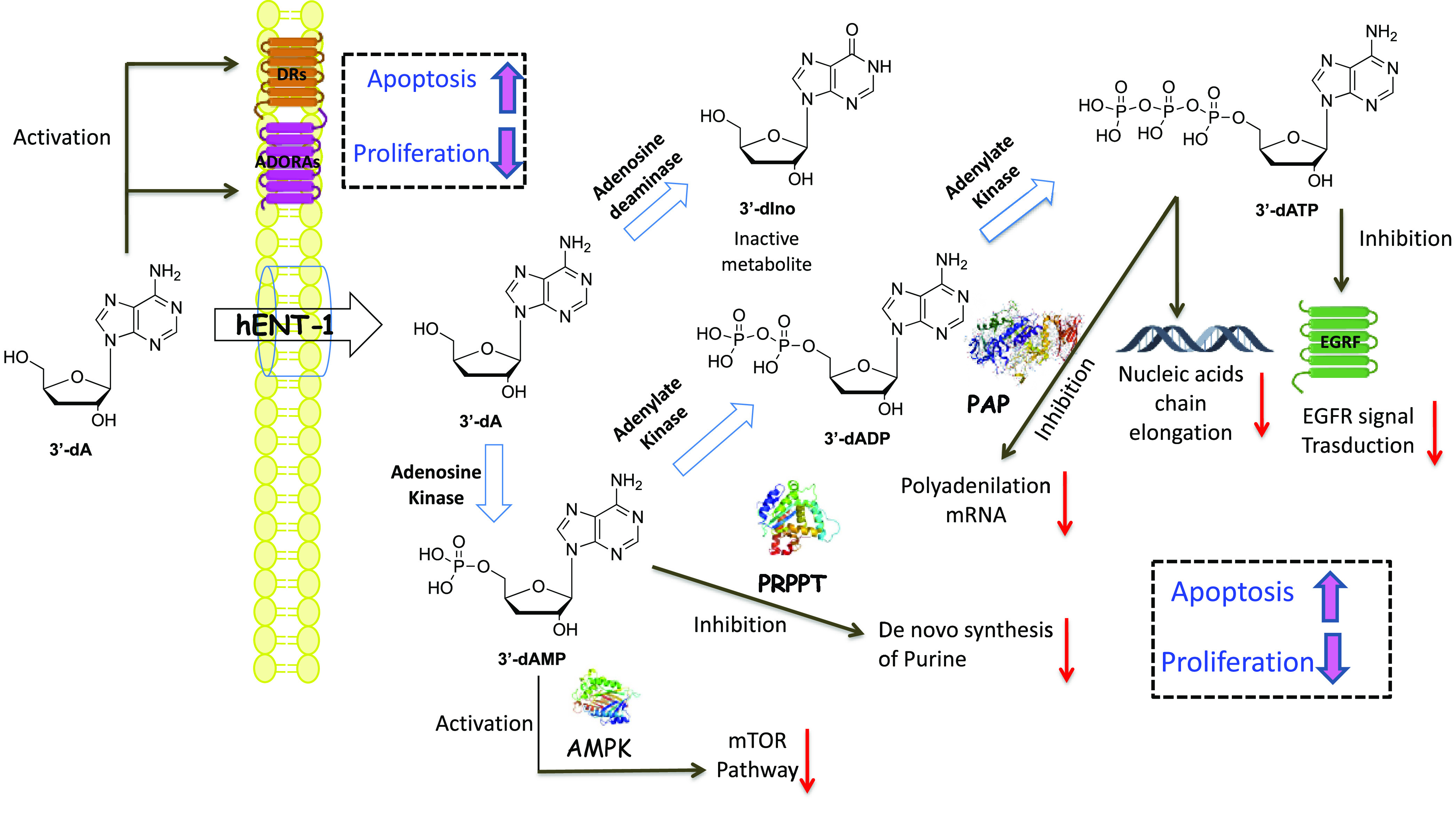
Transport (gray arrow), metabolism (blue arrows), and mode of action (black arrows) of 3′-dA and its metabolites. 3′-dA enters the cell via the human equilibrative nucleoside transporters (hENT1), then it is phosphorylated by adenosine kinase (AK) to 3′-deoxyadenosine monophosphate (3′-dAMP), which in turn is phosphorylated twice to 3′-deoxyadenosine triphosphate (3′-dATP) by the combined actions of adenosine monophosphate kinase (AMPK) and nucleoside diphosphate kinases (NDPK). 3′dA is metabolized to 3′-deoxyinosine (3′-dIno) by adenosine deaminase (ADA). Mode of action: 3′-dATP provokes termination of RNA elongation and inhibits polyadenylate polymerase (PAP). It also interrupts EGFR signal transduction. 3′-dAMP inhibits the activity of phosphoribosyl-pyrophosphate aminotransferase (PRPPT) and activates AMPK. 3′-dA mediates apoptotic signaling via adenosine (ADORAs) and death receptors (DRs) to inhibit lung cancer cell proliferation and induce apoptosis. All of these activities lead to apoptosis and inhibition of cell proliferation.

3′-Deoxyadenosine (3′-dA, cordycepin, **1**) (Figure 1) is the main bioactive component isolated from the fermented broth of *Cordyceps militaris*, which is a medicinal parasitic fungus. This fungus has been used in traditional Chinese medicine for over 300 years as a treatment for inflammatory diseases and cancer.^1−5^ 3′-dA is a nucleoside analogue similar in structure to adenosine but lacking the 3′-hydroxyl group on the ribose moiety, and it has been proposed as an anticancer drug due to its numerous biological and pharmacological actions including inhibition of cell proliferation, induction of apoptosis, antimetastatic effect, and immune system activation.^6^ The mechanism of its antitumor activity is not well understood, but 3′-dA has been shown to regulate several signaling pathways associated with tumor growth and metastasis.^7,8^ 3′-dA is thought to enter cells via the human equilibrative nucleoside transporters (hENT1),^9^ and to be phosphorylated by adenosine kinase (AK) to 3′-deoxyadenosine monophosphate (3′-dAMP). 3′-dAMP is phosphorylated twice to the active metabolite 3′-deoxyadenosine triphosphate (3′-dATP) by the combined actions of adenosine monophosphate kinase (AMPK) and nucleoside diphosphate kinases (NDPK) (Figure 1).^10^ 3′-dA is also subject to metabolism by adenosine deaminase (ADA), which rapidly metabolizes it to the inactive 3′-deoxyinosine (3′-dIno, Figure 1).^11−13^ Due to the structural similarity with adenosine triphosphate (ATP), 3′-dATP can provoke termination of RNA elongation by incorporating into the site where nucleic acids are supposed to incorporate.^10^ 3′-dATP may also compete with ATP for binding to the epidermal growth factor receptor (EGFR) binding site. It has been suggested that 3′-dATP blocks the phosphorylation of EGFR and therefore interferes with the caspase and GSK-3β mediated pathways.^14^ 3′-dATP has also been shown to inhibit polyadenylate polymerase (PAP), an enzyme responsible for the polyadenylation of mRNA.^10^ Furthermore, 3′-dAMP inhibits the activity of phosphoribosyl-pyrophosphate aminotransferase (PRPPT), which impedes *de novo* purine synthesis,^15^ and activates AMPK downregulating mTORC1 function and HIF-1α expression in tumor cells.^16^ 3′-dA has been suggested to mediate apoptotic signaling via adenosine receptors (ADORAs) in glioma,^17^ bladder,^18^ melanoma, and lung carcinoma^19^ cell lines and via death receptors (DRs) in colon and prostate cancer cell lines.^20,21^ Despite all of these activities and the potent anticancer activity observed in *in vitro* studies, 3′-dA has not been successfully developed or approved as a chemotherapeutic agent. 3′-dA efficacy is severely limited by its rapid metabolism into an inactive metabolite by ADA. Downregulation of hENT1 transporter and lack of expression of kinase enzymes, which have been found after drug resistance develops in multiple cancers,^22^ are also responsible for the reduced efficacy of 3′dA due to its poor cellular uptake and activation.^13,23^ In response to some of these limitations, 3′-dA entered clinical trials in combination with the ADA inhibitor, pentostatin. Two phase 1 clinical studies in patients with refractory acute lymphocytic or chronic myelogenous leukemia^24^ and phase I/II studies in patients with refractory TdT-positive leukemia^25^ have been reported; however, it remains unclear if either of these studies completed recruitment or generated results. It is worthy of note that given the critical role that ADA plays in several human metabolic pathways, treatment strategies that require the co-administration of an ADA inhibitor may restrict the clinical development of 3′-dA.

**Figure 2:**
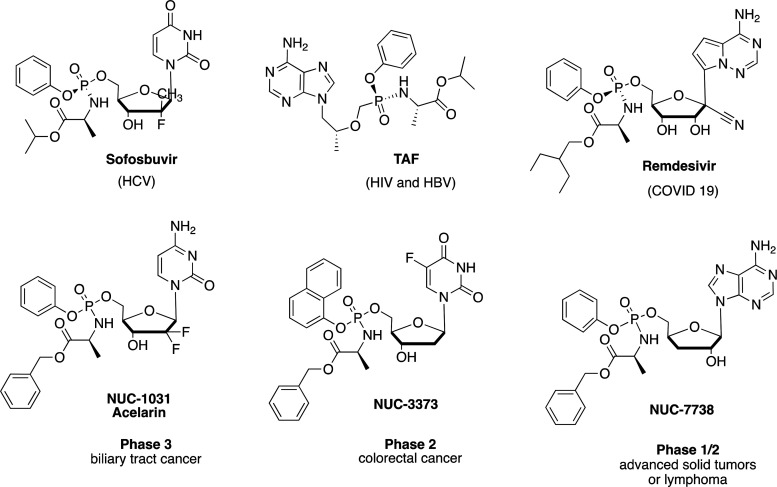
FDA-approved and clinical candidate ProTides.

Exploiting phosphoramidate chemistry, nucleoside analogues have been converted into activated nucleotide analogues with the addition at the 5′ position of a phosphate group protected by specific combinations of aryl and amino acid ester moieties.^26^ These prodrugs are designed to deliver the active metabolite of the parental nucleoside directly into cells, bypassing the resistance mechanisms associated with transportation, activation, and breakdown. The ProTide approach has been particularly successfully applied to antiviral nucleosides^27^ as evidenced by the development and approval of the currently marketed drugs Sofosbuvir^28^ and Tenofovir Alafenamide (TAF),^29^ respectively, approved for the treatment of HCV and HIV, and Remdesivir, authorized for temporary use as a COVID-19 treatment (Figure 2).^30^ In the oncology setting, three clinical candidates Acelarin,^31,32^ NUC-3373,^33^ and NUC-7738^34^ are currently under clinical evaluation as anticancer agents (Figure 2).^35−41^ Here, we report the design, synthesis, and biological evaluation of a family of phosphoramidates of 3′-dA, which were designed to bypass the premature breakdown by ADA of 3′-dA and its hENT-1 and kinase dependence. This study has led to the selection of NUC-7738 as a clinical candidate, now in phase 1/2 clinical evaluation for the treatment of advanced solid tumors or lymphomas.^37,41^

## Results and Discussion

### Chemistry

**Scheme 1:**
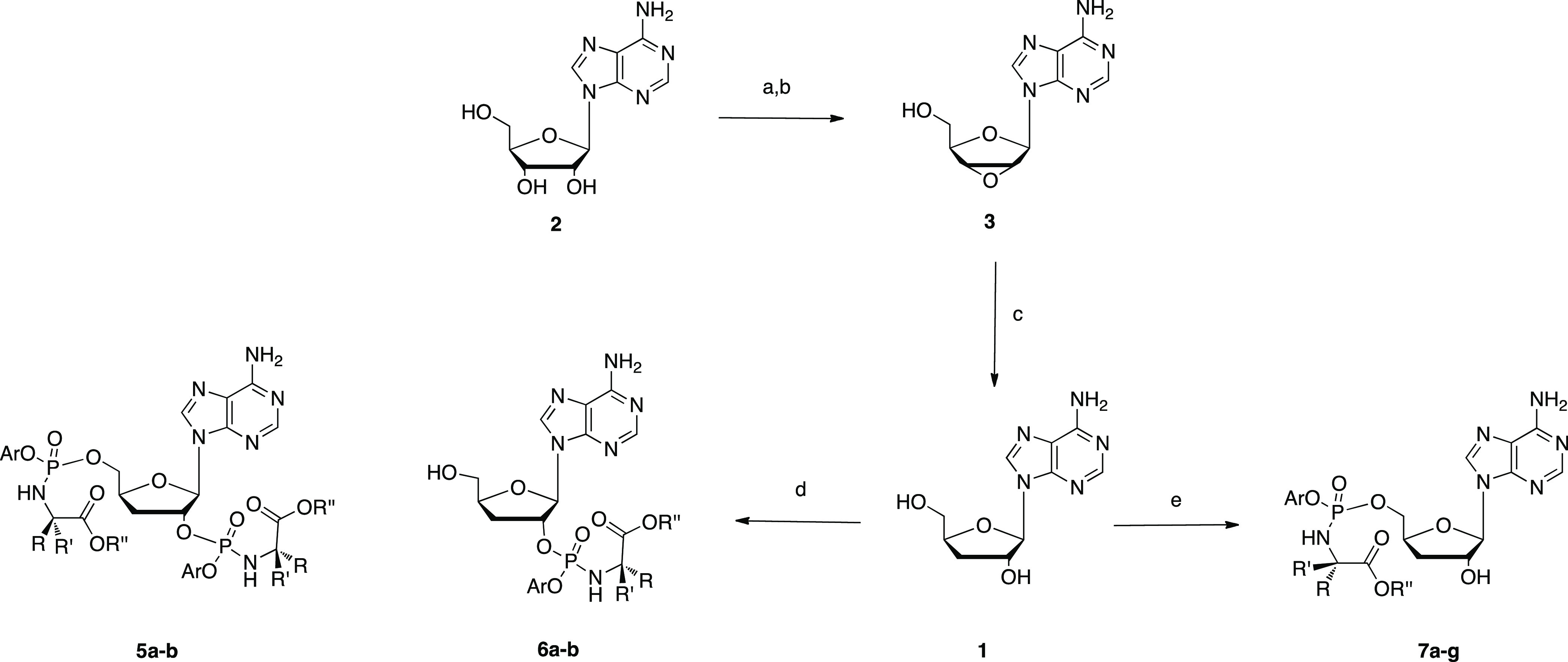
Reagents and Conditions: (a) α-AIBBr (4 equiv), H_2_O/CH_3_CN, rt, 1h; (b) Amberlite (IRN78) (8 mL/mmol), CH_3_OH, rt, 16 h, 98%; (c) LiEt_3_BH DMSO (4 mL/mmol) tetrahydrofuran (THF, 10 mL/mmol), 0 °C to rt, 16 h, 95%; (d) 1 M *t*BuMgCl in THF (1.1 equiv), **4a–b** (3 equiv), anhydrous THF, rt, 16 h, 5–35% (e) NMI (5 equiv), **4a–f** (3 equiv), anhydrous THF, rt, 16 h, 6–28%

In the past, 3′-dA was mainly obtained by extraction from *Cordyceps militaris*. However, due to the low amount of 3′-dA in the fungus (2–3 mg/kg), chemical synthesis has slowly replaced extraction as a means of producing it. Many different strategies for the synthesis of 3′-dA starting either from non-nucleoside^42−45^ or nucleoside starting materials^46−50^ are reported in the literature. After careful consideration, we decided to follow the synthetic route developed by Meier et al. (Scheme 1).^48^ Briefly, adenosine (**2**) was first converted into 9-(2,3-anhydro-β-d-ribofuranosyl)adenine (**3**) with a 98% yield. Then, metal hydride-mediated reductive opening of the epoxide ring in **3** afforded cordycepin (**1**) as the only regioisomer with a 95% yield. To generate phosphoramidates of 3′-dA, we first applied the procedure involving the use of *t*-butyl magnesium chloride (*t*BuMgCl) as a base and a phosphochloridate as a phosphorylating reagent.^51^ However, the reaction of phosphochloridates **4a–b** with **1** in these conditions led to the formation of two products, which, through careful analysis of two-dimensional NMR spectra, were identified as the 2′,5′-*O*-bis phosphoramidates **5a–b** and 2′-*O*-phosphoramidates **6a–b**. On the contrary, when the coupling reaction was performed with *N*-methyl imidazole (NMI),^51^ 5′-phosphoramidates **7a–g** were synthesized as the major products in moderate yields, together with the corresponding 2′,5′-*O*-bis phosphoramidates as side products (not isolated). The choice of promoieties was based on our previous findings (Table 1).^26^

**Table 1:**
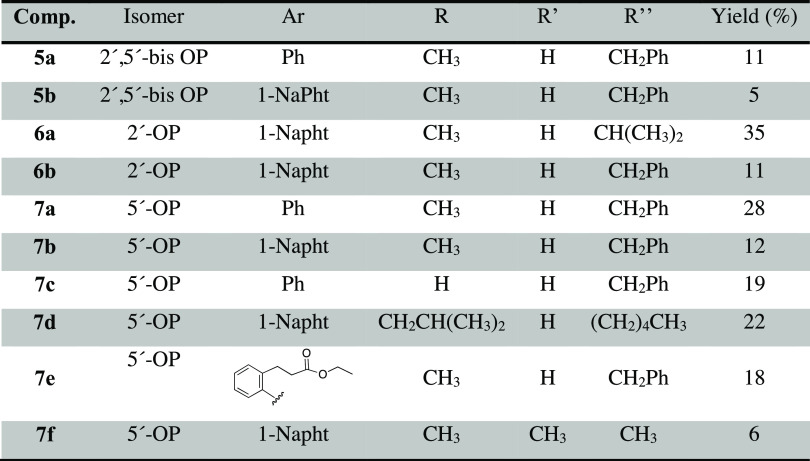
Chemical Composition and Yield of the Synthesized 3′-dA ProTides

**Figure 3:**
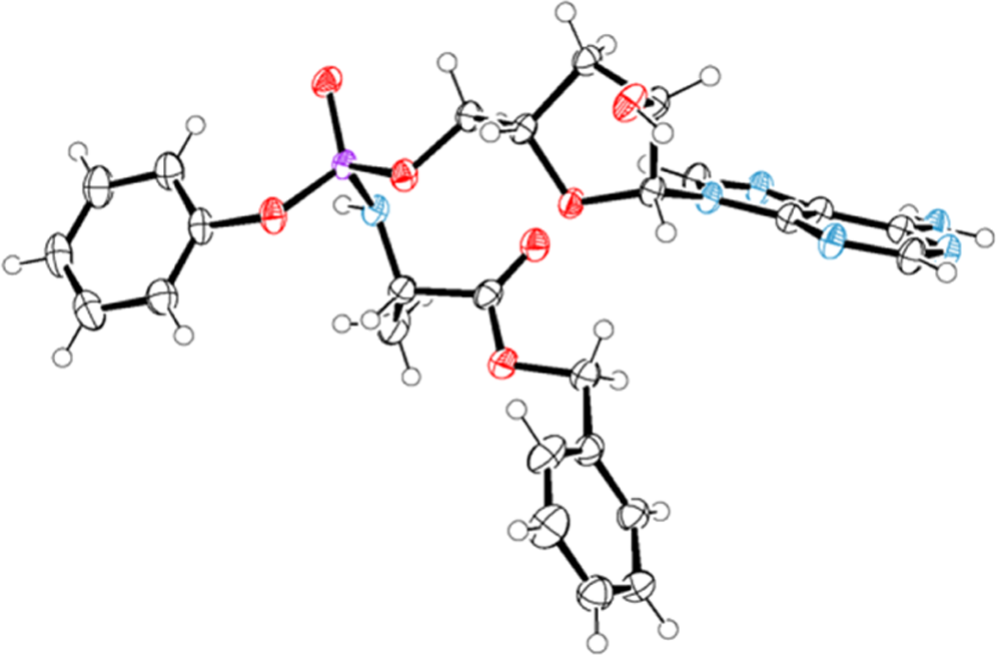
ORTEP view of the **7a-*****S*****p** molecule crystal structure from the ethyl acetate solvate. All nonhydrogen atoms are shown with thermal ellipsoids set at the 50% probability level. Color code: carbon = gray, hydrogen = white, oxygen = red, and phosphorous = purple.

Differences in the stereochemistry of drugs have often led to compounds with different activities, toxicologies, and metabolisms, hence the preference for using single isomers as clinical agents. In the case of ProTides, crucial examples are the marketed drugs sofosbuvir and TAF, whose *S*p isomers were 18-fold and 10-fold more active than the *R*p isomers, respectively.^52,53^ Other examples of ProTides, whose separated diastereoisomers showed different activity profiles,^54^ rates of enzymatic activation^33,55^ or differential transport through intestinal absorption cell models are also reported in the literature.^56^ Although reported, diastereoisomeric separation^57,58^ of ProTides or their preparation^59−61^ as single diastereoisomers are not always straightforward. Our attempts to separate the diastereoisomers of ProTides **7a** (NUC-7738) (selected compound from this study) via direct phase silica gel column chromatography were ineffective due to the very close retention times of both isomers on silica. Attempts to separate 3′-dA phosphoramidate diastereoisomers bearing different silane protecting groups at the 2′ position, as previously reported for ProTides of different nucleosides,^62^ were also unsuccessful. This protection strategy, although ineffective for the separation of the two diastereoisomers, proved to be a more efficient way to prepare **7a**, with an overall yield of 42%.^34^ Separation of the isomers of **7a** was instead achieved via reversed-phase high-performance liquid chromatography (Figure S1) or Biotage Isolera using isocratic elution with 55% methanol in water. Structural elucidation studies were subsequently performed on **7a** slow-eluting isomer, obtained from reversed-phase separation. Single-crystal analysis from crystals obtained from ethyl acetate confirmed the presence of a 1:1 solvated species and the absolute configuration of this isomer was determined to be *S* around the phosphorus chiral center. The asymmetric unit is shown in Figure 3.

**Scheme 2:**
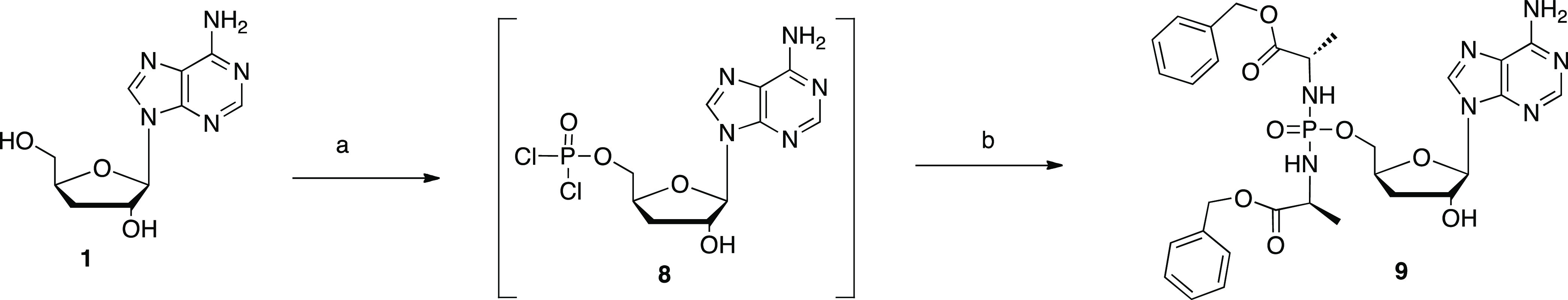
Reagents and Conditions: (a) POCl_3_ (1 equiv), TMP, 0 °C to rt, 4 h. (b) l-AlaOBn·HCl (5 equiv), *N*,*N*-diisopropylethylamine (DIPEA, 10 equiv), CH_2_Cl_2_, 0 °C rt, 16 h, 49%

Although less investigated than the ProTides, the phosphorodiamidates in which two identical amino acid esters are introduced onto the monophosphate moiety through a P–N bond to mask the negative charges have been reported as valid alternatives for the delivery of nucleoside monophosphates into a cell.^63,64^ Due to their symmetric structure, phosphorodiamidates offer the advantage of not having a chiral phosphorus compared to the aryloxy monoamidate analogues. As previously reported for other nucleosides, we used a slightly modified Yoshikawa procedure applied to 3′-dA to obtain bis benzoxy-l-alaninyl phosphorodiamidate **9** from unprotected 3′-dA with a 49% yield (Scheme 2).^65^

### *In Vitro* Cytotoxic Activity

All of the synthesized compounds and the parent nucleosides were tested against a wide panel of cancer cell lines (Tables 2–4 and Table S1). In the majority of cell lines investigated, 3′-dA did not show potent anticancer activity, as evidenced by the high LC_50_ values (the concentration required to kill 50% of the cells in culture) and the inability to reach 100% cell death. The exceptions were the mantle cell lymphoma cell line, Z138 (LC_50_ = 12.15 μM), and the erythroleukemia cell line, HEL92.1.7 (LC_50_ = 68.9 μM). No rationale for these dichotomous results could be found in the literature, but it might be speculated that these cell lines have lower constitutive levels of ADA, which could enhance the activity of 3′-dA.

**Table 2:** Cytotoxic Activity of 3′-dA and Compounds **7a–f, 9**^a^

	CCRF-CEM		HL-60		KG-1		MOLT-4		K562		MV4-11		THP-1	
comp	LC_50_	MI_%_	LC_50_	MI_%_	LC_50_	LC_50_	LC_50_	MI_%_	LC_50_	MI_%_	LC_50_	MI_%_	LC_50_	MI_%_
3′-dA	>198	12	76.84	88	70.82	78	151.92	52	59.31	88	>198	1	>198	–3
**7a**	2.36	100	17.78	97	17.36	92	0.51	98	6.1	92	2.1	99	65.45	74
***Rp*****-7a**	1.86	97	8.7	100	14.0	90	0.57	98	2.60	95	0.98	100	35.0	91
***Sp-*****7a**	2.04	97	11	92	18.0	90	0.43	98	3.0	96	1.03	100	66.0	69
**7b**	1.17	100	6.37	100	11.03	100	0.32	100	4.93	97	1.48	99	46.47	99
**7c**	14.93	94	45.23	82	136.56	65	3.55	93	15.42	92	-	-	-	-
**7d**	4.72	100	8.9	100	12.02	99	1.46	100	11.7	99	8.78	106	68.91	99
**7e**	40.09	90	78.51	74	102.14	69	12.62	97	174.71	61	3.27	100	36.66	100
**7f**	4.92	99	24.08	98	>198	29	1.4	100	10.69	91	8.71	101	>198	43
**9**	7.03	100	16.53	100	34.13	98	3.52	100	24.34	97	-	-	-	-
**PTX**	0.003	95	0.004	96	0.07	89	0.002	97	0.008	94	0.01	99	0.03	72

a Cytotoxicity data reported as μM LC_50_ values (concentration of drug causing 50% cell death) and MI% values (maximum inhibitory effect of the drug at the range of concentrations considered). PTX: paclitaxel (control); (-) not tested.

As is evidenced by the results, the 3′-dA prodrugs conferred an advantage over the parental nucleoside in terms of cytotoxicity in almost all cell lines. Among all of the prodrugs, the 5′-phosphoramidates (**7a–f**) were the most active in hematological tumor cell lines, reported in Tables 2 and 3, and their results against solid tumor cell lines are shown in Table 4 (see Table S1 in the Supporting Information for results of the 2′,5′-*O*-bis phosphoramidates **5a,b** and the 2′-phosphoramidates **6a,b**). Among the 5′ derivatives, **7a** and **7b** emerged as the most active in most of the cell lines tested. The l-leucine pentyl derivative **7d** also showed potent anticancer activity but was always 1- to 3-fold less active compared to **7a** and **7b**. Replacing the amino acid portion with glycine, as in **7c** or dimethylglycine **7f**, led to a decrease in anticancer activity, especially in the case of compound **7f**, which also contained a methyl ester instead of a benzyl ester. The introduction of an ethyl propanoic ester side chain in the *ortho* position of the phenyl ring generated compound **7e**, which had similar or higher LC_50_ values compared to **7a** and **7b**. This side chain was strategically introduced, as upon activation, compound **7e** releases hydroxyphenyl propanoic acid, which is a known nontoxic metabolite of dihydrocoumarin, a common flavoring agent widely used in food and cosmetics.^66^

**Table 3:** Cytotoxic Activity of 3′-dA and Compounds **7a,b, d–f**^a^

	HEL92.1.7		NCI-H929		RPMI-8226		Jurkat		Z138		RL		HS445	
comp	LC_50_	MI_%_	LC_50_	MI_%_	LC_50_	MI_%_	LC_50_	MI_%_	LC_50_	MI_%_	LC_50_	MI_%_	LC_50_	MI_%_
**3′-dA**	68.9	88	>198	24	>198	1	>198	20	12.15	95	>198	17	>198	2
**7a**	8.07	100	6.07	100	14.72	96	1.44	100	26.97	95	3	93	30.53	98
***Rp*****-7a**	11.0	98	19.0	97	25.0	90.8	2.82	97	-	-	4.0	96.8	42.0	99
***Sp-*****7a**	11.0	100	16.0	94	19.0	86.8	2.29	90	-	-	2.64	92.6	67.0	75
**7b**	2.94	98	3.38	99	9.48	102	0.9	100	6.32	100	1.68	96	10.18	96
**7d**	4.23	99	7.21	104	24.06	103	7.15	100	5.65	100	11.61	100	39.53	102
**7e**	14.49	99	7.57	100	31.05	106	4.75	100	68.18	76	7.26	90	42.73	92
**7f**	15.42	101	16.11	98	46.7	89	7.01	95	43.84	93	13.54	88	54.98	85
**PTX**	0.02	83	0.003	82	0.003	91	0.005	97	0.002	99	0.003	83	0.01	74

a Cytotoxicity data reported as μM LC_50_ values (concentration of drug causing 50% cell death) and MI% values (maximum inhibitory effect of the drug at the range of concentrations considered). PTX: paclitaxel (control); (-) not tested.

**Table 4:** Cytotoxic Activity of 3′-dA and Compounds **7a–f, 9**^a^

	HepG2		MCF-7		BxPC-3		HT29		MIA PaCa-2		SW620	
comp	LC_50_	MI_%_	LC_50_	MI_%_	LC_50_	MI_%_	lC_50_	MI_%_	LC_50_	MI_%_	LC_50_	MI_%_
**3′-dA**	142.47	66	34.23	78	>198	22	>198	44	>198	35	>198	10
**7a**	18.73	76	3.07	94	23.59	81	13.42	93	6.85	96	24.94	85
***Sp-*7a**	57.0	73	5.87	94	99.0	73	25.0	95	18.0	94	35.0	87
***Rp-*7a**	32.0	78	2.94	97	74.0	71	16.0	97	14.0	96	29.0	82
**7b**	11.53	95	1.39	99	13.36	90	7.52	98	3.73	98	11.41	93
**7c**	142.35	59	15.46	87	-	-	-	-	-	-	-	-
**7d**	9.53	99	2.48	100	65.46	99	16.06	99	14.69	106	35.48	100
**7e**	96.11	59	15.78	78	60.92	78	25.24	89	12.09	101	43.4	90
**7f**	70.22	84	9.10	99	76.18	71	37.94	76	17.55	91	56.48	74
**9**	156.87	55	12.97	97	-	-	-	-	-	-	-	-
**PTX**	#intersect	54	0.003	79	>0.5	42	0.004	75	0.002	87	0.02	96

a Cytotoxicity data reported as μM LC_50_ values (concentration of drug causing 50% cell death) and MI% values (maximum inhibitory effect of the drug at the range of concentrations considered). PTX: paclitaxel (control); (-) not tested.

ProTides **7a** and **7b** were approximately 200-fold more active than 3′-dA and in the Jurkat cell line (acute T-cell leukemia) with LC_50_ values of 1.44 and 0.9 μM, respectively. Their cytotoxic activities in solid tumor cell lines were lower compared to hematological tumors, but again, ProTides **7a** and **7b** were the most active against this panel of cell lines. The *in vitro* cytotoxic activities of the individual diastereomers **7a**-*S*_p_ and **7a**-*R*_p_ were similar to their diastereomeric mixture. Phosphorodiamidate **9**, although more active than the parental nucleoside, was less potent than the aryloxyphosphoramidates **7a** and **7b** in all of the cell lines tested.

**Figure 4:**
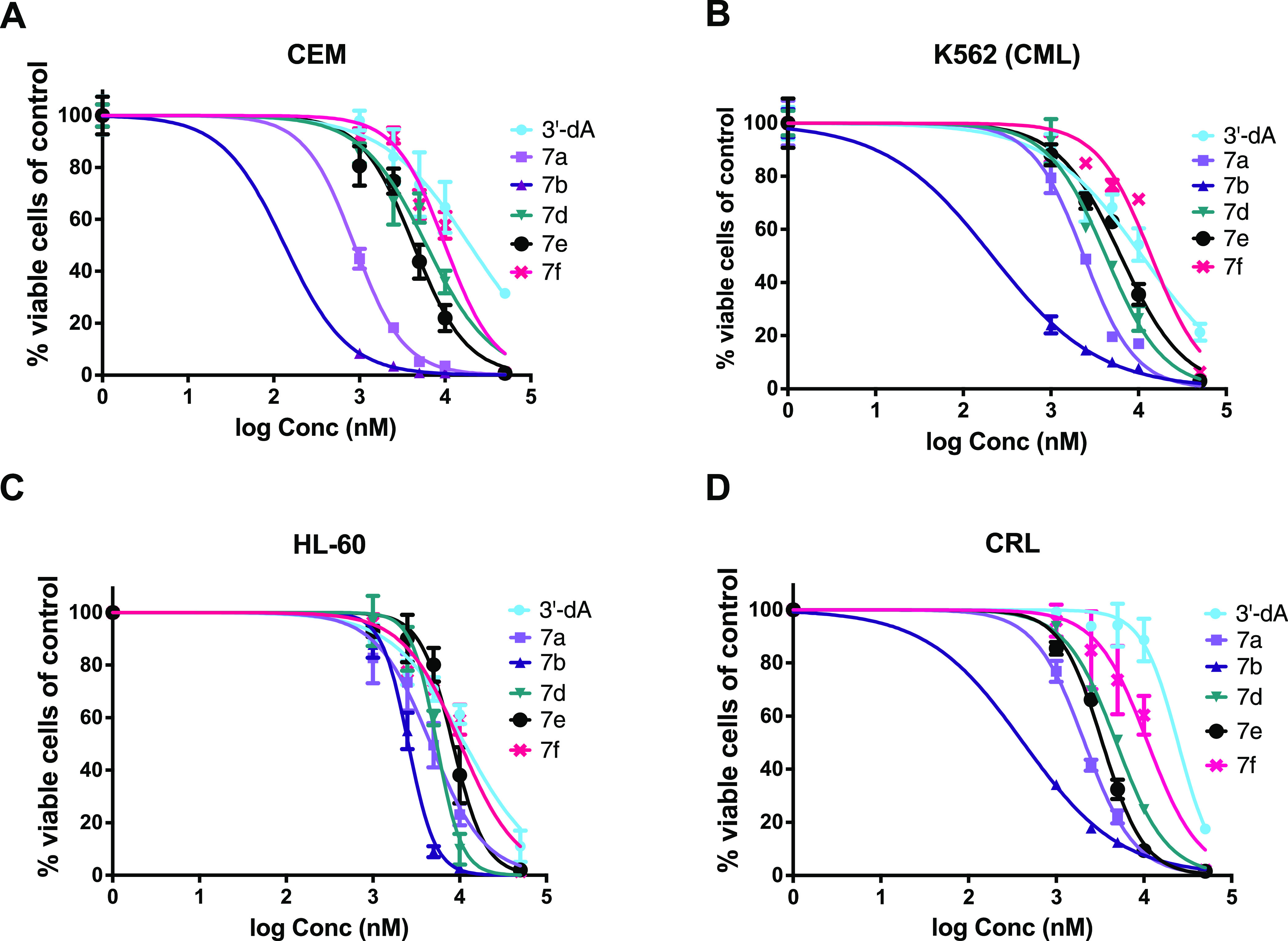
Comparative cytotoxicity of 3′-dA and ProTides **7a,b, d–f** in (A) CEM (B), K562 (C), HL-60, and (D) CRL cell lines in the absence of hENT1, AK, and ADA inhibitors.

The *in vitro* cytotoxic activity of the synthesized ProTides and 3′-dA were then evaluated in five hematological cancer cell lines (CEM, K562, HL60, CRL, and KG1a) (Figure 4) and subsequently, the cytotoxicity observed in 4/5 cell lines (CEM, K562, HL60, CRL) was correlated with the intracellular level of 3′-dATP achieved (measured by liquid chromatography–mass spectrometry). ProTide **7c** and phosphorodiamidate **9** were excluded from these studies due to their lack of potency in the cytotoxic screening.

As depicted in Figure 4A–D, compounds **7a** and **7b** were the most active ProTides with LC_50_ values 3- to 150-fold lower than 3′-dA in all four cell lines (Table 2). Compounds **7d** and **7e** were also more cytotoxic than 3′-dA but were less potent than **7a** and **7b**. Compound **7f** showed only a marginal (1–2-fold) improvement in activity over 3′-dA (Table S2).

**Figure 5:**
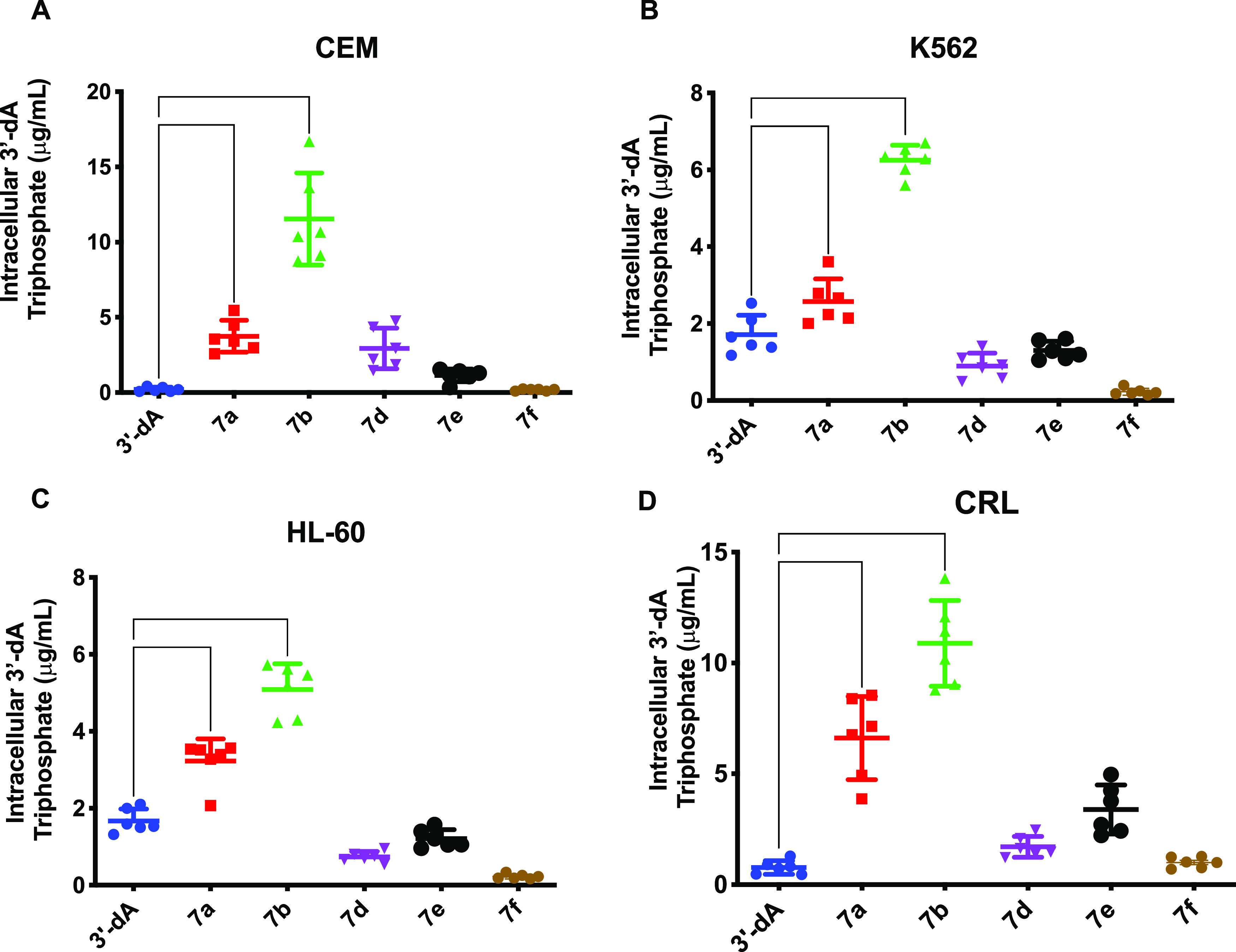
Determination of the intracellular accumulation of 3′-dATP after treatment with 3′-dA and prodrugs **7a,b**, **d–f** in the absence of hENT1, AK, and ADA inhibitors. Differences in intracellular triphosphate were assessed in (A) CEM, (B) K562, (C) HL-60, and (D) CRL cell lines, using one-way analysis of variance (ANOVA) with Tukey’s correction for multiple comparisons. **** *P* < 0.0001, *** *P* < 0.001, ** *P* < 0.01, * *P* < 0.05.

The intracellular accumulation of 3′-dATP was associated with the cytotoxicity of each compound. ProTides **7a** and **7b** generated 3′-dATP intracellular concentrations 3- to 56-fold higher than 3′-dA, whereas **7d** and **7e** showed only 2- to 15-fold increase in 3′-dATP compared to 3′-dA. Treatment with compound **7f** resulted in the lowest 3′-dATP intracellular levels across the four cell lines (Figure 5).

**Figure 6:**
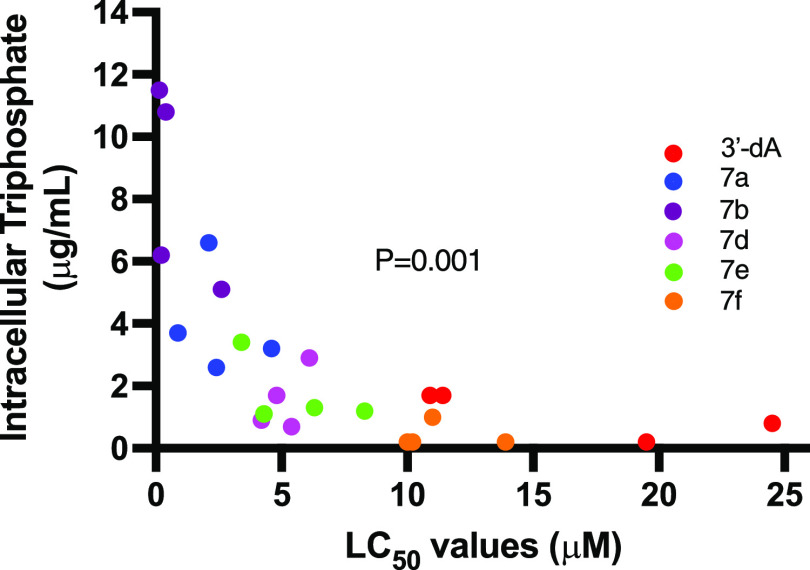
Correlation between cytotoxic LC_50_ values (μM) of 3′-dA and compounds **7a,b, d–f** and their intracellular 3′-dATP concentrations (μg/mL). There was a strong relationship between intracellular triphosphate concentration and LC_50_ values.

Figure 6 shows the correlation between the cytotoxic activity (LC_50_ values) of 3′-dA and ProTides **7a,b, d–f**, and the intracellular levels of 3′-dATP measured. A strong association between the *in vitro* potency of each agent and the level of intracellular anabolite (3′-dATP) was evident from these studies.

**Figure 7:**
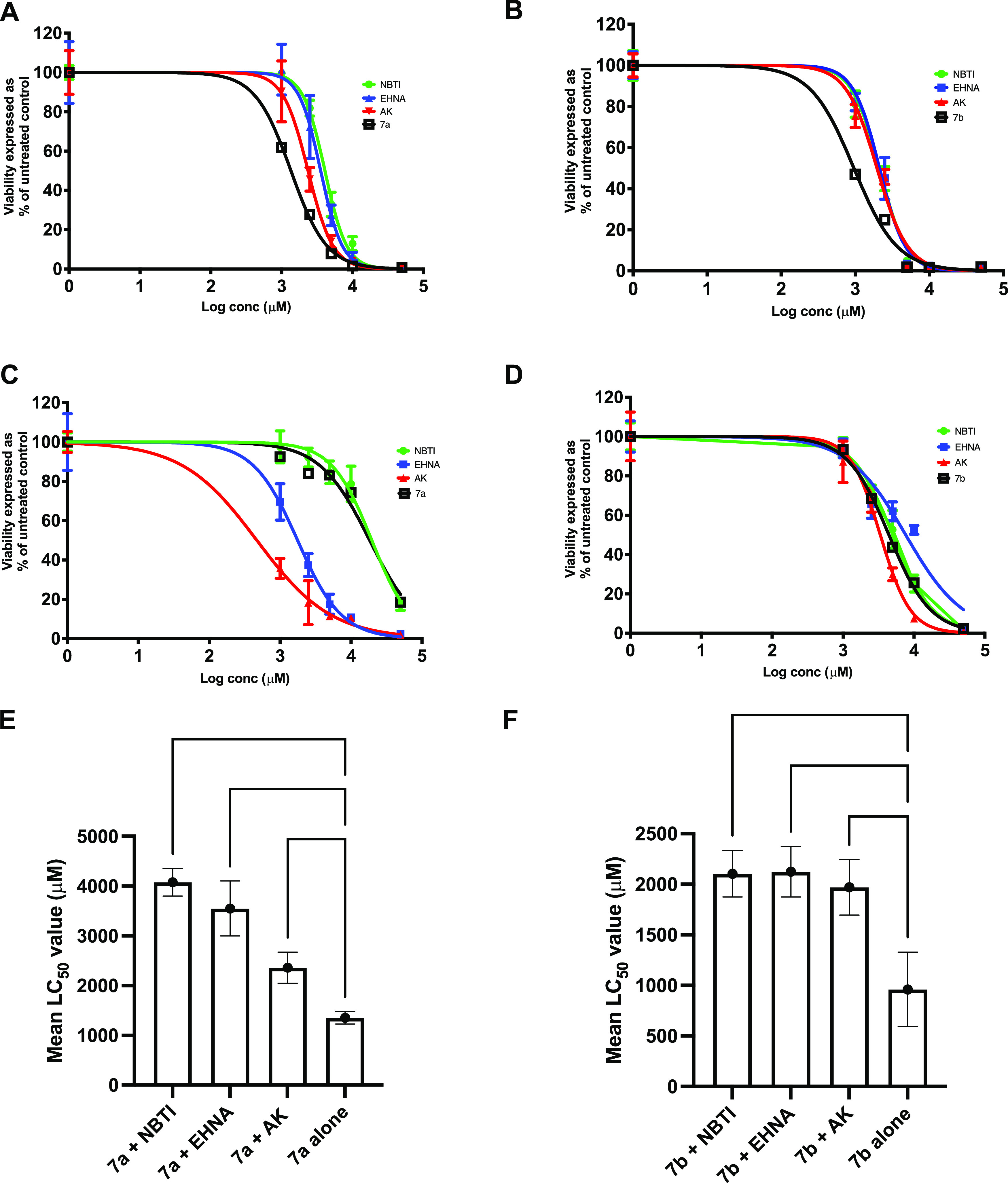
Comparative cell viability assay of ProTides **7a** and **7b** in (A, B) CEM, and (C,D) CRL in the presence of hENT1, AK, and ADA inhibitors. (E, F) Comparative LC_50_ values for **7a** and **7b**, respectively, in the presence and absence of hENT1, AK, and inhibitors in CEM. Statistical analysis was performed using one-way ANOVA with Tukey’s correction for multiple comparisons. **** *P* < 0.0001, ** *P* < 0.01, * *P* < 0.05.

The most active ProTides, **7a** and **7b**, were next tested for activity and 3′-dATP accumulation in the presence of hENT1, AK, and ADA inhibitors in CEM and CRL, and the results were compared with those obtained for 3′-dA (Figure 7). In the presence of either an hENT1 inhibitor (nitrobenzylthioinosine, NBTI) or an AK inhibitor (A-134974 dihydrochloride hydrate), 3′-dA cytotoxicity was decreased by 2- to 5-fold in both TdT^+ve^ (CEM) and TdT^–ve^ (CRL) cancer cell lines. Conversely, inhibition of ADA with erythro-9-(2-hydroxy-3-nonyl) adenine hydrochloride (EHNA) led to a 16-fold improvement of 3′-dA LC_50_ values in CEM and a 2-fold in CRL cell lines. These results indicate the dependency of 3′-dA on hENT1, AK, and ADA activities (Figure S2, Table S2). In contrast, experiments conducted with compounds **7a** and **7b** were independent of ADA activity as its inhibition by EHNA did not lead to any LC_50_ changes in CEM cell lines (Figure 7, Table S2). In CRL cell lines, ADA and AK inhibition led to increased **7a** cytotoxicity but did not affect **7b**. EHNA increased the intracellular 3′-dATP levels generated by 3′-dA by 38- and 19-fold in CEM and CRL cell lines, respectively (Table S2). This indicates that the 3′-dA is rapidly degraded by intracellular ADA. hENT1, AK, and ADA inhibitors did not affect the intracellular 3′-dATP levels generated by both ProTides **7a** and **7b**, which indicates that these new agents can overcome the cancer resistance mechanisms commonly associated with cordycepin (Table S2).

### Leukemic Stem Cell Selectivity

**Figure 8:**
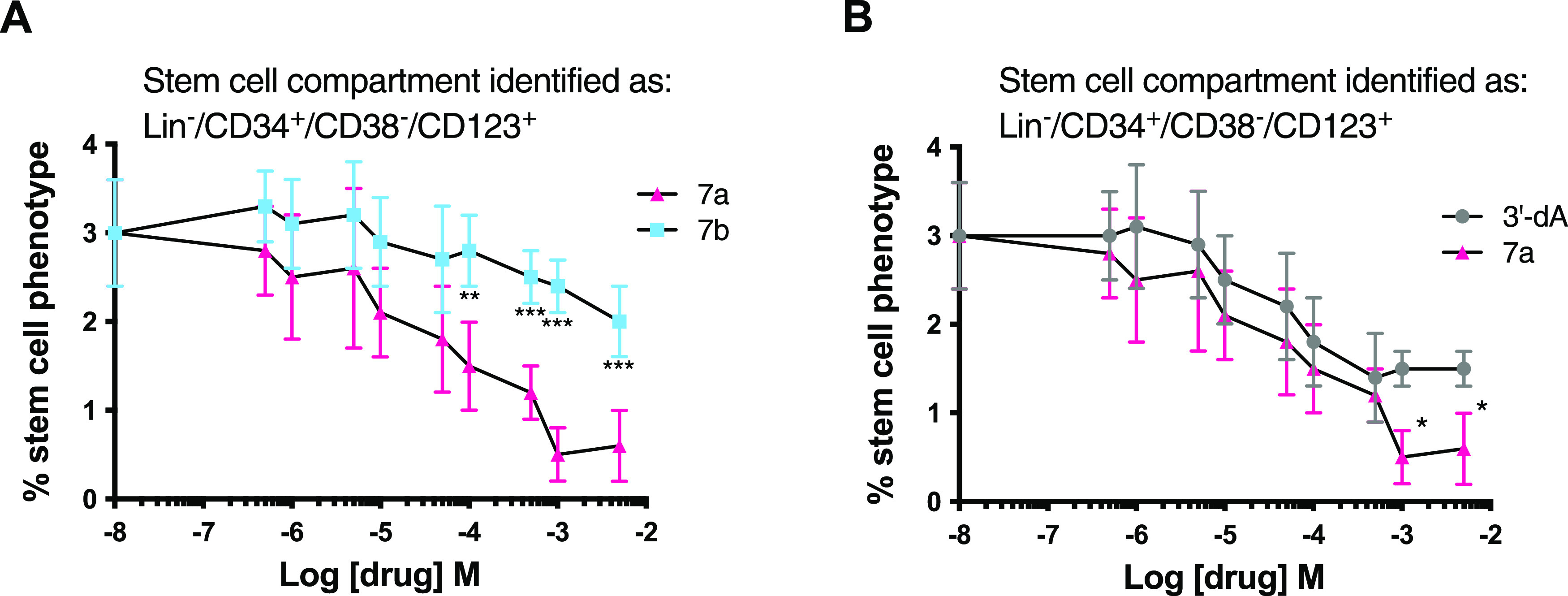
Analysis of the LSC-targeting capacity of 3′-dA and ProTides **7a** and **7b**. The LSC and bulk tumor fractions were identified using a cocktail of antibodies with LSCs: Lin^–^/CD34^+^/CD38^–^/CD123^+^ and bulk tumor: Lin^–^/CD34^+^/CD38^+^/CD123^+^. (A) Percentage of viable LSCs remaining in KG1a cell cultures following exposure to increasing concentrations of **7a** and **7b**. (B) Comparative effects on the LSC pool of 3′-dA and **7a**. All data shown are the mean ± standard deviation (SD) of three independent experiments. *** *P* < 0.001, ** *P* < 0.01, * *P* < 0.05.

We have recently reported on the preferential targeting of leukemic stem cells (LSCs) by the gemcitabine ProTide, NUC-1031, in the acute myeloid leukemia cell line KG1a and in primary AML blasts.^62^ Therefore, once we evaluated the LC_50_ of ProTide **7a** and **7b** in KG1a cells (Table S2), we went on to assess the relative ability of ProTides **7a** and **7b** to preferentially kill the LSCs compared to the bulk tumor within the KG1a cell line (LSCs were defined by the phenotype Lin^–^/CD34^+^/CD38^–^/CD123^+^).^67^ While ProTide **7b** did not lead to any significant alteration of the proportion of LSCs across the entire range of concentrations, 3′-dA and ProTide **7a** showed increased selectivity and potency against the putative leukemic stem cells with **7a** significantly reducing the LSC population to a greater extent than the parent nucleoside at concentrations above 1 μM (Figure 8).

### *In Vitro* Metabolic Stability Study

#### Stability in Human Hepatocytes

The metabolic stabilities of 3′-dA ProTides **7a,b,d−f** were investigated in human hepatocytes to assess their intrinsic clearances. The results, reported in Table 5, indicate that compounds **7a** and **7b** have similar half-lives when incubated with human hepatocytes, suggesting that they are processed at the same rate by mammalian carboxylesterases (CES1 and CES2), the crucial enzymes involved in the metabolism of endogenous esters and ester or amide-containing xenobiotics.^68^

**Table 5:**
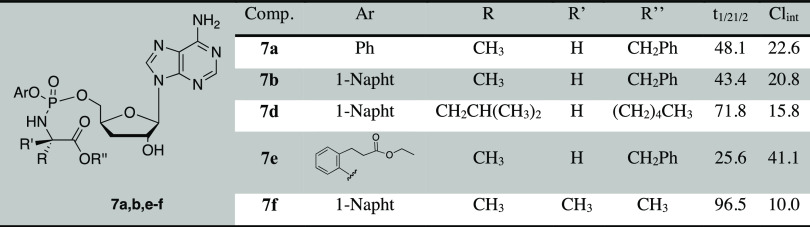
Metabolic Stability of ProTides **7a,b**, **7d–f** in Human Hepatocytes^a^

a Half-life (*t*_1/2_) expressed in minutes; intrinsic clearance (CL_int_) expressed as mL/min/10^6^ Cell.

In contrast, compound **7e** was almost 2-fold less stable (half-life = 26 min) most probably due to the presence of an additional ester moiety at the ortho position of the phenyl ring. Compounds **7d** and **7f**, bearing an amino acid other than l-alanine, resulted in longer half-lives than the other ProTides, suggesting that the nature of the amino acids plays a role in the carboxylesterase enzyme substrate recognition.

#### Deamination by ADA

**Figure 9:**
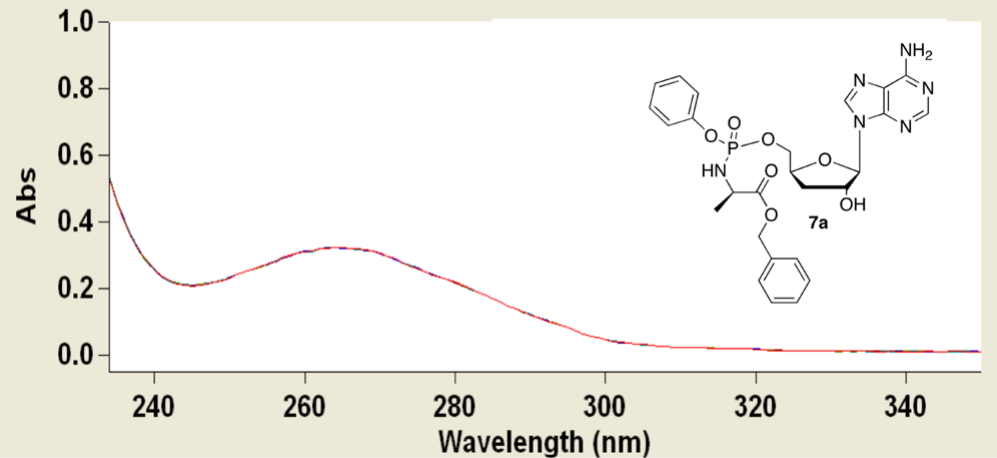
UV absorbance spectra of **7a** (62.5 μM) in the absence (red line) and presence (blue line) of ADA from calf (17 mU/mL^–1^) in phosphate buffer (100 mM, pH 7.4) at 25 °C for 12 h (the two lines are superimposed).

Enzymatic evaluation of the stability of compound **7a** versus 3′-dA in the presence of ADA was then investigated.^69^ As depicted in Figure 9, no shift of the maximum absorption at 265 nm of **7a** was observed up to 2 h from the addition of the enzyme solution, confirming the stability of **7a** to deamination. In the same condition, 3′-dA showed metabolic deamination completed within 2 minutes from the enzyme addition as evidenced by a shift of the maximum absorption at 259 nm to the maximum absorption at 252 nm, characteristic of the metabolite 3′-dIno (Figure S3).

#### Stability in Plasma

**Figure 10:**
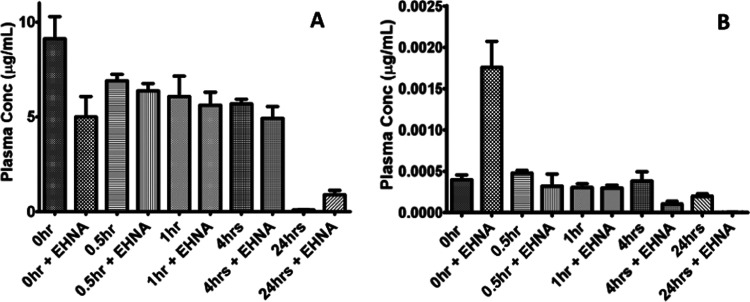
Stability assay of ProTide **7a** in human plasma (A) and mouse plasma (B).

The stability of 3′-dA and compound **7a** was assayed in human, dog, mouse, and rat plasma, with or without the presence of EHNA as an ADA inhibitor. When 3′-dA was incubated in human plasma (without the addition of EHNA), the concentration of the compound decreased in a time-dependent manner, until the nucleoside was completely metabolized after 4 h with an estimated half-life of 62 min. When EHNA was added, the stability of 3′-dA was significantly increased, with a 42.1% of 3′-dA remaining after 24 h (Figure S4A). The half-life of 3′-dA was calculated to be 252 min under these circumstances. The metabolism of 3′-dA in mouse plasma, with and without ENHA, followed a similar trend although the nucleoside was metabolized more quickly (*t*_1/2_ = 25 min) (Figure S4B). The incubation of ProTide **7a** in human plasma showed increased stability compared to the parent nucleoside, with no change in plasma concentration up to 4 h. Moreover, the addition of EHNA did not significantly affect the compound stability (Figure 10A). The stability of 3′-dA and **7a** in dog plasma (Figure S5B) was like those found in human plasma (Figure 10A). On the contrary, ProTide **7a** undergoes rapid metabolism in mouse plasma (Figure 10B). In fact, the concentration of this compound was 4 logs lower (0.0005 μg/mL) than that detected in human plasma (10 μg/mL) after just 30 s of incubation. Moreover, the presence of EHNA did not significantly affect these results, suggesting that the compound is not metabolized by ADA but through other pathways, potentially involving esterase enzymes. It was not possible to estimate the half-life of **7a** under these conditions. Similar results to mice plasma (Figure 10B) were obtained in rat plasma (Figure S5A). In fact, higher levels of carboxylesterase enzymes were reported in rodents^70^ compared to human, monkey, and dogs, and were suggested to be responsible for the reduced stability of ProTides in *in vivo* mouse xenografts.^33,71^

### Prodrug Activation by Carboxypeptidase

To exert their cytotoxic activity, ProTides must be metabolized intracellularly to produce the free monophosphate form, which in turn generates the active triphosphate after two consecutive phosphorylation reactions. In the process of intracellular activation of ProTides, the first step is catalyzed by a carboxyesterase-type enzyme such as Cathepsin A, which was shown to be responsible for the cleavage of the amino acid ester moiety.^72^ To prove that the ProTides of 3′-dA are activated in a similar manner, the interaction of compound **7a** with a carboxyesterase-type enzyme was investigated. Carboxypeptidase Y was used as a surrogate of Cathepsin A since it belongs to the same family of C-type carboxypeptidases, and it was reported to share similarities in the active site.^73^

**Figure 11:**
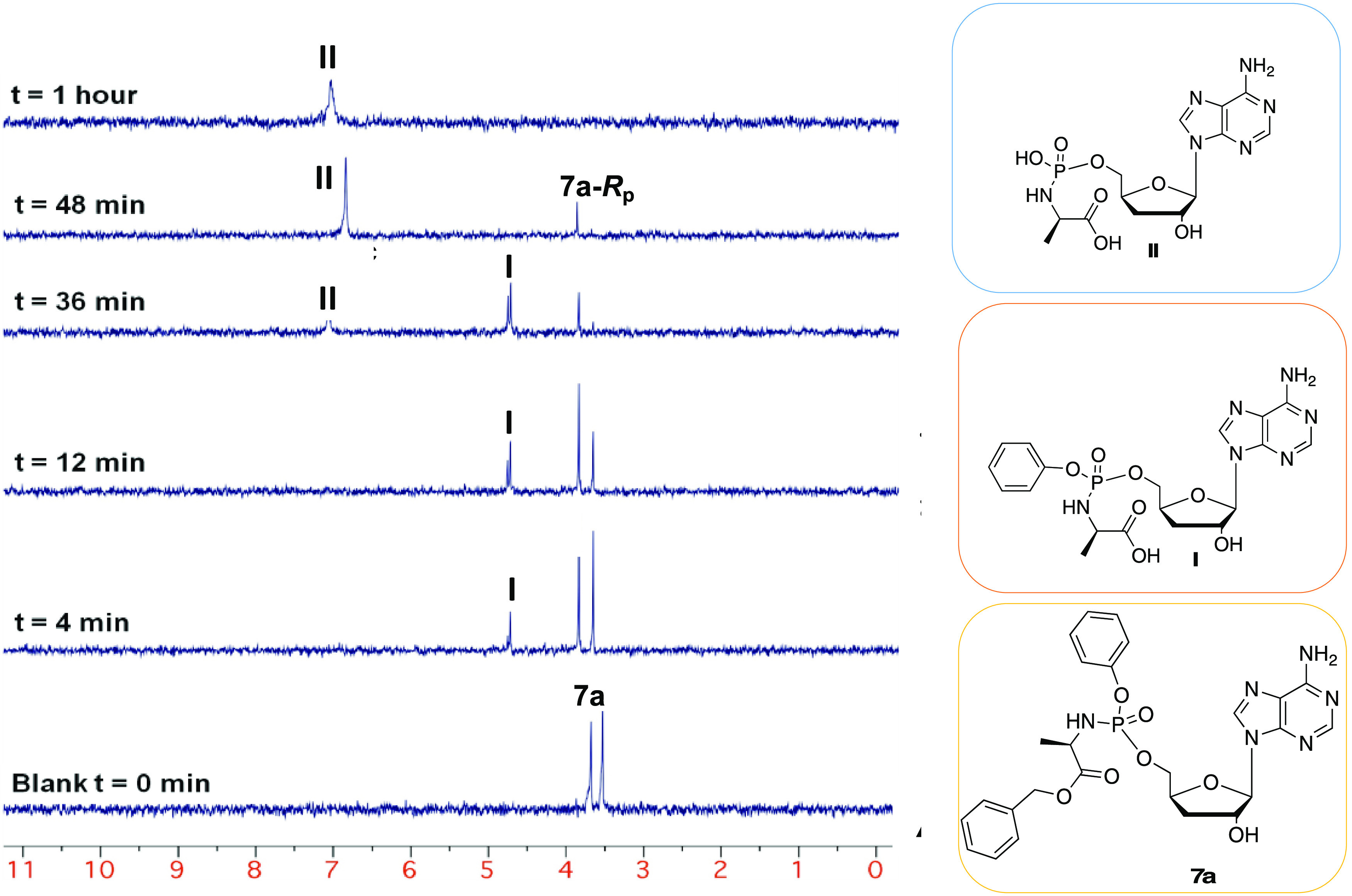
Deconvoluted stacked ^31^P-NMR spectra (202 MHz, acetone-*d*_6_/pH 7.6 Trizma buffer) to show the carboxypeptidase-mediated activation of compound **7a** to metabolite **II** over 1 h.

Compound **7a** was incubated in an NMR tube with carboxypeptidase Y in acetone-*d*_6_ in the presence of Trizma buffer (pH 7.6), and the progress of the reaction was monitored by ^31^P NMR analyses over 1 h. The stacked spectra, reported in Figure 11, show the formation of two new peaks after 4 min of incubation that correspond to the two diastereoisomers **of** intermediate **I** (**δ**_P_ 4.62 and 4.65 ppm, *t* = 4 min). A complete conversion of the ProTide **7a** into the corresponding aminoacyl phosphoramidate ester **II** (**δ**_P_ 6.89 ppm) was observed after 1 h. The *S*p diastereoisomer appeared to be converted at a slightly higher rate compared to the *R*p diasteroiosmer. *In vivo*, the aminoacyl phosphoramidate ester metabolite **II** is then believed to undergo P–N bond cleavage, mediated by HINT-1, a phosphoramidase-type enzyme, to eventually release the parental drug in its monophosphate form.^74^

## Conclusions

A series of phosphoramidate prodrugs of 3′-dA were synthesized and then evaluated for their biological activity against a range of human tumor cell lines. ProTide **7a** and **7b** showed potent cytotoxic activity against a panel of hematological cancer cell lines. Importantly, we demonstrated that both compounds were able to bypass the premature breakdown, the poor uptake, and the kinase dependence for phosphorylation of the parental nucleoside. Compound **7a** (NUC-7738) also showed selective potency against leukemic stem cells, and so it was selected as a clinical candidate drug, which is now the subject of a phase 1/2 clinical trial, NuTide:701, to establish the recommended randomized phase 2 dose and assess safety in patients with advanced solid tumors or lymphoma.^37^

Encouraging PK data from the ongoing phase I/II trial have been already reported, showing that the ProTide NUC-7738 is resistant to ADA degradation and is capable of releasing active 3′-dAMP into cells where it is rapidly converted to the active species 3′-dATP.^34^ Preliminary findings from these studies suggest that NUC-7738 is well tolerated and shows encouraging signals of anticancer activity.^34^ Taken together, our data show that NUC-7738 overcomes the key cancer resistance mechanisms that limit the efficacy of 3′-dA and support the further clinical evaluation of NUC-7738 as a novel anticancer drug.

## Experimental Section

### MTS Cell Viability Assay

The assay was contracted and carried out by WuXi AppTec (Shanghai) Co., Ltd. The tumor cell lines CCRF-CEM, HL-60, KG-1, MOLT-4, K562, MV4-11, THP-1, HEL92.1.7, NCI-H929, RPMI-8226, Jurkat, Z138, RL, HS445, HepG2, MCF-7, Bx-PC-3, HT29, MIA PaCa-2, and SW620 were seeded at cell densities of 0.5–100 Å and about 103 cells/well in a 96-well plate the day before drug incubation. Then, the plates were incubated for 72 h with the different concentrations of compound to be tested. After the incubation period, 50 μL of MTS was added and the tumor cells were incubated for 4 h at 37 °C. The data were read and collected by a Spectra Max 340 absorbance microplate reader. The compounds were tested in duplicate with nine serial concentrations (3.16-fold titrations with 198 μM as the highest concentration), and the data were analyzed by XLfit software.

### Adenosine 5′-Triphosphate (ATP) Viability Assay

#### Cell Culture Conditions

HL-60 (ATCC CCL-240), K562 (ATCC CCL-243), CCRF-CEM (ATCC CRM-CCL-119), and RL (ATCC CRL-2261) *leukemia* cell lines were obtained from the American Type Culture Collection (ATCC), Middlesex. HL-60 and K562 cell lines are deoxynucleotidyl transferase-negative (TdT^–ve^), whereas CCRF-CEM cell line is TdT^+ve^. They were cultured in RPMI-1640 medium (Sigma-Aldrich, U.K.), which were supplemented with 10% fetal bovine serum (FBS) (PAA Laboratories), 1% amphotericin B (5.5 mL), and 1% penicillin/streptomycin (5.5 mL) (PAA Laboratories) and grown in flasks at 37 °C in an incubator with 5% CO_2_. For the studies with inhibitors, the cells were treated with 10 μM nitrobenzylthioinosine (NBTI, hENT inhibitor) or 1 μM EHNA (EHNA hydrochloride) or A-134974 (A-134974 dihydrochloride hydrate) and left for 5 min before adding the drug. The cells were then incubated for 2 h at 37 °C with 5% CO_2_.

#### Measurement of In Vitro Apoptosis

The amount of ATP was used as a measurement of cell number and cell viability. ATP ViaLightTM plus assay kit (Lonza: Product No. LT07-121) to detect ATP in cells treated in luminescence compatible 96-well plates (initial concentration of cells was 1 × 10^4^ cells/well) with Cordycepin and ProTides at concentrations of 0, 0.1, 0.5, 1, 5, and 10 μM, followed by incubation for 72 h at 37 °C in an incubator with 5% CO_2_. For inhibitor studies, 10 μM NBTI or 1 μM EHNA or A-134974 was added and left for 5 min before adding the drugs. After incubation, 50 μL of cell lysis reagent was added to the 96-well plates to release the intracellular ATP, followed by 100 μL of ATP monitoring reagent (AMR). The luminescent values of each well were determined using FLUOstar OPTIMA microplate reader (BMG Labtech), which converted ATP into light using luciferase enzyme. Therefore, the amount of luminescence produced was directly proportional to the amount of ATP.

### Annexin V/7-AAD Cell Viability Assay

#### Cell Culture Conditions

The acute myeloid leukemia (AML) KG1a cell line was maintained in RPMI medium (Invitrogen, Paisley, U.K.) supplemented with 100 units/mL penicillin, 100 μg/mL streptomycin, and 20% FBS. Cells were subsequently aliquoted 10^5^ cells/100 μL into 96-well plates and were incubated at 37 °C in a humidified 5% carbon dioxide atmosphere for 72 h in the presence of 3′-dA and proTides at concentrations that were experimentally determined for each series of compounds. In addition, control cultures were carried out to which no drug was added. The cells were subsequently harvested by centrifugation and were analyzed by flow cytometry using the Annexin V assay. All experiments were performed in triplicate. LC_50_ values (the concentration of compound required to kill 50% of the cells in culture) were calculated by nonlinear regression modeling using GraphPad Prism software and are shown as mean ± standard error of the mean (SEM) for each replicate data set.

### 3′-dATP Quantification

#### Cell Treatment and Nucleotide Extraction

HL-60, K562, CCRF-CEM, and RL leukemic cell lines with 5 × 10^6^ cells/mL were used. Cells were treated with 1 μL of 50 μM of each 3′-dA, **7a,b, d–f** and incubated for 2 h at 37 °C with 5% CO_2_. After incubation, the cells were centrifuged (ambient, 300*g*, 5 min), the culture medium supernatants were removed, and the cell pellets were washed with 1 mL of phosphate-buffered saline (PBS) and centrifuged (ambient, 1200 rpm, 5 min). The supernatants were removed, and the pellets were reconstituted in 100 μL of PBS and 100 μL of 0.8 M perchloric acid and vortex-mixed and kept on ice for 30 min, then centrifuged (ambient, 300*g*, 5 min) and 180 μL of the supernatant was transferred to new tubes and stored at −80 °C until time of analysis. During analysis, 90 μL of the extract was transferred to the fresh tubes. Ammonium acetate (25 μL, 1 M) was added to the extract, and then neutralized by the addition of 10 μL of 10% ammonia and 5 μL of deionized water, then transferred to liquid chromatography–mass spectrometry (LC-MS) vials, and 10 μL was injected into the ultrahigh-performance liquid chromatography–tandem mass spectrometry (UPLC-MS/MS) system.

#### LC-MS/MS Analysis

The analytes were resolved using an ultraperformance liquid chromatography system (Accela UPLC, Thermo Scientific, U.K.) equipped with a Biobasic *A* × 5 μm, 50 × 2.1 mm column (Thermo Electron Corporation, Murrieta, CA) and mobile phase consisting of a mixture of 10 mM NH_4_Ac in ACN/H_2_O (30:70 v/v), pH 6.0 (A), and 1 mM NH_4_Ac in ACN/H_2_O (30:70 v/v), pH 10.5 (B). The mobile phase gradient employed comprised buffer *A* = 95% at 0–0.5 min, from 95 to 0% over 1.25 min, held at 0% for 1.75 min, from 0 to 95% over 0.1 min, ending with 95% for 2.9 min, all at a flow rate of 500 μL/min. Eluting compounds of interest were detected using a triple-stage quadrupole Vantage mass spectrometry system (Thermo Scientific, U.K.) equipped with an electrospray ion source. Samples were analyzed in the Multiple Reaction Monitoring, negative-ion modes at a spray voltage of 3000 V. Nitrogen was used as sheath and auxiliary gas at flow rates of 50 and 20 arbitrary units, respectively. Argon was used as collision gas with a pressure of 1.5 mTorr. The optimum transitional daughter ions mass and collision energy of each analyte were as follows: 3′dATP 490.1 → 392.1 (collision energy 19 V) and the internal standard ChloroATP 539.9 → 442.2 (collision energy 24 V).

### Immunophenotypic Identification of the Leukemic Stem Cells

KG1a cells were cultured for 72 h in the presence of a wide range of concentrations of 3′-dA and ProTides. Cells were then harvested, and KG1a cells were labeled with anti-CD34 (FITC), anti-CD38 (PE), and anti-CD123 (PERCP cy5). The subpopulation expressing a leukemic stem cell (LSC) phenotype was subsequently identified and was expressed as a percentage of all viable cells left in the culture. The percentages of stem cells remaining were then plotted on a dose–response graph, and the effects of the ProTides were compared with the parental nucleoside to determine if they preferentially target cancer stem cells

### Metabolic Stability

#### Cryopreserved Human Hepatocytes Assay

The assay was contracted and performed by Cerep (Seattle, WA Laboratories, 15318 N. E. 95th Street Redmond, WA, 98052) according to the published procedure (Cerep ref 1432). Pooled cryopreserved hepatocytes were thawed, washed, and resuspended in Krebs–Heinslet buffer (pH 7.3). The reaction was initiated by adding the test compound (1 μM final concentration) into cell suspension and incubated in a final volume of 100 μL on a flat-bottom 96-well plate for 0 and 60 min, respectively, at 37 °C/5% CO_2_. The reaction was stopped by adding 100 μL of acetonitrile into the incubation mixture. Samples were then mixed gently and briefly on a plate shaker, transferred completely to a 0.8 mL V-bottom 96-well plate, and centrifuged at 2550*g* for 15 min at room temperature. Each supernatant (150 μL) was transferred to a clean cluster tube, followed by high-performance liquid chromatography–tandem mass spectrometry (HPLC-MS/MS) analysis on a Thermo Electron triple quadrupole system.

### Plasma Stability

Plasma samples (10 mL of human, beagle dog, mouse, or rats from Sera laboratories, U.K.) were treated with and without 1 μM of EHNA, after which 20 μM concentration of each drug (3′dA and ProTides) was added and incubated for 2 h at 37 °C with 5% CO_2_. After incubation, 100 μL of each sample was taken and 300 μL of 100% methanol was added and left on ice for 30 min. After 30 min, the mixture was centrifuged (14 000 rpm, 4 °C, 10 min), and the supernatant was transferred to new tubes and evaporated under SpeedVac. Then, the dried extract was reconstituted in 100 μL of 10% acetonitrile, then transferred to LC-MS vials, and 10 μL was injected into the UPLC-MS/MS system.

### Adenosine Deaminase Assay

Bovine recombinant adenosine deaminase (ADA, EC:3.5.4.4) was purchased from Sigma (Sigma-Aldrich, product number 59722). The ADA suspension (containing >2 U/mL) was aliquoted into 55 μL portions and kept at −20 °C. Before use, a 55 μL sample was thawed and 1 mL of phosphate buffer (0.1 N, pH 7.4) was added. The suspension was filtered through a 0.2 μm filter to remove precipitates that could interfere with the absorbance measurements. The clear supernatant was used for the degradation experiments. The experiments were conducted in UV transparent cuvettes (Sigma-Aldrich, code: Z637092) using an Envision microplate reader (PerkinElmer). Samples contained 62.5 μM of either 3′-dA or ProTide **7a**, which were added to 17 μL of the ADA solution. Scans of the absorption spectrum were taken between 220 and 350 nm with 2 nm resolution every 0.1 min over a period of 10 min to 12 h. One sample containing only ADA was also prepared, and this was subtracted to obtain the spectrum over time for substrates.

### Carboxypeptidase Y Assay

ProTides **7a** (5 mg, ±0.008 mmol) was dissolved in 150 μL of acetone-*d*_6_, and 300 μL of trizma buffer (pH 7.6) was added. A ^31^P NMR (202 MHz, 64–128 scans) was conducted at this stage as a reference (blank, *t* = 0). To this mixture, 130 μL of a stock solution of carboxypeptidase enzyme (purchased from Sigma-Aldrich, >50 unit/mg, dissolved in trizma buffer 7.6 pH to a concentration of 50 units/mL, EC 3.4.16.1) was added. ^31^P NMR (128 scans) were carried out with 1 min of delay between experiments for 14 h at 25 °C.

### Chemistry

#### General Information

All solvents used were anhydrous and used as supplied by Sigma-Aldrich. All commercially available reagents were supplied by either Sigma-Aldrich or Fisher and used without further purification. All solid reagents were dried for several hours under high vacuum prior to use. For analytical thin-layer chromatography (TLC), precoated aluminum-backed plates (60 F-54, 0.2 mm thickness; supplied by E. Merck AG, Darmstadt, Germany) were used and developed by an ascending elution method. For preparative thin-layer chromatography (prep TLC), preparative TLC plates (20 cm × 20 cm, 500–2000 μm) were purchased from Merck. After solvent evaporation, compounds were detected by quenching of the fluorescence at 254 nm upon irradiation with a UV lamp. Column chromatography purifications were carried out by means of automatic Biotage Isolera One. Fractions containing the product were identified by TLC and pooled, and the solvent was removed in vacuo. ^1^H, ^31^P, and ^13^C NMR spectra were recorded in a Bruker Avance 500 spectrometer at 500, 202, and 125 MHz, respectively, and autocalibrated to the deuterated solvent reference peak in case of ^1^H and ^13^C NMR and 85% H_3_PO_4_ for ^31^P experiments. All ^31^P and ^13^C NMR spectra were proton-decoupled. Chemical shifts are given in parts per million (ppm), and coupling constants (*J*) are measured in hertz (Hz). The following abbreviations are used in the assignment of NMR signals: s (singlet), d (doublet), t (triplet), q (quartet), m (multiplet), bs (broad singlet), dd (doublet of doublet), ddd (doublet of doublet of doublet), dt (doublet of triplet). The assignment of the signals in ^1^H NMR and ^13^C NMR was done based on the analysis of coupling constants and additional two-dimensional experiments (correlation spectroscopy (COSY), heteronuclear single-quantum coherence (HSQC)). Analytical high-performance liquid chromatography (HPLC) analysis was performed using both Spectra System SCM (with X-select-C18, 5 mm, 4.8 × 150 mm column) and Varian Prostar system (LCWorkstation-Varian Prostar 335 LC detector). Preparative HPLC was performed with Varian Prostar (with pursuit XRs C18 150 × 21.2 mm column). Low-resolution mass spectrometry was performed on a Bruker Daltonics MicroTof-LC system (atmospheric pressure ionization, electron spray mass spectroscopy) in positive mode. The ≥95% purity of the final compounds **5a**, **6a**, **5b**, **6b**, **7a**, **7b**, **7c**, **7e**, and **7f**, and the 93% purity for **7d** were confirmed using HPLC analysis.

##### 9-(2′,3′-Anhydro-α-d-ribofuranosyl) Adenine (**3**)

To a stirring suspension of adenosine (**2**) (10.00 g, 37.42 mmol) in CH_3_CN (250 mL), α-AIBBr (22.03 mL, 149.68 mmol, 4 equiv) and H_2_O (0.67 mL, 0.037 mmol, 0.001 equiv) were added, and stirring was continued at room temperature. After 1 h, the mixture was neutralized by the addition of a saturated solution of NaHCO_3_ (a change in color from dark orange to clear-white could be noted), and the solution was extracted with EtOAc (2 × 5 mL per mmol of adenosine analogue). The combined organic phase was washed with brine (1 mL × mmol of adenosine analogue). The aqueous phase was extracted with EtOAc (1 × 190 mL), and the combined organic phase was dried over Na_2_SO_4_, filtered, and evaporated to give a white gum. The crude mixture was dissolved in CH_3_OH (260 mL) and stirred for 16 h with Amberlite (2 × OH) resin (150 mL, 1.1 meq/mL by wetted bed volume), previously washed with CH_3_OH. The solution was then filtered, and the resin was carefully washed with CH_3_OH until no spot of the product by TLC could be detected in the filtrate. Evaporation of the combined filtrate and crystallization of the residue from EtOH gave **3** as a white powder (8.86 g, 95%). Melting point 178–180 °C (Lit. mp: 180–181 °C). ^1^H NMR (500 MHz, DMSO-*d*_6_) δ_H_ 8.33 (s, 1H, H-2), 8.18 (s, 1H, H-8), 7.29 (br s, 2H, NH_2_), 6.21 (s, 1H, H-1′), 5.05 (br s, 1H, OH-5′), 4.46 (d, *J* = 2.6 Hz, 1H, H-2′), 4.22 (d, *J* = 2.6 Hz, 1H, H-3′), 4.18 (t, *J* = 5.2 Hz, 1H, H-4′), 3.60–3.55 (m, 1H, H-5′), 3.53–3.49 (m, 1H, H-5′). ^13^C NMR (125 MHz, DMSO-*d*6) δ_C_ 156.01 (C-6), 152.61 (C-2), 149.11 (C-4), 139.55 (C-8), 119.52 (C-5), 91.25 (C-1′), 81.15 (C-4′), 75.06 (C-2′), 58.75 (C-3′), 57.70 (C-5′). HPLC reversed-phase HPLC eluting with H_2_O/CH_3_CN from 100/0 to 75/25 in 30 min, *F* = 1 mL/min, λ = 254 nm, showed one peak with *t*_R_ 13.20 min. C_10_H_13_N_5_O_3_ required *m*/*z* 249.23 [M]; (ES+) found *m*/*z* 272.09 [M + Na]^+^, 250.09 [M + H]^+^.

##### 3′-Deoxyadenosine (**1**)

2′,3′-Anhydrous adenosine **3** (9.12 g, 36.59mmol, 1 equiv) was dissolved in a mixture of DMSO (55 mL) and THF (550 mL), under argon atmosphere. The solution was cooled down to 0 °C and 1 M LiEt_3_BH in THF (110 mL, 109.77 mmol, 3 equiv) was added dropwise. Stirring was continued at ∼4 °C for 1 h and at rt for 16 h. The mixture was cooled down to 0 °C, and an additional portion of 1 M LiEt_3_BH in THF (36.6 mL, 36.6 mmol, 1 equiv) was added dropwise. The mixture was stirred at 0 °C for 1 h and then at rt for 1 h. The reaction mixture was carefully acidified (5% AcOH/H_2_O), purged with N_2_ for 1 h (under the fume hood) to remove *pyrophoric* triethylborane, and evaporated. Purification by silica gel flash column chromatography (eluent system CH_3_OH/CH_2_Cl_2_ 5/95 to 20/80) afforded the title compound **1** as a white solid (9.01 g, 98%). Melting point: 188–190 °C (Lit. mp: 191–192 °C). ^1^H NMR (500 MHz, DMSO-*d*_6_) δ_H_ 8.37 (s, 1H, H-8), 8.17 (s, 1H, H-2), 7.29 (br s, 2H, NH_2_), 5.89 (d, *J* = 2.5 Hz, 1H, H-1′), 5.68 (d, *J* = 4.5 Hz, 1H, OH-2′), 5.19 (t, *J* = 6.0 Hz, 1H, OH-5′), 4.63–4.58 (m, 1H, H-2′), 4.40–4.34 (m, 1H, H-4′), 3.71 (ddd, *J* = 12.0, 6.0, 3.0 Hz, 1H, H-5′), 3.53–3.49 (ddd, *J* = 12.0, 6.0, 4.0 Hz, 1H, H-5′). ^13^C NMR (125 MHz, DMSO-*d*_6_) δ_C_ 156.00 (C-6), 152.41 (C-2), 148.82 (C-4), 139.09 (C-8), 119.06 (C-5), 90.79 (C-1′), 80.66 (C-4′), 74.56 (C-2′), 62.61 (C-5′), 34.02 (C-3′). C_10_H_13_N_5_O_3_ required *m*/*z* 251.24 [M]. (ES+) found *m*/*z* 258.12 [M + Li]^+^, 274.09 [M + Na]^+^, 252.11 [M + H]^+^. HPLC reversed-phase eluting with H_2_O/CH_3_CN from 100/0 to 75/25 in 30 min, *F* = 1 mL/min, λ = 254 nm, *t*_R_ 11.22 min.

#### Standard Procedure A for the Synthesis of Aryl Amino Acid Ester Phosphorochloridates from Amino Acid Hydrochloride or p-Toluene Sulfonate Salts

The appropriate amino acid ester salt (1 equiv) was dissolved in CH_2_Cl_2_ (4 mL per mmol of amino acid ester) under argon atmosphere. A solution of the appropriate phosphorodichloridate (1 equiv) in CH_2_Cl_2_ (1 mL per mmol of amino acid ester) was added, and the mixture was cooled to −78 °C. Et_3_N (2 equiv) was added dropwise, and the reaction mixture was stirred at −78 °C for 20 min and thereafter at rt for 2.5 h. The solvent was evaporated under reduced pressure, the resulting oil was triturated with anhydrous Et_2_O, and the filtrate was reduced to give the crude product as an oil.

##### Phenyl-(benzoxy-l-alaninyl)dichlorophosphate (**4a**)

Prepared according to the general procedure **A** using L-alanine benzyl ester hydrochloride salt (1.12 g, 5.20 mmol), phenyl dichlorophosphate (1.10 g, 5.20 mmol), and Et_3_N (1.45 mL, 10.40 mmol) in CH_2_Cl_2_ (20 mL). The product was obtained as a clear oil (1.51 g, 82%).^31^P NMR (202 MHz, CDCl_3_) δ_P_ 7.51, 7.85. ^1^H NMR (500 MHz, CDCl_3_) δ_H_ 7.36–7.25 (m, 10 H, Ar), 5.15 (s, 1H, C*H*_2_Ph), 5.11 (s,1H, C*H*_2_Ph), 4.25–4.14 (m, 2H, NH, C*H*CH_3_), 1.59–1.57 (m, 3H, CHC*H*_3_).

#### Standard Procedure B for the Synthesis of ProTides **5a**,**b** and **6a**,**b**

##### 1

3′-Deoxyadenosine () (1 equiv) was dissolved in THF (20 mL per 0.1 mmol of nucleoside) under argon atmosphere. 1 M *t*BuMgCl in THF (1.1 equiv) was added dropwise. The appropriate phosphorochloridate (**4a**,**b**) (3 equiv) was dissolved in THF (4 mL per 1 mmol of phosphorochloridate), and the resulting solution was added to the initial mixture. The mixture was stirred for 12–16 h, and the solvent was evaporated under vacuum. The obtained crude was purified by silica gel CC or Biotage Isolera One. In some cases, further purification by preparative TLC and/or preparative HPLC was necessary.

#### Standard Procedure C for the Synthesis of ProTides **7a–f**

##### 1

3′-Deoxyadenosine () (1 equiv) was dissolved in THF (7 mL per 0.1 mmol of nucleoside) under argon atmosphere. The appropriate phosphorochloridate (**4a–f**) (3 equiv) was dissolved in THF (4 mL per 1 mmol of phosphorochloridate), and the resulting solution was added to the initial mixture, followed by NMI (5 equiv). The mixture was stirred for 12–16 h, and the solvent was evaporated under vacuum. The obtained crude was purified by silica gel CC or Biotage Isolera One. In some cases, further purification by preparative TLC and/or preparative HPLC was necessary.

##### (*R*_p_)- and (*S*_p_)-3′-Deoxyadenosine 5′-*O*-phenyl-(benzoxy-l-alaninyl)-phosphate (**7a**)

Prepared according to general procedure **C** using 3′-deoxyadenosine (**1**) (0.05 g, 0.20 mmol) in anhydrous THF (4 mL), *N*-methyl imidazole (0.080 μL, 1.0 mmol), and phenyl(benzyloxy-l-alaninyl) phosphorochloridate (**4a**) (0.021 g, 0.6 mmol) in THF (2.4 mL) Purification by Biotage Isolera One (cartridge SNAP 25 g, 25 mL/min, CH_3_OH/CH_2_Cl_2_ 1–8% 10 CV, 8% 5 CV) and preparative TLC (1000 μM, eluent system CH_3_OH/CH_2_Cl_2_ 5/95) afforded the title compound **7a** as a white solid (0.032 g, 28%). ^31^P NMR (202 MHz, CD_3_OD) δ_P_ 3.91, 3.73. ^1^H NMR (500 MHz, CDCl_3_) δ_H_ 8.26 (s, 0.5H, H-8), 8.24 (s, 0.5H, H-8), 8.22 (s, 0.5H, H-2), 8.21 (s, 0.5H, H-2), 7.34–7.25 (m, 7H, Ar), 7.21–7.13 (m, 3H, Ar), 6.01 (d, *J* = 1.5 Hz, 0.5H, H-1′), 6.00 (d, *J* = 1.5 Hz, 0.5H, H-1′), 5.15–5.04 (m, 2H, C*H*_2_Ph), 4.73–4.63 (m, 2H, H-2′, H-4′), 4.43–4.35 (m, 1H, H-5′), 4.27–4.20 (m, 1H, H-5′), 4.03–3.91 (m, 1H, C*H*CH_3_), 2.35–2.28 (m, 1H, H-3′), 2.09–2.02 (m, 1H, H-3′), 1.32 (d, *J* = 7.4 Hz, 1.5 H, CHC*H*_3_), 1.28 (d, *J* = 7.4 Hz, 1.5 H, CHC*H*_3_). ^13^C NMR (125 MHz, CD_3_OD) δ_C_ 174.84 (d, ^3^*J*_C-P_ = 4.5 Hz, C=O), 174.63 (d, ^3^*J*_C-P_ = 4.5 Hz, C=O), 157.32 (C-6), 157.31 (C-6), 153.86 (C-2), 153.84 (C-2), 152.13 (C-4), 152.07 (C-4), 150.20 (C-Ar), 150.18 (C-Ar), 140.47 (C-8), 137.26 (C-Ar), 137.19 (C-Ar), 130.76 (CH-Ar), 130.74 (CH-Ar), 129.57 (CH-Ar), 129.32 (CH-Ar), 129.31 (CH-Ar), 129.29 (CH-Ar), 129.26 (CH-Ar), 126.16 (CH-Ar), 126.14 (CH-Ar), 121.46 (d, ^3^*J*_C-P_ = 4.7 Hz, CH-Ar), 121.38 (d, ^3^*J*_C-P_ = 4.7 Hz, CH-Ar) 120.54 (C-5), 120.53 (C-5), 93.24 (C-1′), 93.18 (C-1′), 80.43 (d, ^3^*J*_C-P_ = 3.6 Hz, C-4′), 80.36 (d, ^3^*J*_C-P_ = 3.6 Hz, C-4′), 76.62 (C-2′), 68.62 (d, ^2^*J*_C-P_ = 5.3 Hz, C-5′), 68.30 (d, ^2^*J*_C-P_ = 5.3 Hz, C-5′), 67.95 (*C*H_2_Ph), 67.92 (*C*H_2_Ph), 51.74 (*C*HCH_3_), 51.60 (*C*HCH_3_), 34.91 (C-3′), 34.70 (C-3′), 20.45 (d, ^3^*J*_C-P_ = 7.0 Hz, CH*C*H_3_), 20.28 (d, ^3^*J*_C-P_ = 7.0 Hz, CH*C*H_3_). Reversed-phase HPLC eluting with H_2_O/CH_3_CN from 100/10 to 0/100 in 30 min, *F* = 1 mL/min, λ = 254 nm, *t*_R_ 13.56 and 13.75 min. C_26_H_29_N_6_O_7_P required *m*/*z* 568.2 [M]. MS (ES+) found *m*/*z* 569.2 [M + H]^+^, 591.2 [M + Na]^+^, 1159.4 [2M+Na]^+^.

The two diastereoisomers **7a-*****R***_**p**_ and **7a-*****S***_**p**_ were separated via Biotage Isolera One (cartridge SNAP-Ultra C18 12 g, *F*: 12 mL/min, isocratic eluent system: H_2_O/CH_3_OH 45/55 in 30 min, 150 mg sample) to obtain:

##### **7a-*****R***_**p**_ as Fast Eluting Isomer (76 mg)

^31^P NMR (202 MHz, CD_3_OD) δ_P_ 3.91. ^1^H NMR (500 MHz, CDCl_3_) δ_H_ 8.26 (s, 1H, H-8), 8.22 (s, 1H, H-2), 7.37–7.25 (m, 7H, Ar), 7.22–7.12 (m, 3H, Ar), 6.01 (d, *J* = 1.5 Hz, 1H, H-1′), 5.12 (AB q, *J*_AB_ = 12.0 Hz, 2H, C*H*_2_Ph), 4.74–4.70 (m, 1H, H-2′), 4.69–4.62 (m, 1H, H-4′), 4.44–4.38 (m, 1H, H-5′), 4.28–4.21 (m, 1H, H-5′), 3.99–3.90 (m, 1H, C*H*CH_3_), 2.35–2.27 (m, 1H, H-3′), 2.09–2.02 (m, 1H, H-3′), 1.29 (d, *J* = 7.0 Hz, 3H, CHC*H*_3_). HPLC reversed-phase HPLC eluting with H_2_O/CH_3_CN from 90/10 to 0/100 in 30 min, *F* = 1 mL/min, λ = 254 nm, showed one peak with *t*_R_ 13.56 min.

##### **7a-*****S***_**p**_ as Slow-Eluting Isomer (61 mg)

^31^P NMR (202 MHz, CD_3_OD) δ_P_ 3.73. ^1^H NMR (500 MHz, CDCl_3_) δ_H_ 8.24 (s, 1H, H-8), 8.22 (s, 1H, H-2), 7.36–7.26 (m, 7H, Ar), 7.22–7.13 (m, 3H, Ar), 6.01 (d, *J* = 1.5 Hz, 1H, H-1′), 5.08 (AB q, *J*_AB_ = 12.0 Hz, 2H, C*H*_2_Ph), 4.70–4.67 (m, 1H, H-2′), 4.66–4.60 (m, 1H, H-4′), 4.41–4.35 (m, 1H, H-5′), 4.26–4.19 (m, 1H, H-5′), 4.02–3.94 (m, 1H, C*H*CH_3_), 2.36–2.27 (m, 1H, H-3′), 2.08–2.01 (m, 1H, H-3′), 1.34–1.30 (m, 3H, CHC*H*_3_). HPLC reversed-phase HPLC eluting with H_2_O/CH_3_CN from 90/10 to 0/100 in 30 min, *F* = 1 mL/min, λ = 254 nm, *t*_R_ 13.75 min.

## Supporting Information Available

Antiproliferative activity of 3′-dA and compounds **5a,b** and **6b** (Table S1); antiproliferative activity of compounds **7a,b, d–f** in CEM, K562, HL60, CRL cell lines (Table S2); antiproliferative activity and 3′-dATP level of 3′-dA and compounds **7a,b** in the presence of NBTI, EHNA, and A-134974 dihydrochloride hydrate in CEM and CRL cell lines (Table S3); analytical HPLC traces of **7a** diastereomeric mixture (Figure S1); in vitro cytotoxicity of 3′-dA in the presence/absence of hENT3, AK, and ADA blockers in CEM and CRL cell lines (Figure S2); change in UV absorbance spectra of 3′-dA after addition of a solution of ADA (in phosphate buffer pH 7.4) (Figure S3); stability of 3′-dA in human and mouse plasma (Figure S4); stability of 3′-dA and **7a** in rat and dog plasma (Figure S5); intermediate and final compounds characterization description; copies of ^31^P, ^1^H, and ^13^C NMR spectra, and HPLC traces of compounds **1, 3, 5a–b**, **7a–f**, **7a-*****R*****p and 7a-*****S*****p**, and **9**; atomic coordinates and equivalent isotropic displacement parameters for *S*p-**7a** (PDF)
Molecular formula strings (CSV)

## Abbreviations Used

ADA: adenosine deaminase
ADORAs: adenosine receptors
AK: adenosine kinase
AMPK: adenosine monophosphate kinase
3′-dA: 3′-deoxyadenosine
3′-dAMP: 3′-deoxyadenosine monophosphate
3′-dATP: 3′-deoxyadenosine triphosphate
DRs: death receptors
EGFR: epidermal growth factor receptor
hENT: human equilibrative nucleoside transporters
NDPK: nucleoside diphosphate kinases
3′-dIno: 3′-deoxyinosine
PAP: polyadenylate polymerase
PRPPT: phosphoribosyl-pyrophosphate aminotransferase
