# Supplementary material for: Synthesis and Characterization of NUC-7738, an Aryloxy Phosphoramidate of 3′-Deoxyadenosine, as a Potential Anticancer Agent
Source: J Med Chem. 2022 Nov 23;65(23):15789–804. doi: 10.1021/acs.jmedchem.2c01348 (PMC9743095; doi:10.1021/acs.jmedchem.2c01348)
Supplement: Supplementary file 1 — jm2c01348_si_001.pdf [file jm2c01348_si_001.pdf]

Supporting Information

Synthesis and characterisation of NUC-7738, an aryloxy phosphoramidate of 3´-deoxyadenosine, as a potential anti-cancer agent

Michaela Serpi,[1*] Valentina Ferrari[2], Christopher McGuigan[2] Essam Ghazaly[3], Chris Pepper[4]

[1]School of Chemistry, Cardiff University Main Building, Park Place, CF10 3AT Cardiff, Wales, United Kingdom

[2]School of Pharmacy and Pharmaceutical Sciences, Cardiff University, Cardiff, King Edward VII Avenue, Cardiff, CF10 3NB, UK.

[3]Centre for Haemato-Oncology, Barts Cancer Institute, Queen Mary University of London, Charterhouse Square, London, EC1M 6BQ, UK.

[4]Brighton and Sussex Medical School, University of Sussex, BN1 9PX, UK.

Corresponding Author:

*Michaela Serpi – School of Chemistry, Cardiff University Main Building, Park Place, CF10 3AT Cardiff, Wales, United Kingdom. orcid.org/0000-0002-6162-7910; Phone: +44029208775571; Email: serpiM5@cardiff.ac.uk.

Table of contents

Table S1	p. S3
Table S2	p. S3
Table S3	p. S3
Table S4	p. S4-S5
Figure S1	p. S6
Figure S2	p. S6
Figure S3	p. S6
Figure S4	p. S7
Figure S5	p. S7
Synthesis of 9-(2',3'-Anhydro-α-D-ribofuranosyl) adenine (3)	p. S7
Synthesis of 3'deoxy adenosine (1)	p. S8
Ethyl 3-(2-hydroxyphenyl)propanoate	p. S8
Synthesis of Naphth-1-yl dichlorophosphate	p. S9
Synthesis of Ethyl 3-(2-hydroxyphenyl)propanoyl dichlorophosphate	p. S9
Synthesis of aryl amino acid ester phosphorochloridates (4a-f)	p. S10-S11
Synthesis of ProTides 5a-b and 6a-b	p. S11-S14
Synthesis of ProTides 7a-f	p. S14-S21
Copies of $^{1}H/^{31}P/^{13}C$ NMR spectra and HPLC traces	p. S22-S48

Table S1. Antiproliferative activity of 3’-dA and compounds **5a,b** and **6b**.

Comp	CCRF-CEM		HL-60		KG-1		MOLT-4		K562		HepG2		MCF-7	
	LC_{50}	$MI_{\%}$	LC_{50}	$MI_{\%}$	LC_{50}	IC_{50}	LC_{50}	$MI_{\%}$	LC_{50}	$MI_{\%}$	LC_{50}	$MI_{\%}$	LC_{50}	$MI_{\%}$
**3’-dA**	>198	12	76.84	88	70.82	78	151.92	52	59.31	88	142.47	66	34.23	78
**5a**	64.83	65	143.47	57	111.95	63	62.86	75	26.3	78	85.5	667	10.35	97
**5b**	27.14	99	78.59	99	66.28	93	14.78	100	25.35	87	30.16	90	5.05	99
**6a**	17.63	99	31.09	80	79.44	69	18.62	100	26.87	89	83.06	55	5.06	94

Cytotoxicity data reported as µM LC50 values (concentration of drug causing 50% of cell death)

Table S2. Antiproliferative activity of 3’-dA compounds **7a,b, d–f**

Comp	CEM	K562	HL-60	CRL
	**LC_{50}**	**LC_{50}**	**LC_{50}**	**LC_{50}**
**3’-dA**	19.5	10.9	11.4	24.5
**7a**	0.87	2.4	4.6	2.1
**7b**	0.13	0.21	2.6	0.4
**7d**	6.1	4.2	5.4	4.8
**7e**	4.3	6.3	8.3	3.4
**7f**	10.0	13.9	10.2	11.0

Cytotoxicity data reported as µM LC50 values (concentration of drug causing 50% of cell death)

Table S3. Antiproliferative activity of 3’-dA compounds **7a,b** in presence of NBTI (nucleoside transporter), EHNA(Adenosine deaminase inhibitor) A-134974 dihydrochloride hydrate (AK inhibitors)

Compound	Inhibitors	CEM		CRL	
		**LC_{50}**	**[3’-dATP]**	**LC_{50}**	**[3’-dATP]**
**3’-dA**	Control	11.5	0.24	7.3	0.10
	NBTI	57.7	0.14	17.9	0.06
	EHNA	0.7	9.01	13.2	1.85
	A-134974	29.2	0.31	28.6	0.16
**7a**	Control	1.4	1.30	3.6	0.31
	NBTI	2.0	0.99	2.6	0.32
	EHNA	3.1	1.35	2.9	0.27
	A-134974	3.6	1.20	10.2	0.30
**7b**	Control	0.9	4.07	3.1	0.59
	NBTI	1.4	3.14	2.6	0.68
	EHNA	1.3	1.35	5.2	0.27
	A-134974	1.3	1.20	2.8	0.30

Cytotoxicity data reported as µM LC50 values (concentration of drug causing 50% of cell death) [3’-dATP] is reported in µg/mL

Table S4: Atomic coordinates (x104) and equivalent isotropic displacement parameters (Å2x103) for *S*p-**7a**

**Atom**	***x***	***y***	***z***	**U(eq)**
C1	-5602.1(16)	5924.5(9)	429.4(7)	26.6(3)
C2	-3860.7(16)	6851.7(8)	120.5(6)	22.9(3)
C3	-2808.0(16)	6258.4(8)	273.3(7)	22.3(3)
C4	-3326.8(16)	5526.0(9)	499.5(6)	22.2(3)
C5	-959.4(16)	5519.0(9)	441.6(7)	24.6(3)
C6	-2194.6(15)	4200.6(8)	816.9(7)	20.2(3)
C7	-2319.0(15)	3619.2(8)	251.2(7)	22.5(3)
C8	-737.5(15)	3426.4(9)	123.2(6)	23.1(3)
C9	-145.9(14)	3370.8(8)	789.9(6)	20.7(3)
C10	1454.3(15)	3513.7(9)	807.7(6)	22.4(3)
C11	5180.5(17)	2776.2(8)	2432.7(7)	26.1(3)
C12	6469.5(18)	2700.8(10)	2113.0(8)	33.3(3)
C13	7720(2)	2525.8(11)	2453.3(9)	38.1(4)
C14	7666(2)	2426(1)	3095.3(9)	37.3(4)
C15	6366(2)	2498.5(10)	3405.6(8)	38.2(4)
C16	5101.3(19)	2677(1)	3077.3(8)	32.4(3)
C17	3388.6(17)	4819.7(9)	2154.9(7)	26.4(3)
C18	4375(2)	5495.9(12)	2394.8(10)	42.0(4)
C19	1979.7(17)	5194.4(8)	1909.9(7)	25.5(3)
C20	-367.8(18)	5622.5(11)	2198.7(8)	32.7(3)
C21	-1367.5(17)	5518.8(10)	2746.4(7)	29.2(3)
C22	-1963.5(18)	6185.5(10)	3047.4(7)	30.5(3)
C23	-2911.8(19)	6077.3(10)	3551.4(8)	33.0(3)
C24	-3246.3(19)	5305.5(11)	3758.4(8)	35.1(4)
C25	-2646(2)	4637.6(11)	3456.7(10)	42.4(4)
C26	-1728(2)	4743.1(10)	2951.2(9)	38.3(4)
N1	-3533.9(14)	7589.8(7)	-99.4(6)	26.7(3)
N2	-5274.5(14)	6656.3(8)	200.6(6)	24.7(2)
N3	-4722.2(14)	5321.8(7)	594.8(6)	24.3(2)
N4	-1305.3(14)	6247.6(7)	240.9(6)	25.4(2)
N5	-2132.6(13)	5056.3(7)	610.9(6)	21.5(2)
N6	4121.1(13)	4358.5(7)	1665.9(6)	22.8(2)
O1	-905.1(11)	4003.8(6)	1134.1(5)	22.2(2)
O2	-2988.8(11)	2882.4(6)	427.9(5)	27.5(2)
O3	1986.8(10)	3334.6(6)	1438.9(5)	23.3(2)
O4	4476.4(11)	3123.5(6)	955.2(5)	26.6(2)
O5	3847.6(11)	2924.2(6)	2135.5(5)	26.2(2)
O6	1781.0(12)	5396.8(7)	1377.5(5)	28.7(2)
O7	1031.8(13)	5281.0(7)	2377.3(5)	32.4(2)
P1	3681.3(3)	3428.2(2)	1504.61(16)	19.98(9)
C27	8789(3)	1042.6(12)	674.7(12)	52.0(5)
C28	7891(2)	284(1)	712.4(8)	34.5(3)
C29	7754(2)	-1038.7(11)	1127.4(9)	37.3(4)
C30	8758(2)	-1623.0(12)	1450.5(11)	50.1(5)
O8	6769.5(15)	176.2(9)	441.8(7)	43.5(3)
O9	8516.8(15)	-277.8(8)	1073.6(6)	39.7(3)
H1	-6584	5821	480	32

H5	-10	5334	467	30
H6	-3022	4122	1098	24
H7	-2795	3874	-110	27
H8A	-271	3856	-113	28
H8B	-630	2917	-100	28
H9	-372	2839	970	25
H10A	1933	3167	504	27
H10B	1664	4073	701	27
H12	6505	2765	1680	40
H13	8599	2476	2245	46
H14	8507	2310	3318	45
H15	6332	2428	3838	46
H16	4223	2728	3287	39
H17	3160	4455	2504	32
H18A	5248	5264	2560	63
H18B	3887	5792	2720	63
H18C	4607	5856	2056	63
H20A	-748	5342	1834	39
H20B	-265	6193	2096	39
H22	-1731	6706	2913	37
H23	-3319	6526	3748	40
H24	-3868	5233	4097	42
H25	-2863	4117	3596	51
H26	-1349	4293	2746	46
H1A	-4213	7928	-186	32
H1B	-2644	7726	-154	32
H6A	5043	4398	1709	27
H2	-3809	2975	556	41
H27A	9233	1077	268	78
H27B	9526	1028	992	78
H27C	8181	1507	740	78
H29A	7494	-1241	715	45
H29B	6876	-969	1371	45
H30A	9663	-1642	1230	75
H30B	8331	-2154	1454	75
H30C	8922	-1447	1874	75

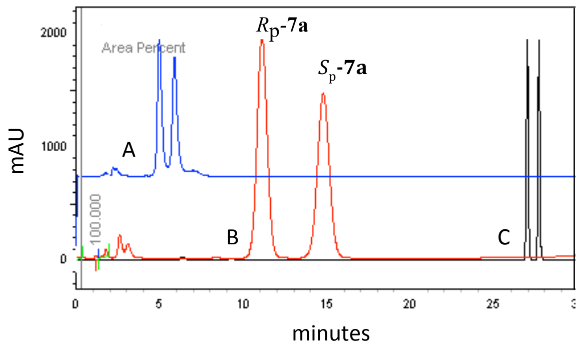

Figure S1. Analytical HPLC traces of **7a** diastereomeric mixture. HPLC isocratic conditions: (A) 60:40 CH_3OH:H_2O, 30 min (blue). (B) 55:45 CH_3OH:H_2O, 30 min (red). (C) 40:60 CH_3OH:H_2O, 30 min (black). Flow = 1 mL min; λ= 254 nm.

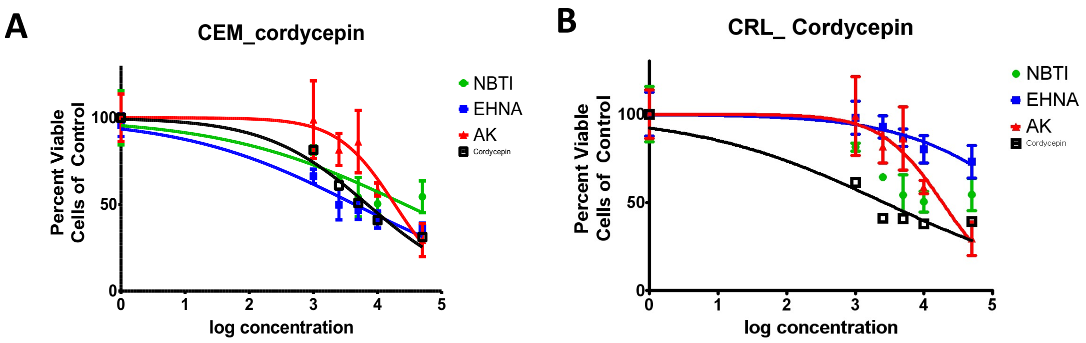

Figure S2. *In vitro* cytoxicity of 3’-dA in the presence/absence of hENT1, AK and ADA inhibitors in CEM (**A**) and CRL (**B**) cell lines.

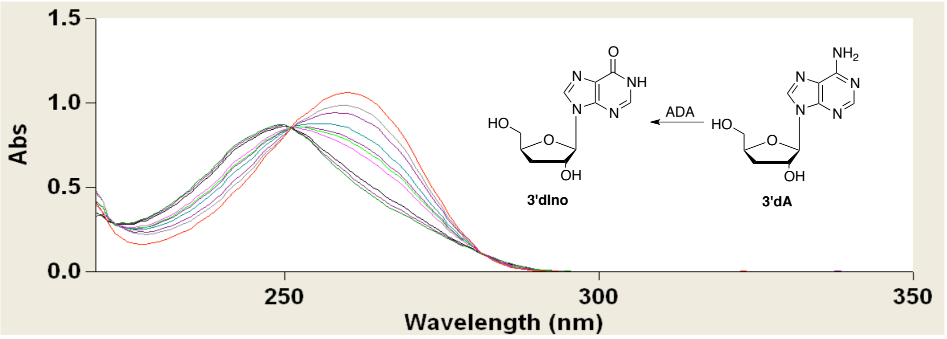

Figure S3. Change in UV absorbance spectra of 3’-dA after addition of a solution of ADA (in phosphate buffer pH 7.4).

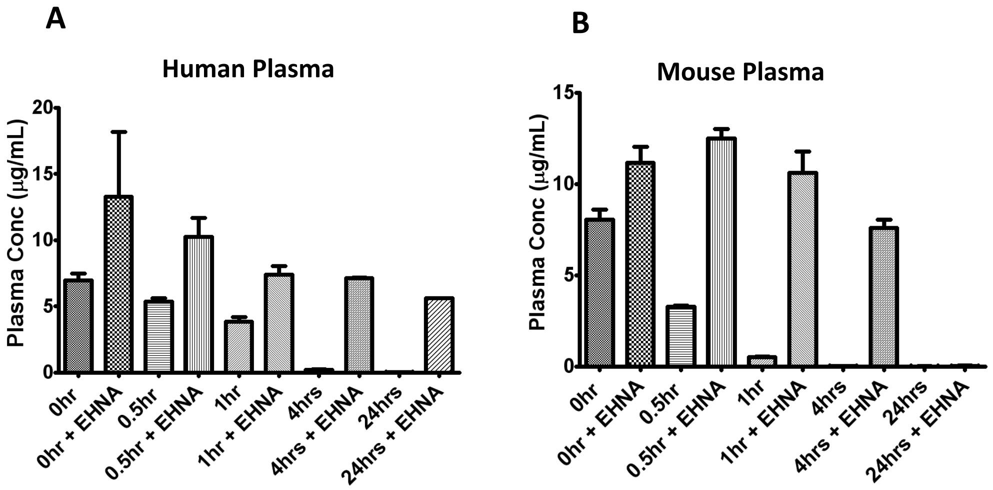

Figure S4. Stability assay of 3'-dA in human plasma (A) and mouse plasma (B).

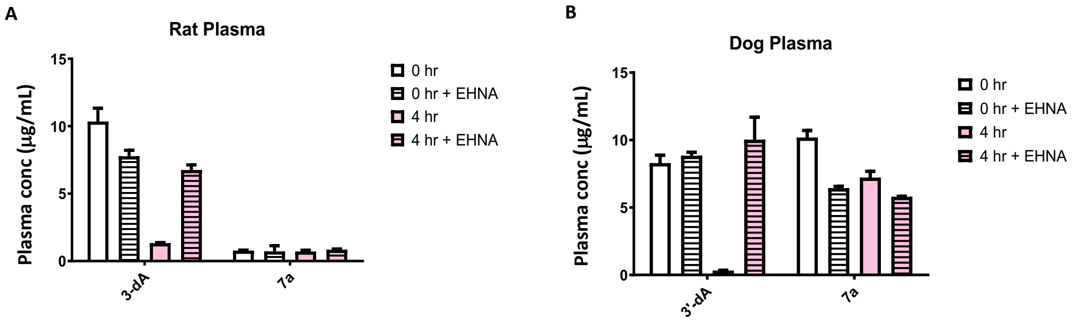

Figure S5. Stability assay of 3'dA and **7a** in rat plasma (A) and dog plasma (B).

9-(2',3'-Anhydro-α-D-ribofuranosyl) adenine (3)

To a stirring suspension of adenosine (**2**) (10.00 g, 37.42 mmol) in CH_3CN (250 mL), α-AIBBr (22.03 mL, 149.68 mmol, 4 eq) and H_2O (0.67 mL, 0.037 mmol, 0.001 eq) were added, and stirring was continued at room temperature. After 1 hour, the mixture was neutralised by addition of a saturated solution of $NaHCO_3$ (a change in colour from dark orange to clear-white could be noticed) and the solution was extracted with EtOAc (2 x 5 mL per mmol of adenosine analogue). The combined organic phase was washed with brine (1 mL x mmol of adenosine analogue). The aqueous phase was extracted with EtOAc (1 x 190 mL) and the combined organic phase was dried over Na_2SO_4, filtered and evaporated to give a white gum. The crude mixture was dissolved in CH_3OH (260 mL) and stirred for 16 hours with Amberlite (2 x OH) resin (150 mL, 1.1 meq/mL by wetted bed volume), previously washed with CH_3OH). The solution was then filtered, and the resin carefully washed with CH_3OH until no spot of the product by TLC could be detected in the filtrate. Evaporation of the combined filtrate and crystallisation of the residue from EtOH gave **3**

as a white powder (8.86 g, 95%). Melting point 178-180 °C (Lit. mp: 180-181 °C). ^{1}H NMR (500 MHz, DMSO-*d6*) δ_H 8.33 (s, 1H, H-2), 8.18 (s, 1H, H-8), 7.29 (br s, 2H, NH_2), 6.21 (s, 1H, H-1'), 5.05 (br s, 1H, OH-5'), 4.46 (d, *J* = 2.6 Hz, 1H, H-2'), 4.22 (d, *J* = 2.6 Hz, 1H, H-3'), 4.18 (t, *J* = 5.2 Hz, 1H, H-4'), 3.60-3.55 (m, 1H, H-5'), 3.53-3.49 (m, 1H, H-5'). ^{13}C NMR (125 MHz, DMSO-*d6*) δ_C 156.01 (C-6), 152.61 (C-2), 149.11 (C-4), 139.55 (C-8), 119.52 (C-5), 91.25 (C-1'), 81.15 (C-4'), 75.06 (C-2'), 58.75 (C-3'), 57.70 (C-5'). HPLC Reverse-phase HPLC eluting with H_2O/CH_3CN from 100/0 to 75/25 in 30 minutes, F = 1ml/min, λ = 254 nm, showed one peak with t_R 13.20 min. $C_{10}H_{13}N_5O_3$ required *m/z* 249.23 [M]; (ES+) found *m/z* 272.09 $[M+Na]^+$, 250.09 $[M+H]^+$

3'-Deoxyadenosine (1) 2',3'-anhydrous adenosine **3** (9.12 g, 36.59 mmol, 1 eq) was dissolved in a mixture of DMSO (55 mL) and THF (550 mL), under Argon atmosphere. The solution was cooled down to 0 ºC and 1M $LiEt_3BH$ in THF (110 mL, 109.77 mmol, 3 eq) was added dropwise. Stirring was continued at ~4 °C for 1 h and at rt for 16 h. The mixture was cooled down to 0 °C and an additional portion of 1M $LiEt_3BH$ in THF (36.6 mL, 36.6 mmol, 1 eq) was added dropwise. The mixture was stirred at 0 ºC for 1 hour and then at rt for one additional hour. The reaction mixture was carefully acidified (5% $AcOH/H_2O$), purged with N_2 for 1 h (under the fume hood) to remove *pyrophoric* triethylborane, and evaporated. Purification by silica gel flash column chromatography (eluent system CH_3OH/CH_2Cl_2 5/95 to 20/80) afforded the title compound **1** as a white solid (9.01 g, 98%). Melting point: 188-190 °C (Lit. mp: 191-192 °C).^{1}H-NMR (500 MHz, DMSO-*d6*) δ_H 8.37 (s, 1H, H-8), 8.17 (s, 1H, H-2), 7.29 (br s, 2H, NH_2), 5.89 (d, *J* = 2.5 Hz, 1H, H-1'), 5.68 (d, *J* = 4.5 Hz, 1H, OH-2'), 5.19 (t, *J* = 6.0 Hz, 1H, OH-5'), 4.63-4.58 (m, 1H, H-2'), 4.40-4.34 (m, 1H, H-4'), 3.71 (ddd, *J* = 12.0, 6.0, 3.0 Hz, 1H, H-5'), 3.53-3.49 (ddd, *J* = 12.0, 6.0, 4.0 Hz, 1H, H-5'). ^{13}C NMR (125 MHz, DMSO-*d6*) δC 156.00 (C-6), 152.41 (C-2), 148.82 (C-4), 139.09 (C-8), 119.06 (C-5), 90.79 (C-1'), 80.66 (C-4'), 74.56 (C-2'), 62.61 (C-5'), 34.02 (C-3'). $C_{10}H_{13}N_5O_3$ required *m/z* 251.24 [M]. (ES+) found *m/z* 258.12 $[M+Li]^+$, 274.09 $[M+Na]^+$, 252.11 $[M+H]^+$. HPLC Reverse-phase eluting with H_2O/CH_3CN from 100/0 to 75/25 in 30 minutes, F = 1ml/min, λ = 254 nm, t_R 11.22 min.

Ethyl 3-(2-hydroxyphenyl)propanoate.[1] 2,3-Dichloro-5,6-dicyano-1,4-benzoquinone (771 mg, 3.4 mmol) was added to a solution of 3,4-dihydrocoumarin (7.63 g, 51.5 mmol) in EtOH (40 mL) and the resulting orange solution was stirred at room temperature for 8 hours. The crude was purified by flash column chromatography (eluent: CH_2Cl_2) to afford **4** as a yellow solid (9.6 g,

96%). Lit. mp = 36-36.5 °C.^{1}H NMR (500 MHz, $CDCl_3$) δ_H 7.29-7.20 (m, 1H, Ar), 7.19-7.16 (m, 1H, Ar), 7.10-7.05 (m, 2H, Ar), 4.12 (q, *J* = 7.2 Hz, 2H, $CO_2CH_2CH_3$), 3.00 (t, *J* = 7.7 Hz, 2H, $CH_2CH_2CO_2$), 2.61 (t, *J* = 7.7 Hz, 2H, $CH_2CO_2CH_2CH_3$), 1.22 (t, *J* = 7.2 Hz, 3H, $CO_2CH_2CH_3$).[1]

Naphth-1-yl dichlorophosphate. Phosphorus oxychloride (3.9 mL, 41.8mmol), (1 eq) 1-naphthol (6.06 g, 42.03 mmol) were stirred in Et_2O (45 mL). The solution was cooled to -78 °C and Et_3N (5.9 mL, 42.3 mmol) was added. After 30 minutes the solution was allowed to reach rt and stirred for 1.5 hours. The triethylamine hydrochloride salt was filtered off through a sintered funnel and the solvent removed under vacuum to afford the crude product as a clear oil. The product was obtained as a clear oil (9.98 g, 91%).^{31}P NMR (202 MHz, CDCl3) δ_P 3.69. ^{1}H NMR (500 MHz, $CDCl_3$) δ_H 8.14 (d, *J* = 8.3 Hz, 1H, H-8), 7.90 (d, *J* = 8.1 Hz, 1H, H-5), 7.81 (d, *J* = 8.3 Hz, 1H, H-4), 7.68-7.54 (m, 3H, H-2, H-6, H-7), 7.46 (d, *J* = 8.0 Hz, 1H, H-3).

Ethyl 3-(2-hydroxyphenyl)propanoyl dichlorophosphate. Phosphorus oxychloride (2.48 mL g, 26.62 mmol) and ethyl 3-(2- hydroxyphenyl)propanoate (5.17 g, 26.62 mmol) were stirred in Et_2O (27 mL). The solution was cooled to -78 °C and Et_3N (3.71 mL, 26.62 mmol) was added. After 30 minutes the solution was allowed to reach rt and stirred for 1.5 hours. The triethylamine hydrochloride salt was filtered off through a sintered funnel and the solvent removed under vacuum to afford the crude product as a clear oil.The product was obtained as a clear oil (7.45 g, 90%). ^{31}P NMR (202 MHz, $CDCl_3$) δ_P 3.22. ^{1}H NMR (500 MHz, $CDCl_3$) δ_H 7.43-7.40 (m, 1H, Ar), 7.36-7.33 (m, 1H, Ar), 7.31-7.23 (m, 2H, Ar), 4.15 (q, J = 7.2 Hz, 2H, $CH_2CH_2CO_2CH_2CH_3$), 3.06 (t, *J* = 7.7 Hz, 2H).

[1] Gutsche CD, Oude-Alink BAM. The photoinduced alcoholysis of 3,4-dihydrocoumarin and related compounds. J. *Am. Chem. Soc.* **1968**;90(21):5855-61

Standard procedure A for the synthesis of aryl amino acid ester phosphorochloridates from amino acid hydrochloride or *p*-toluene sulfonate salts (4a-f).

The appropriate amino acid ester salt (1 eq) was dissolved in CH_2Cl_2 (4 mL per mmol of amino acid ester) under Argon atmosphere. A solution of the appropriate phosphorodichloridate (1 eq) in CH_2Cl_2 (1 mL per mmol of amino acid ester) was added and the mixture cooled to -78 °C. Et_3N (2 eq) was added dropwise and the reaction mixture was stirred at -78 °C for 20 minutes and thereafter at rt for 2.5 hours. The solvent was evaporated under reduced pressure and the resulting oil was triturated with anhydrous Et_2O and the filtrate reduced to give the crude product as an oil.

Phenyl-(benzoxy-L-alaninyl)dichlorophosphate (4a). Prepared according to the general procedure **A** using L-alanine benzyl ester hydrochloride salt (1.12 g, 5.20 mmol), phenyl dichlorophosphate (1.10 g, 5.20 mmol) and Et_3N (1.45 ml, 10.40 mmol) in CH_2Cl_2 (20 ml). The product was obtained as a clear oil (1.51 g, 82%).^{31}P NMR (202 MHz, $CDCl_3$) δ_P 7.51, 7.85. ^{1}H NMR (500 MHz, $CDCl_3$) δ_H 7.36-7.25 (m, 10 H, Ar), 5.15 (s, 1H, C*H₂*Ph), 5.11 (s,1H, C*H₂*Ph), 4.25-4.14 (m, 2H, NH, C*H*CH_3), 1.59-1.57 (m, 3H, CHC*H₃*).

Naphth-1-yl-(benzoxy-L-alaninyl) dichlorophosphate (4b). Prepared according to the general procedure **A** using L-alanine benzyl ester hydrochloride salt (430 mg, 1.99 mmol), naphth-1-yl dichlorophosphate (0.52 g, 1.99 mmol) and Et_3N (0.55 mL, 3.98 mmol) in CH_2Cl_2 (8 ml). The product was obtained as a clear oil (87%, 0.70 g). ^{31}P NMR (202 MHz, $CDCl_3$) δ_P 8.3, 8.0. ^{1}H NMR (500 MHz, $CDCl_3$) δ_H 8.10-7.30 (m, 12H, Ar), 5.12 (s, 1H, C*H₂*Ph), 5.19 (s, 1H, C*H₂*Ph), 4.70-4.64 (m, 1H, NH), 4.42-4.33 (m, 1H, C*H*CH_3), 1.59-1.57 (m, 3H, CHC*H₃*).

Phenyl-(benzoxy-glycinyl)dichlorophosphate (4c). Prepared according to the general procedure **A** using glycine benzylester hydrochloride salt (1.78 g, 8.83 mmol), phenyl dichlorophosphate (1.32 mL, 8.83 mmol) and Et_3N (2.50 mL, 17.66 mmol) in CH_2Cl_2 (35 mL). The product was obtained as a clear oil (2.40 g, 80%). ^{31}P NMR (202 MHz, $CDCl_3$) δ_P 9.04. ^{1}H NMR (500 MHz, $CDCl_3$) δ_H 7.42-7.34 (m, 7H, Ar), 7.31-7.22 (m, 3H, Ar), 5.14-5.07 (m, 1H, C*H*$(CH_3)_2$), 4.62-4.48 (m, 1H, NH), 4.01-3.94 (m, 2H, CH_2).

Naphth-1-yl-(pent-1-oxy-L-leucinyl)dichlorophosphate (4d). Prepared according to the general procedure **A** using L-leucine pent-1-yl ester hydrochloride salt (2.00 g, 8.41 mmol), naphth-1-yl dichlorophosphate (2.19 g, 8.41 mmol) and Et_3N (2.34 mL, 16.82 mmol) in CH_2Cl_2 (35 mL). The product was obtained as a clear oil (3.19 g, 89%).^{31}P NMR (202 MHz, $CDCl_3$) δ_P 8.78, 8.52.^{1}H NMR (500 MHz, $CDCl_3$) δ_H 8.12 (d, J = 8.0 Hz, 1H, Ar), 7.91-7.87 (m, 1H, Ar), 7.77, 7.73 (m,

1H, Ar), 7.65-7.54 (m, 3H, Ar) 7.47-7.42 (m, 1H, Ar), 4.48-4.36 (m, 1H, NH), 4.28-4.21 (m, 1H, C*H*NH), 4.21-4.12 (m, 2H, OCH_2), 1.91-1.76 (m, 2H, CH_2CH(CH_3)$_2$), 1.76-1.60 (m, 3H, C*H*(CH3)$_2$ L-Leu, OCH$_2$CH_2), 1.41-1.30 (m, 4H, CH_2CH_2CH$_3$), 1.01-0.94 (m, 6H, CH(CH_3)$_2$ L-Leu), 0.94-0.86 (m, 3H, CH$_2$CH_3).

Ethyl 3-(2-hydroxyphenyl)propanoyl-(benzyloxy-L-alaninyl)dichlorophosphate (4e). Prepared according to the general procedure **A** using L-alanine-*O*-benzyl ester hydrochloric salt (1.70 g, 7.88 mmol), ethyl 3-(2-hydroxyphenyl)propanoyl dichlorophosphate (2.45 g, 7.88 mmol) and Et$_3$N (2.20 mL, 15.76 mmol) in CH_2Cl_2 (35 mL). The product was obtained as a clear oil (3.29 g, 92%). ^{31}P NMR (202 MHz, $CDCl_3$) δ_P 7.53, 7.70. ^{1}H NMR (500 MHz, $CDCl_3$) δ_H 7.50-7.44 (m, 1H, Ar), 7.41-7.32 (m, 5H, Ar), 7.28-7.14 (m, 3H, Ar), 5.26-5.17 (m, 2H, CH_2Ph), 4.67-4.56 (m, 1H, NH), 4.34-4.22 (m, 1H, C*H*CH$_3$), 4.15-4.09 (m, 2H, OCH_2CH$_3$), 3.15-2.93 (m, 2H, CH$_2$CH_2CO$_2$), 2.66-2.59 (m, 2H, CH_2CH$_2$CO$_2$), 1.58-1.52 (m, 3H, CHCH_3), 1.24-1.20 (m, 3H, OCH$_2$CH_3).

Naphth-1-yl-(methoxy-dimethylglycinyl)dichlorophosphate (4f). Prepared according to the general procedure **A** using dimethyl glycine methyl ester hydrochloride salt (1.0 g, 6.50 mmol), naphth-1-yl dichlorophosphate (1.70 g, 6.50 mmol) and Et$_3$N (1.81 mL, 13.00 mmol) in CH_2Cl_2 (26 mL). The product was obtained as a clear oil (1.66 g, 75%).^{31}P NMR (202 MHz, $CDCl_3$) δ_P 6.02. ^{1}HNMR(500MHz,$CDCl_3$)δ_H 8.15(d, *J*=8.1Hz, 1H, Ar),7.87(d, *J*=8.1Hz,1H, Ar), 7.73 (d, *J* = 8.1 Hz, 1H, Ar), 7.64-7.61 (m, 1H, Ar), 7.60-7.52 (m, 2H, Ar), 7.44 (t, *J* = 7.7 Hz, 1H, Ar), 4.97 (br s, 1H, NH), 3.81 (s, 3H, OCH$_3$), 1.77 (s, 3H, CH$_3$), 1.73 (s, 3H, CH$_3$).

Standard procedure B for the synthesis of ProTides 5a-b and 6a-b

3'-deoxyadenosine (**1**) (1 eq) was dissolved in THF (20 ml per 0.1 mmol of nucleoside) under Argon atmosphere. 1M tBuMgCl in THF (1.1 eq) was added dropwise. The appropriate phosphorochloridate (**4a-b**) (3 eq) was dissolved in THF (4 mL per 1 mmol of phosphorochloridate) and the resulting solution added to the initial mixture. The mixture was stirred for 12-16 hours, and the solvent evaporated under vacuum. The obtained crude was purified by silica gel flash column chromatography. In some cases, further purification by preparative TLC was necessary.

3'-deoxyadenosine-2',5'-O-[phenyl(benzoxy-L-alaninyl)] diphosphate (5a) and 3'-deoxyadenosine-2'-O-[phenyl(benzoxy-L-alaninyl)] phosphate (6a). Prepared according to the general procedure **B** using 3'-deoxyadenosine (50 mg, 0.20 mmol), 1.0 M ^{t}BuMgCl in THF (0.22 mL, 0.22 mmol) and phenyl(benzoxy-L-alaninyl) phosphorochloridate **5a** (212 mg, 0.6 mmol). Purification by column chromatography (eluent system CH_3OH/CH_2Cl_2 0/100 to 5/95) followed by preparative TLC (1000 μM, eluent system CH_3OH/CH_2Cl_2 = 5/95) afforded **5a** as a white solid (19 mg, yield = 11%). ^{31}P NMR (202 MHz, CD_3OD): δ_P 3.98, 3.88, 3.59, 3.12, 3.05, 2.45, 2.32. ^{1}H NMR (500 MHz, CD_3OD): δ_H 8.24-8.13 (m, 2H, H-8, H-2), 7.39-7.08 (m, 20H, Ph), 6.27-6.23 (m, 0.5H, H-1'), 6.16-6.13 (m, 0.5H, H-1'), 5.61-5.48 (m, 1H, H-2'), 5.17-4.91 (m, 4H, OC*H*$_2$Ph), 4.57-4.49 (m, 1H, H-4'), 4.41-4.29 (m, 1H, H-5'), 4.25-4.15 (m, 1H, H-5'), 4.10-4.01 (m, 1H, C*H*CH$_3$), 3.99-3.89 (m, 1H, C*H*CH$_3$), 2.57-2.41 (m, 1H, H-3'), 2.28-2.17 (m, 1H, H-3'), 1.38-1.23 (m, 6H, CHC*H$_3$*). ^{13}C NMR (125 MHz, CD_3OD): δ_C 174.88 (C=O), 174.83 (C=O), 174.79 (C=O), 174.73 (C=O), 174.61 (C=O), 174.57 (C=O), 174.53 (C=O), 157.36 (C-6), 157.34 (C-6), 157.32(C-6), 157.29 (C-6), 154.04 (C-2), 154.01 (C-2), 153.97 (C-2), 153.94 (C-2), 152.09 (C-4), 152.04 (C-4), 152.02 (C-4), 151.97 (C-4), 150.31 (C-Ar), 150.29 (C-Ar), 150.16 (C-Ar), 140.98 (C8), 140.91 (C8), 140.81 (C8), 137.31 (C-Ar), 137.28 (C-Ar), 137.22 (C-Ar), 137.09 (C-Ar), 130.86 (CH-Ar), 130.78 (CH-Ar), 130.77 (CH-Ar), 129.65 (CH-Ar), 129.61 (CH-Ar), 129.58 (CH-Ar), 129.55 (CH-Ar), 129.44 (CH-Ar), 129.42 (CH-Ar), 129.38 (CH-Ar), 129.34 (CH-Ar), 129.32 (CH-Ar), 129.30 (CH-Ar), 129.28 (CH-Ar), 129.23 (CH-Ar), 129.21 (CH-Ar), 12.42 (CH-Ar), 126.23 (CH-Ar), 126.20 (CH-Ar), 126.17 (CH-Ar), 121.65 (CH-Ar), 121.63 (CH-Ar), 121.61 (CH-Ar), 121.59 (CH-Ar), 121.52 (CH-Ar), 121.50 (CH-Ar), 121.47 (CH-Ar), 121.46 (CH-Ar), 121.40 (CH-Ar), 121.39 (CH-Ar), 121.36 (CH-Ar), 121.35 (CH-Ar), 121.30 (CH-Ar), 121.28 (CH-Ar), 121.26 (CH-Ar), 121.24 (CH-Ar), 120.61 (C-5), 120.57 (C-5), 120.56 (C-5), 120.54 (C-5), 91.56 (C-1'), 91.51 (C-1'), 91.45 (C-1'), 91.25 (C-1'), 91.20 (C-1'), 81.84 (C-2'), 81.82 (C-2'), 81.79 (C-2'), 81.27 (C-2'), 81.22 (C-2'), 81.18 (C-2'), 80.49 (C-4'), 80.43 (C-4'), 80.06 (C-4'), 79.99 (C-4'), 68.29 (C-5', OCH$_2$Ph), 68.25 (C5', O*C*H$_2$Ph), 68.00 (C-5', O*C*H$_2$Ph), 67.96 (C-5', O*C*H$_2$Ph), 67.94 (C-5', O*C*H$_2$Ph), 67.90 (C-5', O*C*H$_2$Ph), 67.71 (C-5', O*C*H$_2$Ph), 67.67 (C-5', O*C*H$_2$Ph), 51.91 (*C*HCH$_3$), 51.74 (*C*HCH$_3$), 51.70 (*C*HCH$_3$), 51.59 (*C*HCH$_3$), 34.22 (C-3'), 34.20 (C-3'), 34.16 (C-3'), 33.97 (C-3'), 33.94 (C-3'), 33.91 (C-3'), 20.44 (CH*C*H$_3$), 20.43 (CH*C*H$_3$), 20.39 (CH*C*H$_3$), 20.29 (CH*C*H$_3$), 20.27 (CH*C*H$_3$), 20.24 (CH*C*H$_3$), 20.21 (CH*C*H$_3$), 20.19 (CH*C*H$_3$). $C_{42}H_{45}N_7O_{11}P_2$ required *m/z* 885.3 [M]. (ES+) found *m/z* 886.3 $[M + H]^+$, 1771.6 $[2M + H]^+$. Reverse-phase HPLC eluting with H_2O/CH_3CN from 90/10 to 0/100 in 30 minutes, F=1ml/min, λ = 280 nm, t_R 19.35 min.

Further elution by column chromatography with CH_3OH/CH_2Cl_2 8/92 followed by preparative TLC (500 µM, eluent system CH_3OH/CH_2Cl_2 = 5/95) afforded **6a** as a white solid (6 mg, 5%). ^{31}P NMR (202 MHz, CD_3OD) δ_P 2.44, 2.92. ^1H NMR (500 MHz, CD_3OD): δ_H 8.41 (s, 0.5 H, H-8), 8.28 (s, 0.5 H, H-8), 8.19 (s, 0.5H, H-2), 8.18 (s, 0.5H, H-2), 7.39-7.30 (m, 4H, Ar), 7.28-7.18 (m, 4H, Ar), 7.17-7.11 (m, 1H, Ar), 7.08-7.03 (m, 1H, Ar), 6.23 (d, J = 2.0 Hz, 0.5H, H-1'), 6.08 (d, J = 3.4 Hz, 0.5H, H-1'), 5.52-5.43 (m, 1H, C-2'), 5.19-5.12 (m, 1H, OC*H*$_2$Ph), 5.07-4.95 (m, 1H, OC*H*$_2$Ph), 4.48-4.42 (m, 1H, H-4'), 4.05-3.97 (m, 1H, C*H*CH_3), 3.95-3.87 (m, 1H, H-5'), 3.69-3.61 (m, 1H, H-5'), 2.59-2.45 (m, 1H, H-3'), 2.31-2.23 (m, 1H, H-3'), 1.36-1.27 (m, 3H, CHC*H*$_3$). ^{13}C NMR (125 MHz, CD_3OD): δ_C 174.76 (d, $^3J_{C-P}$ = 5.0 Hz, C=O), 174.52 (d, $^3J_{C-P}$ = 5.0 Hz, C=O), 157.44 (C-6), 153.76 (C-2), 151.93 (C-4), 150.06 (C-Ar), 149.93 (C-Ar), 141.38 (C-8), 141.18 (C-8), 137.33 (C-Ar), 137.10 (C-Ar), 130.69 (CH-Ar), 130.79 (CH-Ar), 129.61 (CH-Ar), 129.51 (CH-Ar), 129.40 (CH-Ar), 129.30 (CH-Ar), 129.23 (CH-Ar), 126.33 (CH-Ar), 126.16 (CH-Ar), 121.53 (d, $^3J_{C-P}$ = 4.5 Hz, CH-Ar), 121.20 (d, $^3J_{C-P}$ = 4.5 H, CH-Ar), 120.76 (C-5), 91.56 (d, $^3J_{C-P}$ = 7.7 Hz, C-1'), 91.45 (d, $^3J_{C-P}$ = 7.7 Hz, C-1'), 82.78 (C-4'), 82.28 (C-4'), 81.83 (d, $^2J_{C-P}$ = 4.7 Hz, C-2'), 80.96 (d, $^2J_{C-P}$ = 4.7 Hz, C-2'), 67.95 (O*C*H_2Ph), 67.92 (O*C*H_2Ph), 64.13 (C-5'), 63.59 (C-5'), 51.88 (*C*HCH_3), 51.75 (*C*HCH_3), 33.75 (C-3'), 33.59 (C-3'), 20.33 (d, $^3J_{C-P}$ = 7.1 Hz , CH*C*H_3), 20.18 (d, $^3J_{C-P}$ = 7.1 Hz, CH*C*H_3). $C_{26}H_{29}N_6O_7P$ required *m/z* 568.2 [M]. (ES+) Found *m/z:* 569.2 $[M + H]^+$, 591.2 $[M + Na]^+$, 1159.4 $[2M + Na]^+$. Reverse-phase HPLC eluting with H_2O/CH_3OH from 90/10 to 0/100 in 30 min., F= 1ml/min, λ = 254 nm, t_R 16.31 min.

3'-Deoxyadenosine 2',5'-bis-O-naphth-1-yl-(benzoxy-L-alaninyl)-phosphate (5b) and 3'-deoxyadenosine-2'-*O*-[1-naphthyl(benzoxy-L-alaninyl)] phosphate (6b). Prepared according to the general procedure **B** using 3'-deoxyadenosine (50 mg, 0.20 mmol), ^{t}BuMgCl (1.0 M solution in THF, 0.22 mL, 0.22 mmol) and naphthyl(benzoxy-L-alaninyl) phosphorochloridate **4b** (323 mg, 0.8 mmol). Purification by column chromatography (eluent system CH_3OH/CH_2Cl_2 0/100 to 5/95) followed by preparative TLC (eluent system CH_3OH/CH_2Cl_2 0/100 to 6/94) afforded **5b** as a white solid (0.069 g, 35%). ^{31}P NMR (202 MHz, CD_3OD) δ_P 4.23, 4.18, 3.97, 3.94, 3.48, 3.39, 2.78, 2.77. ^1H NMR (500 MHz, CD_3OD) δH 8.18-8.05 (m, 3H, H-8, H-2, Ar), 8.03-7.81 (m, 3H, Ar), 7.74-7.62 (m, 2H, Ar), 7.54-7.39 (m, 7H, Ar), 7.37-7.16 (m, 11H, Ar), 6.22-6.18 (m, 0.5H, H-1'), 6.05-6.02 (m, 0.5H, H-1'), 5.67-5.54 (m, 1H, H-2'), 5.15-4.96 (m, 4H, 2 x C*H*$_2$Ph), 4.50-4.42 (m, 1H, H-4'), 4.39-4.31 (m, 1H, H-5'), 4.24-4.16 (m, 1H, H-5'), 4.15-4.08 (m, 1H, C*H*CH_3), 4.04-3.91 (m, 1H, C*H*CH_3), 3.60-2.41 (m, 1H, H-3'), 2.29-2.09 (m, 1H, H-3'), 1.37-1.27 (m, 6H,

CHCH_3 - x 2).13C MISSING description Reverse-phase HPLC eluting with H_2O/CH_3OH from 90/10 to 0/100 in 30 minutes, F=1ml/min, λ = 280 nm, t_R 19.64 min.

Further elution by column chromatography with CH_3OH/CH_2Cl_2 8/92 followed by preparative TLC (1000 μM, eluent system CH_3OH/CH_2Cl_2 = 5/95) afforded **6b** as a white solid (14 mg, 11%). ^{31}P NMR (202 MHz, CD_3OD): δ_P 3.27, 2.75.^{1}H NMR (500 MHz, CD_3OD): δ_H 8.37 (s, 1H, H8), 8.18 (s, 1H, H8), 8.14 (s, 1H, H-2), 8.13-8.11 (m, 0.5 H, Nap) 8.11 (s, 1H, H-2), 7.94-7.90 (m, 0.5 H, Ar), 7.90-7.87 (m, 0.5 H, Ar), 7.86-7.82 (m, 0.5 H, Ar), 7.74-7.70 (m, 0.5 H, Ar), 7.66-7.61 (m, 0.5 H, Ar), 7.57-7.47 (m, 1.5 H, Ar), 7.46-7.37 (m, 2.5 H, Ar), 7.34-7.27 (m, 4 H, Ar), 7.25-7.17 (m, 1 H, Ar), 6.19 (d, J = 2.4 Hz, 0.5H, H-1'), 6.04 (d, J = 2.4 Hz, 0.5H, H-1'), 5.60-5.54 (m, 0.5H, H-2'), 5.50-5.42 (m, 0.5H, H-2'), 5.16-4.99 (m, 2H, OCH_2Ph), 4.46-4.40 (m, 0.5H, H-4'), 4.36-4.30 (m, 0.5H, H-4'), 4.13-4.04 (m, 1H, CHCH$_3$), 3.90-3.83 (m, 1H, H-5'), 3.64-3.56 (m, 1H, H-5'), 2.61-2.54 (m, 0.5H, H-3'), 2.49-2.41 (m, 0.5H, H-3'), 2.35-2.27 (m, 0.5H, H-3'), 2.22-2.16 (m, 0.5H, H-3'), 1.35-1.24 (m, 3H, CHCH_3). ^{13}C NMR (125 MHz, CD_3OD): δ_C 174.52 (C=O), 174.49 (C=O), 157.27 (C-6), 153.58 (C-2), 149.97 (C-4), 149.93 (C-4), 147.70 (d, $^3J_{C-P}$ = 7.5, 'ipso' Nap), 147.48 (d, $^3J_{C-P}$ = 7.5, 'ipso' Nap), 141.36 (C-8), 141.19 (C-8), 137.25 (C-Ar), 137.05 (C-Ar), 136.31 (C-Ar), 136.20 (C-Ar), 129.58 (CH-Ar), 129.48 (CH-Ar), 129.37 (CH-Ar), 129.26 (CH-Ar), 129.22 (CH-Ar), 128.88 (CH-Ar), 127.84 (CH-Ar), 127.75 (CH-Ar), 127.49 (CH-Ar), 127.44 (CH-Ar), 126.48 (CH-Ar), 126.39 (CH-Ar), 126.26 (CH-Ar), 126.05 (CH-Ar), 122.76 (CH-Ar), 122.38 (CH-Ar), 120.68 (C-5), 120.61 (C-5), 116.64 (d, $^3J_{C-P}$ = 3.75 Hz, CH-Ar), 116.13 (d, $^3J_{C-P}$ = 3.75, CH-Ar), 91.60 (d, $^3J_{C-P}$ = 7.5 Hz, C1'), 91.43 (d, $^3J_{C-P}$ = 7.5 Hz, C-1'), 82.74 (C-4'), 82.27 (C-4'), 81.99 (d, $^2J_{C-P}$ = 5.5 Hz, C-2'), 81.12 (d, $^2J_{C-P}$ = 5.5 Hz, C-2'), 67.97 (OCH$_2$Ph), 67.94 (OCH$_2$Ph), 64.16 (C-5'), 63.51 (C-5'), 51.96 (CHCH$_3$), 51.89 (CHCH$_3$), 33.89 (C-3'), 33.63 (C-3'), 20.28 (d, $^3J_{C-P}$ = 7.5 Hz, CHCH$_3$), 20.23 (d, $^3J_{C-P}$ = 7.1 Hz , CHCH$_3$). $C_{30}H_{31}N_6O_7P$ required *m/z* 618.20 [M]. (ES+) found *m/z* 619.2 $[M + H]^+$, 641.2 $[M + Na]^+$, 1259.4 $[2M + Na^+]$. Reverse-phase HPLC eluting with H_2O/CH_3OH from 90/10 to 0/100 in 30 minutes, F=1ml/min, λ = 280 nm, t_R 14.80 and 15.01min.

Standard procedure C for the synthesis of ProTides 7a-f

3'-deoxyadenosine (**1**) (1 eq) was dissolved in THF (7 ml per 0.1 mmol of nucleoside) under Argon atmosphere. The appropriate phosphorochloridate (**4a-f**) (3 eq) was dissolved in THF (4 mL per 1 mmol of phosphorochloridate) and the resulting solution added to the initial mixture, followed by

NMI (5 eq). The mixture was stirred for 12-16 hours, and the solvent evaporated under vacuum. The obtained crude was purified by silica gel flash column chromatography. In some cases, further purification by preparative TLC was necessary.

(*R*p)- and (*S*p)-3'-Deoxyadenosine 5'-*O*-phenyl-(benzoxy-*L*-alaninyl)-phosphate (7a). Prepared according to general procedure **C** using 3'-deoxyadenosine (**1**) (0.05 g, 0.20 mmol) in anhydrous THF (4 mL), *N*-methylimidazole (0.080 µL, 1.0 mmol), phenyl(benzyloxy-L-alaninyl) phosphorochloridate (**4a**) (0.021 g, 0.6 mmol) in THF (2.4 mL) Purification by Biotage Isolera One (cartridge SNAP 25g, 25 mL/min, CH_3OH/CH_2Cl_2 1-8% 10 CV, 8% 5 CV) and preparative TLC (1000 µM, eluent system CH_3OH/CH_2Cl_2 5/95) afforded the title compound **7a** as a white solid (0.032 g, 28%).^{31}P NMR (202 MHz, CD_3OD) δ_P 3.91, 3.73.^{1}H NMR (500 MHz, $CDCl_3$) δH 8.26 (s, 0.5H, H-8), 8.24 (s, 0.5H, H-8), 8.22 (s, 0.5H, H-2), 8.21 (s, 0.5H, H-2), 7.34-7.25 (m, 7H, Ar), 7.21-7.13 (m, 3H, Ar), 6.01 (d, *J* = 1.5 Hz, 0.5H, H-1'), 6.00 (d, *J* = 1.5 Hz, 0.5H, H-1'), 5.15-5.04 (m, 2H, C*H₂*Ph), 4.73-4.63 (m, 2H, H-2', H-4'), 4.43-4.35 (m, 1H, H-5'), 4.27-4.20 (m, 1H, H-5'), 4.03-3.91 (m, 1H, C*H*CH_3), 2.35-2.28 (m, 1H, H-3'), 2.09-2.02 (m, 1H, H-3'), 1.32 (d, *J* = 7.4 Hz, 1.5 H, CHCH_3), 1.28 (d, *J* = 7.4 Hz, 1.5 H, CHCH_3). ^{13}C NMR (125 MHz, CD_3OD) δ_C 174.84 (d, $^3J_{C-P}$ = 4.5 Hz, C=O), 174.63 (d, $^3J_{C-P}$ = 4.5 Hz, C=O), 157.32 (C-6), 157.31 (C-6), 153.86 (C-2), 153.84 (C-2), 152.13 (C-4), 152.07 (C-4), 150.20 (C-Ar), 150.18 (C-Ar), 140.47 (C-8), 137.26 (C-Ar), 137.19 (C-Ar), 130.76 (CH-Ar), 130.74 (CH-Ar), 129.57 (CH-Ar), 129.32 (CH-Ar), 129.31 (CH-Ar), 129.29 (CH-Ar), 129.26 (CH-Ar), 126.16 (CH-Ar), 126.14 (CH-Ar), 121.46 (d, $^3J_{C-P}$ = 4.7 Hz, CH-Ar), 121.38 (d, $^3J_{C-P}$ = 4.7 Hz, CH-Ar) 120.54 (C-5), 120.53 (C-5), 93.24 (C-1'), 93.18 (C-1'), 80.43 (d, $^3J_{C-P}$ = 3.6 Hz, C-4'), 80.36 (d, $^3J_{C-P}$ = 3.6 Hz, C-4'), 76.62 (C-2'), 68.62 (d, $^2J_{C-P}$ = 5.3 Hz, C-5'), 68.30 (d, $^2J_{C-P}$ = 5.3 Hz, C-5'), 67.95 (*C*H_2Ph), 67.92 (*C*H_2Ph), 51.74 (*C*HCH_3), 51.60 (*C*HCH_3), 34.91 (C-3'), 34.70 (C-3'), 20.45 (d, $^3J_{C-P}$ = 7.0 Hz, CH*C*H_3), 20.28 (d, $^3J_{C-P}$ = 7.0 Hz, CH*C*H_3). Reverse-phase HPLC eluting with H_2O/CH_3CN from 100/10 to 0/100 in 30 minutes, F = 1mL/min, λ = 254 nm, t_R 13.56 and 13.75 min. $C_{26}H_{29}N_6O_7P$ required *m/z* 568.2 [M]. MS (ES+) found *m/z* 569.2 $[M+H]^+$, 591.2 $[M+Na]^+$, 1159.4 $[2M+Na]^+$.

The two diasteroisomers **7a-*R*p** and **7a-*S*p** were separated via Biotage Isolera One (cartridge SNAP-Ultra C18 12 g, F: 12 mL/min, isocratic eluent system: H_2O/CH_3OH 45/55 in 30 min, 150 mg sample) to obtain:

7a-*R*$_p$ as fast eluting isomer (76 mg). ^{31}P NMR (202 MHz, CD_3OD) δ_P 3.91.^{1}H NMR (500 MHz, $CDCl_3$) δ_H 8.26 (s, 1H, H-8), 8.22 (s, 1H, H-2), 7.37-7.25 (m, 7H, Ar), 7.22-7.12 (m, 3H, Ar), 6.01 (d, *J* = 1.5 Hz, 1H, H-1'), 5.12 (AB q, J_{AB} = 12.0 Hz, 2H, C*H*$_2$Ph), 4.74-4.70 (m, 1H, H-2'), 4.69-4.62 (m, 1H, H-4'), 4.44-4.38 (m, 1H, H-5'), 4.28-4.21 (m, 1H, H-5'), 3.99-3.90 (m, 1H, C*H*CH_3), 2.35-2.27 (m, 1H, H-3'), 2.09-2.02 (m, 1H, H-3'), 1.29 (d, *J* = 7.0 Hz, 3H, CHC*H*$_3$). HPLC Reverse-phase HPLC eluting with H_2O/CH_3CN from 90/10 to 0/100 in 30 minutes, F= 1 mL/min, λ = 254 nm, showed one peak with tR 13.56 min.

and **7a-*S*$_p$** as slow eluting isomer (61 mg). ^{31}P NMR (202 MHz, CD_3OD) δ_P 3.73.^{1}H NMR (500 MHz, $CDCl_3$) δ_H 8.24 (s, 1H, H-8), 8.22 (s, 1H, H-2), 7.36-7.26 (m, 7H, Ar), 7.22-7.13 (m, 3H, Ar), 6.01 (d, *J* = 1.5 Hz, 1H, H-1'), 5.08 (AB q, J_{AB} = 12.0 Hz, 2H, C*H*$_2$Ph), 4.70-4.67 (m, 1H, H-2'), 4.66-4.60 (m, 1H, H-4'), 4.41-4.35 (m, 1H, H-5'), 4.26-4.19 (m, 1H, H-5'), 4.02-3.94 (m, 1H, C*H*CH_3), 2.36-2.27 (m, 1H, H-3'), 2.08-2.01 (m, 1H, H-3'), 1.34-1.30 (m, 3H, CHC*H*$_3$). HPLC Reverse-phase HPLC eluting with H_2O/CH_3CN from 90/10 to 0/100 in 30 minutes, F= 1 mL/min, λ = 254 nm, t_R 13.75 min.

3'-Deoxyadenosine 5'-O-naphth-1-yl(benzoxy-L-alaninyl)-phosphate (7b). Prepared according to general procedure **C** using 3'-deoxyadenosine (0.15 g, 0.6 mmol) in THF (12 mL), *N*- methylimidazole (0.240 μL, 3.0 mmol), naphth-1-yl- (benzyloxy-*L*-alaninyl) phosphorochloridate (**4b**) (0.727 g, 1.8 mmol) in THF (7 mL). Purification by Biotage Isolera One (cartridge SNAP KP-SIL 50g, 100 mL/min, CH_3OH/CH_2Cl_2 1-10% 10 CV, 10% 5 CV) and preparative TLC (2000 μM, eluent system CH_3OH/CH_2Cl_2 5/95) afforded compound **7b** as white solid (0.045 g, 12%).^{31}P NMR (202 MHz, CD_3OD) δ_P 4.32, 4.14.^{1}H NMR (500 MHz, CD_3OD) δ_H 8.24 (s, 0.5H, H-8), 8.22 (s, 0.5H, H-8), 8.20 (s, 0.5H, H-2), 8.19 (s, 0.5H, H-2), 8.14-8.09 (m, 1H, Ar), 7.89-7.85 (m, 1H, Ar), 7.70-7.67 (m, 1H, Ar), 7.53-7.42 (m, 3H, Ar), 7.39-7.34 (m, 1H, Ar), 7.31-7.25 (m, 5H, Ar), 5.99 (d, *J* = 2.0 Hz, 0.5H, H-1'), 5.98 (d, *J* = 2.0 Hz, 0.5H, H-1'), 5.10-5.01 (m, 2H, C*H*$_2$Ph), 4.72-4.61 (m, 2H, H-2', H-4'), 4.47-4.40 (m, 1H, H-5'), 4.33-4.24 (m, 1H, H-5'), 4.09-3.98 (m, 1H, C*H*CH_3) 2.35-2.26 (m, 1H, H-3'), 2.07-1.98 (m, 1H, H-3'), 1.30-1.24 (m, 3H, CHC*H*$_3$). ^{13}C NMR (125 MHz, CD_3OD) δ_C 174.85 (d, $^3J_{C-P}$ = 3.7 Hz, C=O), 174.56 (d, $^3J_{C-P}$ = 3.7 Hz, C=O), 157.33 (C6), 157.31 (C6), 153.87 (C2), 153.85 (C2), 150.24 (C4), 150.23 (C4), 147.91 (d, $^3J_{C-P}$ = 7.5 Hz, C-Ar), 147.95, (d, $^3J_{C-P}$ = 7.5 Hz, C-Ar), 140.56 (C-8), 140.50 (C-8), 137.22 (C-Ar), 137.17 (C-Ar), 136.28 (C-Ar), 129.55 (CH-Ar), 129.53 (CH-Ar), 129.30 (CH-Ar),

129.25 (CH-Ar), 128.88 (CH-Ar), 128.82 (CH-Ar), 127.91 (d, $^{2}J_{C\text{-}P}$ = 6.2 Hz, C-Ar), 127.83 (d, $^{2}J_{C\text{-}P}$ = 6.2 Hz, C-Ar), 127.77 (CH-Ar), 127.75 (CH-Ar), 127.49 (CH-Ar), 127.45 (CH-Ar), 126.48 (CH-Ar), 126.47 (CH-Ar), 126.02 (CH-Ar), 125.97 (CH-Ar), 122.77 (CH-Ar), 122.63 (CH-Ar), 120.58 (C5), 120.53 (C5), 116.35 (d, $^{3}J_{C\text{-}P}$ = 3.7 Hz, CH-Ar), 116.15 (d, $^{3}J_{C\text{-}P}$ = 3.7 Hz, CH-Ar), 93.22 (C1'), 93.20 (C1'), 80.30 (d, $^{3}J_{C\text{-}P}$ = 2.7 Hz, C4'), 80.24 (d, $^{3}J_{C\text{-}P}$ = 2.7 Hz, C-4'), 76.51 (C-2'), 76.44 (C-2'), 68.87 (d, $^{2}J_{C\text{-}P}$ = 5.2 Hz, C-5'), 68.64 (d, $^{2}J_{C\text{-}P}$ = 5.2 Hz, C5'), 67.93 (CH_2Ph), 51.82 ($CHCH_3$), 51.73 ($CHCH_3$), 35.01 (C-3'), 34.76 (C-3'), 20.41 (d, $^{3}J_{C\text{-}P}$ = 6.7 Hz, $CHCH_3$), 20.22 (d, $^{3}J_{C\text{-}P}$ = 6.7 Hz, $CHCH_3$).Reverse-phase HPLC eluting with H_2O/CH_3CN from 90/10 to 0/100 in 30 min., F = 1ml/min, λ = 280 nm, t_R 15.34 and 15.55 min. $C_{30}H_{31}N_6O_7P$ required *m/z* 618.58 [M]. (ES+) found *m/z* 619.2 $[M+H]^+$, 641.2 $[M+Na]^+$, 1259.4 $[2M+Na]^+$

3'-Deoxyadenosine 5'-*O*-(benzoxy-glycinyl)-phosphate (7c). Prepared according to general procedure **C** using 3'-deoxyadenosine (**1**) (0.05 g, 0.20 mmol) in THF (4 mL), *N*-methylimidazole (80 μL, 1.0 mmol), phenyl (benzyloxy-glycinyl) phosphorochloridate (**4c**) (204 mg, 0.6 mmol) in THF (2.4 mL). Purification by flash column chromatography (eluent system CH_3OH/CH_2Cl_2 0/100 to 6/94) and preparative TLC (500 μM, eluent system CH_3OH/CH_2Cl_2 5/95) afforded compound **7c** as a white solid (0.021 g, 19%).^{31}P NMR (202 MHz, CD_3OD) δ_P 5.12, 4.91.^{1}H NMR (500 MHz, CD_3OD) δ_H 8.27 (s, 0.5H, H-8), 8.24 (s, 0.5H, H-8), 8.22 (s, 0.5H, H-2), 8.21 (s, 0.5H, H-2), 7.37-7.26 (m, 7H, Ph), 7.22-7.13 (m, 3H, Ph), 6.02 (d, *J* = 1.8 Hz, 0.5H, H-1'), 6.00 (d, *J* = 1.8 Hz, 0.5H, H-1'), 5.15-5.12 (m, 2H, CH_2Ph), 4.73-4.64 (m, 2H, H2', H-4'), 4.50-4.39 (m, 1H, H-5'), 4.36-4.24 (m, 1H, H-5'), 3.53-3.71 (m, 2H, CH_2NH), 2.39-2.25 (m, 1H, H-3'), 2.13-2.02 (m, 1H, H-3').^{13}C NMR (125 MHz, CD_3OD) δ_C 172.30 (d, $^{3}J_{C\text{-}P}$ = 5.0 Hz, C=O), 172.27 (d, $^{3}J_{C\text{-}P}$ = 5.0 Hz, C=O), 157.34 (C-6), 157.32 (C-6), 153.88 (C-2), 153.87 (C-2), 152.08 (d, $^{3}J_{C\text{-}P}$ = 7.5 Hz, C-Ar), 152.05 (d, $^{3}J_{C\text{-}P}$ = 7.5 Hz, C-Ar), 150.20 (C-4), 150.19 (C-4), 140.52 (C-8), 140.42 (C-8), 137.15 (C-Ar), 130.79 (CH-Ar), 129.57 (CH-Ar), 129.55 (CH-Ar), 129.35 (CH-Ar), 129.34 (CH-Ar), 129.33 (CH-Ar), 126.22 (CH-Ar), 121.44 (d, $^{3}J_{C\text{-}P}$ = 3.7 Hz, CH-Ar), 121.40 (d, $^{3}J_{C\text{-}P}$ = 3.7 Hz, CH-Ar), 120.51 (C-5), 120.49 (C-5), 93.19, 93.14 (C-1'), 80.46 (d, $^{3}J_{C\text{-}P}$ = 4.6 Hz, C-4'), 80.39 (d, $^{3}J_{C\text{-}P}$ = 4.6 Hz, C-4'), 76.66 (C-2'), 68.68 (d, $^{2}J_{C\text{-}P}$ = 5.4 Hz, C-5'), 68.24 (d, $^{2}J_{C\text{-}P}$ = 5.4 Hz, C-5'), 67.95 (CH_2Ph), 67.93 (CH_2Ph), 43.90 (CH_2NH), 43.83 (CH_2NH), 34.83 (C-3'), 34.54 (C-3'). Reverse-phase HPLC eluting with H_2O/CH_3CN from 100/10 to 0/100 in 30 min, F = 1mL/min, λ = 254 nm, t_R 12.98 min. $C_{25}H_{27}N_6O_7P$ required *m/z* 554.2 [M] (ES+) m/z found: 555.2 $[M+H]^+$,

577.2 $[M+Na]^+$, 1131.4 $[2M+Na]^+$

3’-Deoxyadenosine 5’-*O*-naphth-1-yl-(pent-1-oxy-L-leucinyl)-phosphate (7d). Prepared according to general procedure **C** using 3’- deoxyadenosine (0.048 g, 0.19 mmol) in THF (4 mL), *N*-methylimidazole (76 µL, 0.95 mmol), naphth-1-yl(pentyl- 1-oxy-L-leucinyl) phosphorochloridate (**4d**) (0.25 g, 0.6 mmol) in THF (2.4 mL). Purification by Biotage Isolera One, cartridge SNAP 25 g, 25 mL/min, CH_3OH/CH_2Cl_2 2-20% (15 CV), 20% (5 CV) and preparative TLC (1000 µM, eluent system CH_3OH/CH_2Cl_2 4/96) afforded compound **7d** as white solid (0.027 g, 22%).^{31}P NMR (202 MHz, CD_3OD) δ_P 4.64, 4.37.^{1}H NMR (500 MHz, CD_3OD) δ_H 8.28 (s, 0.5H, H-8), 8.25 (s, 0.5H, H-8), 8.21 (s, 0.5H, H-2), 8.20 (s, 0.5H, H-2), 8.17-8.12 (m, 1H, H-8 Napht), 7.88-7.83 (m, 1H, H-5 Napht), 7.69-7.66 (m, 1H, H-4 Napht), 7.54-7.42 (m, 3H, H-2, H-6, H-7 Napht), 7.40-7.35 (m, 1H, H-3 Napht), 6.01 (d, *J* = 2.1 Hz, 0.5H, H-1’), 6.00 (d, *J* = 2.1 Hz, 0.5H, H-1’), 4.47-4.67 (m, 2H, H-2’, H-4’), 4.55-4.44 (m, 1H, H-5’), 4.43-4.31 (m, 1H, H-5’), 4.00-3.87 (m, 3H, C*H*NH *and* C*H2*O), 2.44-2.30 (m, 1H, H-3’), 2.14-2.04 (m, 1H, H-3’), 1.66-1.39 (m, 5H, $CH_2CH(CH_3)_2$, $CH_2CH_2(CH_2)_2CH_3$), 1.1.28-1.21 (m, 4H, $(CH_2)_2CH_2CH_2CH_3$), 0.86-0.81 (m, 3H, $(CH_2)_4CH_3$), 0.81-0.68 (m, 6H, $CH(CH_3)_2$). ^{13}C NMR (125 MHz, CD3OD) δ_C 175.42 (d, $^3J_{C\text{-}P}$ = 2.5 Hz, C=O), 175.04 (d, $^3J_{C\text{-}P}$ = 2.5 Hz, C=O), 157.32 (C6), 153.87 (C2), 153.86 (C2), 150.23 (C4), 147.97 (d, $^3J_{C\text{-}P}$ = 6.2 Hz, C-Ar), 140.55 (C8), 136.30 (C-Ar), 136.29 (C-Ar), 128.89 (CH-Ar), 128.84 (CH-Ar), 127.95 (C-Ar), 127.91 (C-Ar), 127.84 (C-Ar), 127.78 (CH-Ar), 127.76 (CH-Ar), 127.46 (CH-Ar), 126.50 (C-Ar), 126.48 (C-Ar), 126.46 (C-Ar), 126.01 (CH-Ar), 125.91 (CH-Ar), 122.80 (CH-Ar), 122.70 (CH-Ar), 120.58 (C5), 120.56 (C5), 116.40 (d, $^3J_{C\text{-}P}$ = 3.7 Hz, CH-Ar), 116.01 (d, $^3J_{C\text{-}P}$ = 3.7 Hz, CH-Ar), 93.31 (C-1’), 93.27 (C-1’), 80.35 (d, $^3J_{C\text{-}P}$ = 3.5 Hz, C-4’), 80.29 (d, $^3J_{C\text{-}P}$ = 3.5 Hz, C4’), 76.54 (C2’), 76.50 (C2’), 69.07 (d, $^2J_{C\text{-}P}$ = 5.5 Hz, C5’), 68.85 (d, $^2J_{C\text{-}P}$ = 5.5 Hz, C-5’), 66.33 (*C*H2O *n*-Pen), 66.32 (*C*H2O), 54.81 (*C*HNH), 54.71 (*C*HNH), 44.22 (d, $^3J_{C\text{-}P}$ = 7.6 Hz, *C*H2CH(CH3)2), 43.93 (d, $^3J_{C\text{-}P}$ = 7.6 Hz, *C*H2CH(CH3)2), 35.15 (C-3’), 34.86 (C-3’), 29.32 ($CH2(CH_2CH_2)_2CH_3$), 29.30 ($CH_2(CH_2CH_2)_2CH_3$), 29.11 ($CH_2(CH2)_3CH_3$), 25.67 ($CH(CH_3)_2$), 25.45 ($CH(CH_3)_2$), 23.30 ($CH2(CH2)_3CH_3$), 23.12 ($CH(CH_3)_2$), 23.02 ($CH(CH_3)_2$), 22.0 ($CH(CH_3)_2$), 21.78 ($CH(CH_3)_2$), 14.28 ($CH_2CH_2CH_2CH_2CH_3$). $C_{31}H_{41}N_6O_7P$ required *m/z* 640.3 [M]. (ES+) found *m/z* 641.3 $[M+H]^+$, 663.3 $[M+Na]^+$, 1303.6 $[2M+Na]^+$. Reverse-phase HPLC eluting with H_2O/CH_3CN from 100/10 to 0/100 in 30 min., F = 1 mL/min, λ = 200 nm, t_R 18.90 min.

3’-Deoxyadenosine 5’-*O*-naphth-1-yl(methoxy-2,2-dimethylglycinyl)-phosphate (7e).

Prepared according to general procedure **C** using 3'-deoxyadenosine (0.15 g, 0.6 mmol) in THF (4 mL), *N* methylimidazole (240 µL, 3.0 mmol), naphth-1-yl-(methoxy-2,2-dimethylglycinly) phosphorochloridate (**4e**) (0.61 g, 1.8 mmol) in THF (2.4 mL). Purification by Biotage Isolera One, cartridge SNAP KP-SIL 50 g, 100 mL/min, CH_3OH/CH_2Cl_2 2-20% (10 CV), 20% (5 CV) and preparative TLC (1000 µM, eluent system CH_3OH/CH_2Cl_2 4/96) afforded compound **7e** as a white solid (0.02 g, 6%).^{31}P NMR (202 MHz, CD_3OD) δ_P 2.73.^{1}H NMR (500 MHz, CD_3OD) δH 8.28 (s, 0.5H, H-8), 8.25 (s, 0.5H, H-8), 8.21 (s, 0.5H, H-2), 8.19 (s, 0.5H, H-2), 8.18-8.14 (m, 1H, H-8 Napht), 7.90-7.84 (m, 1H, H-5 Napht), 7.71-7.66 (m, 1H, H-4 Napht), 7.53-7.47 (m, 3H, H-2, H-6, H-7 Napht), 7.41-7.35 (m, 1H, H-3 Napht), 6.03 (d, *J* = 2.1 Hz, 0.5H, H-1'), 5.99 (d, *J* = 2.1 Hz, 0.5H, H-1'), 4.76-4.67 (m, 2H, H-2', H-4'), 4.52-4.44 (m, 1H, H-5'), 4.42-4.33 (m, 1H, H-5'), 3.65 (s, 1.5H, OCH3), 3.64 (s, 1.5H, OCH3), 2.48-2.41 (m, 0.5H, H-3'), 2.37-2.30 (m, 0.5H, H-3'), 2.15-2.09 (m, 0.5H, H-3'), 2.08-2.02 (m, 0.5H, H-3'), 1.47-1.44 (m, 6H, $C(CH_3)_2$).^{13}C NMR (125 MHz, CD_3OD) δ_C 177.25 (d, $^3J_{C\text{-}P}$ = 3.7 Hz, C=O), 157.53 (C-6), 157.51 (C-6), 153.86 (C-2), 150.28 (C-4), 150.25 (C-4), 148.06 (d, $^3J_{C\text{-}P}$ = 7.5 Hz, C-Ar), 148.04 (d, $^3J_{C\text{-}P}$ = 7.5 Hz, C-Ar), 140.67 (C-8), 140.60 (C-8), 136.28 (C-Ar), 136.27 (C-Ar), 128.82 (CH-Ar), 128.80 (CH-Ar), 127.93 (d, $^2J_{C\text{-}P}$ = 6.25 Hz, C-Ar), 127.92 (d, $^2J_{C\text{-}P}$ = 6.25 Hz, C-Ar), 127.71 (CH-Ar), 127.69 (CH-Ar), 127.32 (CH-Ar), 126.44 (CH-Ar), 125.84 (CH- Ar), 122.93 (CH-Ar), 120.56 (C5), 120.50 (C5), 116.38 (d, $^3J_{C\text{-}P}$ = 3.75 Hz, CH-Ar), 116.36 (d, $^3J_{C\text{-}P}$ = 3.7 Hz, CH-Ar), 93.25 (C-1'), 80.40 (d, $^3J_{C\text{-}P}$ = 8.0 Hz, C-4'), 80.33 (d, $^3J_{C\text{-}P}$ = 8.0 Hz, C-4'), 76.57 (C-2'), 76.43 (C-2'), 68.99 (d, $^2J_{C\text{-}P}$ = 5.5 Hz, C-5'), 68.84 (d, $^2J_{C\text{-}P}$ = 5.5 Hz, C-5'), 53.01 (O*C*H3), 35.22 (C-3'), 34.90 (C-3'), 27.85 (d, $^3J_{C\text{-}P}$ = 6.0 Hz, $(CH_3)_2$), 27.80 (d, $^3J_{C\text{-}P}$ = 6.0 Hz, $(CH_3)_2$), 27.60 (d, $^3J_{C\text{-}P}$ = 6.0 Hz, $(CH_3)_2$), 27.56 (d, $^3J_{C\text{-}P}$ = 6.0 Hz, $(CH_3)_2$). Reverse-phase HPLC eluting with H_2O/CH_3CN from 100/10 to 0/100 in 30 minutes, F = 1 mL/min, λ = 254 nm, t_R 15.58 min. $C_{25}H_{29}N_6O_7P$ required *m/z* 556.51 [M] (ES+) found *m/z* 557.2 $[M+H]^+$, 579.2 $[M+Na]^+$, 1135.4 $[2M+Na]^+$.

3'-Deoxyadenosine 5'-*O*-ethyl-3-(2-hydroxyphenyl)propanoyl-(benzoxy-L- alaninyl)-phosphate (7f). Prepared according to general procedure **C** using 3'-deoxyadenosine (**1**) (0.15 g, 0.6 mmol) in THF (4 mL), *N*-methylimidazole (320 µL, 4.2 mmol), ethyl 3-(2-hydroxyphenyl)propanoyl-L-alanine benzyl ester phosphorochloridate (**4f**) (1.14 g, 2.5 mmol) in THF (2.4 mL). Purification by Biotage Isolera One, cartridge ZIP Sphere 80 g, 100 mL/min, CH_3OH/CH_2Cl_2 1-8% (10 CV), 8% (5 CV) and preparative TLC (1000 µM, eluent system CH_3OH/CH_2Cl_2 5/95) afforded the title compound **7f** as a white solid (0.032 g, 18%). ^{3}1P NMR

(202 MHz, CD_3OD) δ_P 3.95, 3.65. ^{1}H NMR (500 MHz, CD_3OD) δ_H 8.25 (s, 0.5H, H-8), 8.21 (s, 1H, H-8, H-2), 8.20 (s, 0.5H, H-2), 7.35-7.29 (m, 6H, Ar), 7.25-7.21 (m, 1H, Ar), 7.16-7.07 (m, 2H, Ar), 6.00 (d, *J* = 1.9 Hz, 0.5H, H-1'), 5.98 (d, *J* = 1.9 Hz, 0.5H, H-1'), 5.17-5.05 (m, 2H, CH_2Ph), 4.76-4.73 (m, 0.5H, H-2'), 4.70-4.59 (m, 1.5H, H-2', H-4'), 4.45-4.34 (m, 1H, H-5'), 4.30-4.22 (m, 1H, H-5'), 4.08-3.96 (m, 3H, $CH_2CH_2OCH_2CH_3$, CHNH), 2.98-2.92 (m, 2H, $CH_2CH_2OCH_2CH_3$), 2.62-2.56 (m, 2H, $CH_2CH_2OCH_2CH_3$), 2.40-2.29 (m, 1H, H-3'), 2.11- 2.03 (m, 1H, H-3'), 1.36 (d, *J* = 6.9 Hz, 1.5 H, $CHCH_3$), 1.33 (d, *J* = 6.9 Hz, 1.5 H, $CHCH_3$), 1.17 (t, *J* = 7.0 Hz, 1.5 H, $CH_2CH_2OCH_2CH_3$), 1.16 (t, *J* = 7.0 Hz, 1.5 H, $CH_2CH_2OCH_2CH_3$). ^{13}C NMR (125 MHz, CD_3OD) δ_C 174.82 (d, $^3J_{C\text{-}P}$ = 3.7 Hz, C=O), 174.62 (C=O), 174.58 (C=O), 174.55 (d, $^3J_{C\text{-}P}$ = 3.7 Hz, C=O), 157.34 (C-6), 157.32 (C-6), 153.86 (C-2), 153.84 (C-2), 150.48 (d, $^3J_{C\text{-}P}$ = 2.5 Hz, C-Ar), 150.44 (C-4), 150.22 (d, $^3J_{C\text{-}P}$ = 2.5 Hz, C-Ar), 140.49 (C-8), 137.29 (C-Ar), 137.21 (C-Ar), 133.09 (d, $^2J_{C\text{-}P}$ = 7.5 Hz, C-Ar), 132.94 (d, $^2J_{C\text{-}P}$ = 7.5 Hz, C-Ar), 131.62 (CH-Ar), 131.59 (CH-Ar), 129.58 (CH-Ar), 129.34 (CH-Ar), 129.31 (CH-Ar), 129.28 (CH-Ar), 128.70 (d, $^3J_{C\text{-}P}$ = 5.0 Hz, CH-Ar), 128.69 (d, $^3J_{C\text{-}P}$ = 5.0 Hz, CH-Ar), 126.18 (CH-Ar), 121.02 (d, $^3J_{C\text{-}P}$ 2.5 Hz, CH-Ar), 120.49 (d, $^3J_{C\text{-}P}$ 2.5 Hz, CH-Ar), 120.58 (C-5), 93.28 (C-1'), 93.24 (C-1'), 80.32 (d, $^3J_{C\text{-}P}$ -P = 8.7 Hz, C-4'), 76.57 (C-2'), 68.86 (d, $^2J_{C\text{-}P}$ = 5.0 Hz, C-5'), 68.53 (d, $^2J_{C\text{-}P}$ = 5.0 Hz, C-5'), 67.98 (CH_2Ph), 67.95 (CH_2Ph), 61.57 ($CH_2CH_2OCH_2CH_3$), 51.76 ($CHCH_3$), 51.65 (CHCH3), 35.37 ($CH_2CH_2OCH_2CH_3$), 35.30 ($CH_2CH_2OCH_2CH_3$), 35.08 (C-3'), 34.85 (C-3'), 26.77 ($CH_2CH_2OCH_2CH_3$), 26.72 ($CH_2CH_2OCH_2CH_3$), 20.55 (d, $^3J_{C\text{-}P}$ = 6.2 Hz, $CHCH_3$), 20.33 (d, $^3J_{C\text{-}P}$ = 6.2 Hz, $CHCH_3$), 14.53 ($CH_2CH_2OCH_2CH_3$). Reverse-phase HPLC eluting with H_2O/CH_3CN from 100/10 to 0/100 in 30 minutes, F = 1 mL/min, λ = 280 nm, t_R 15.45 min. $C_{31}H_{37}N_6O_9P$ required *m/z* 668.63 [M](ES+) found *m/z* 669.3 $[M+H]^+$ 691.3 $[M+Na]^+$.

3'- deoxyadenosine -5'-bis-*O*-[benzoxy-L-alaninyl)] phosphate (9). To a -5ºC cooled solution of 3'-deoxyadenosine (200 mg, 0.80 mmol) in trimethylphosphate (5 mL), $POCl_3$ (75 μL, 0.80 mmol) was added dropwise and the resulting mixture was stirred at this temperature for 4 h. The formation of the intermediate **8** was monitored by ^{31} P NMR. The mixture was then cooled at -78ºC and a solution of L-alanine *O*-benzyl ester *p*-TSA salt (1.4 g, 4.0 mmol) in anhydrous CH_2Cl_2 (5 mL) was added, followed by diisopropyl ethyl amine (1.4 mL, 8.0 mmol). The reaction mixture was then allowed to warm to rt and then it was stirred at this temperature for 20h. Water was then added, and the layers separated. The aqueous layer was extracted with CH_2Cl_2. The combined organic phases were washed with brine, dried over Na_2SO_4 and concentrated. The residue was purified by extraction and flash column chromatography (gradient elution of

CH_2Cl_2/MeOH=100/0 to 93/7) to give **9** as white foam (256 mg, 49 %). ^{31}P NMR (202 MHz, CD_3OD) δ_P 13.9. ^1H NMR (500 MHz, CD_3OD) δ_H 8.28 (s, 1H, H8), 8.22 (s, 1H, H-2), 7.37-7.26 (m, 10H, Ph), 6.00 (d, J = 1.9 Hz, 1H, H-1'), 5.15-5.05 (m, 4H, OCH_2Ph), 4.74-4.70 (m, 1H, H-2'), 4.63-4.56 (m, 1H, H-4'), 4.24-4.18 (m, 1H, H-5'), 4.11-4.05 (m, 1H, H-5'), 3.97-3.87 (m, 1H, C*H*CH_3), 2.35-2.27 (m, 1H, H-3'), 2.07-2.01 (m, 1H, H-3'), 1.34-1.27 (m, 3H, CHCH_3). ^{13}C NMR (125 MHz, CD_3OD) δ_C 175.40 (d, $^3J_{C\text{-}P}$ = 5.0 Hz, C=O), 175.36 (d, $^3J_{C\text{-}P}$ = 5.0 Hz, C=O), 157.36 (C-6), 153.91 (C-2), 150.25 (C-4), 140.64 (C-8), 137.33 (C-Ar), 137.29 (C-Ar), 129.58 (CH-Ar), 129.57 (CH-Ar), 129.33 (CH-Ar), 129.31 (CH-Ar), 129.29 (CH-Ar), 120.55 (C-5), 93.18 (C-1'), 80.67 (d, $^3J_{C\text{-}P}$ = 8.4 Hz, C-4'), 76.59 (C-2'), 67.90 (O*C*H_2Ph), 67.47 (d, $^2J_{C\text{-}P}$ = 5.2 Hz, C-5'), 51.14 (d, $^2J_{C\text{-}P}$ = 1.7 Hz, *C*HCH_3), 51.11 (d, $^2J_{C\text{-}P}$ = 1.7 Hz, *C*HCH_3), 35.08 (C-3'), 20.77 (d, $^3J_{C\text{-}P}$ = 6.5 Hz, CH*C*H_3), 20.59 (d, $^3J_{C\text{-}P}$ = 6.5 Hz, CH*C*H_3). $C_{30}H_{36}N_7O_8P$ required *m/z* 653.62 [M]. (ES+) found *m/z* 654.2 $[M + H]^+$, 676.2 $[M + Na]^+$, 1329.5 $[2M + Na]^+$ Reverse-phase HPLC eluting with H_2O/CH_3CN from 90/10 to 0/100 in 30 minutes, F=1ml/min, λ = 254 nm , showed one peak with t_R 13.87 min.

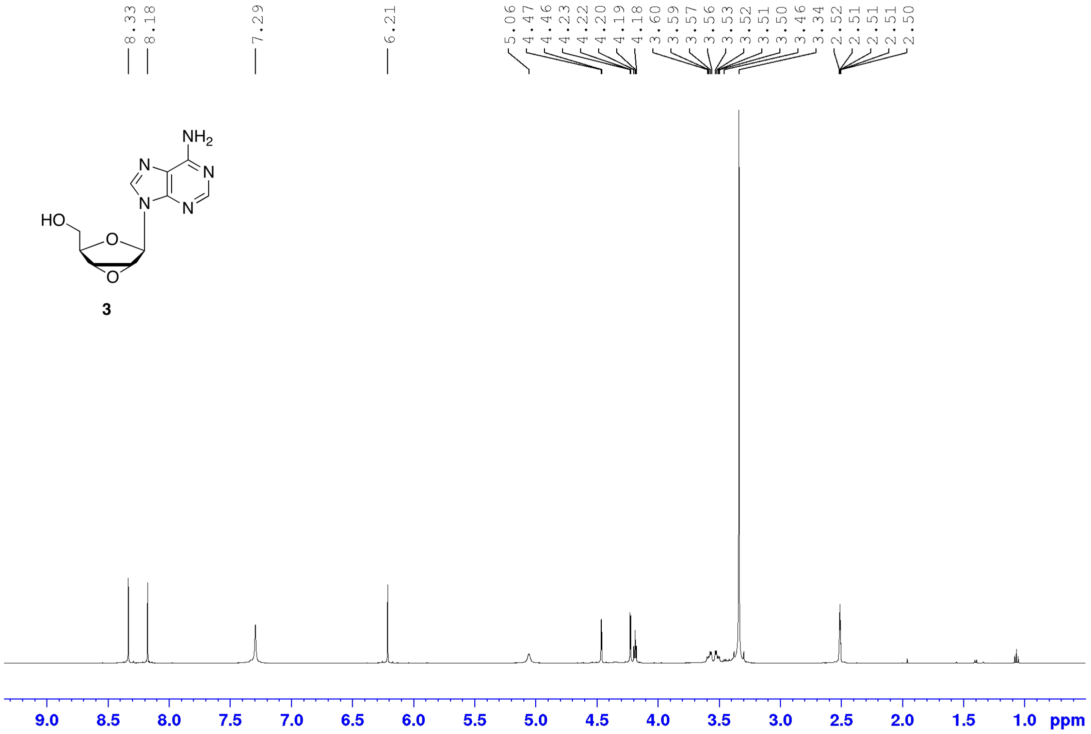

^{1}H-NMR (500MHz, DMSO-d$_6$)

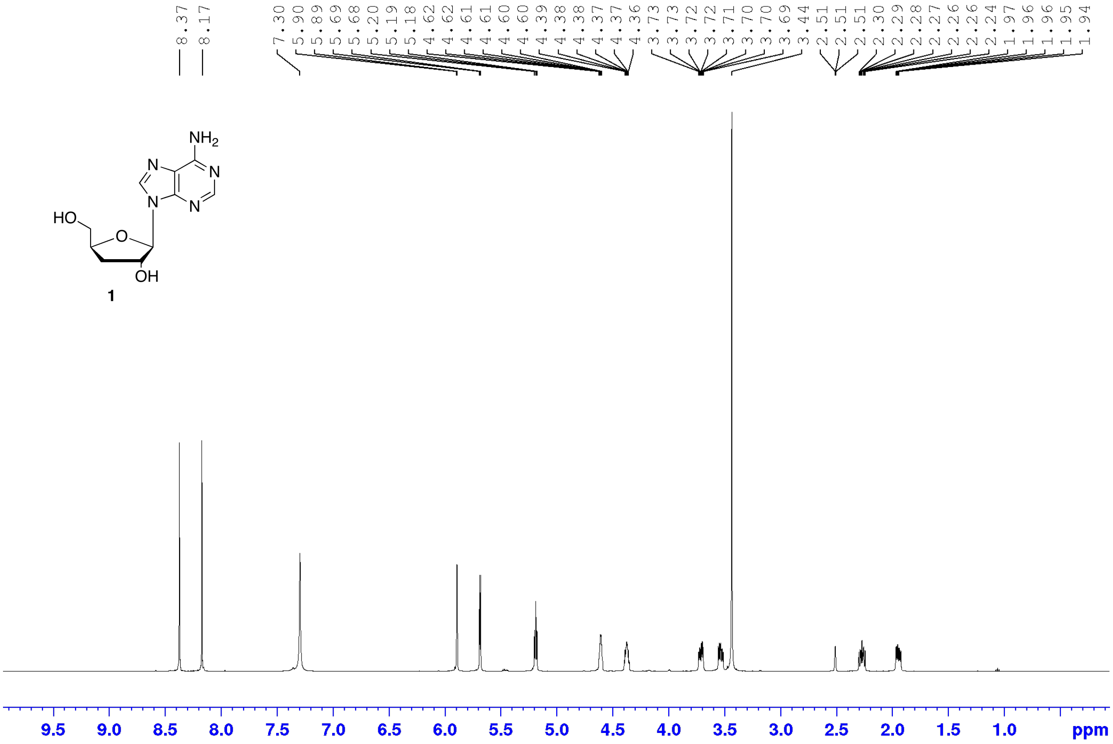

^{1}H-NMR (500MHz, DMSO-d$_6$)

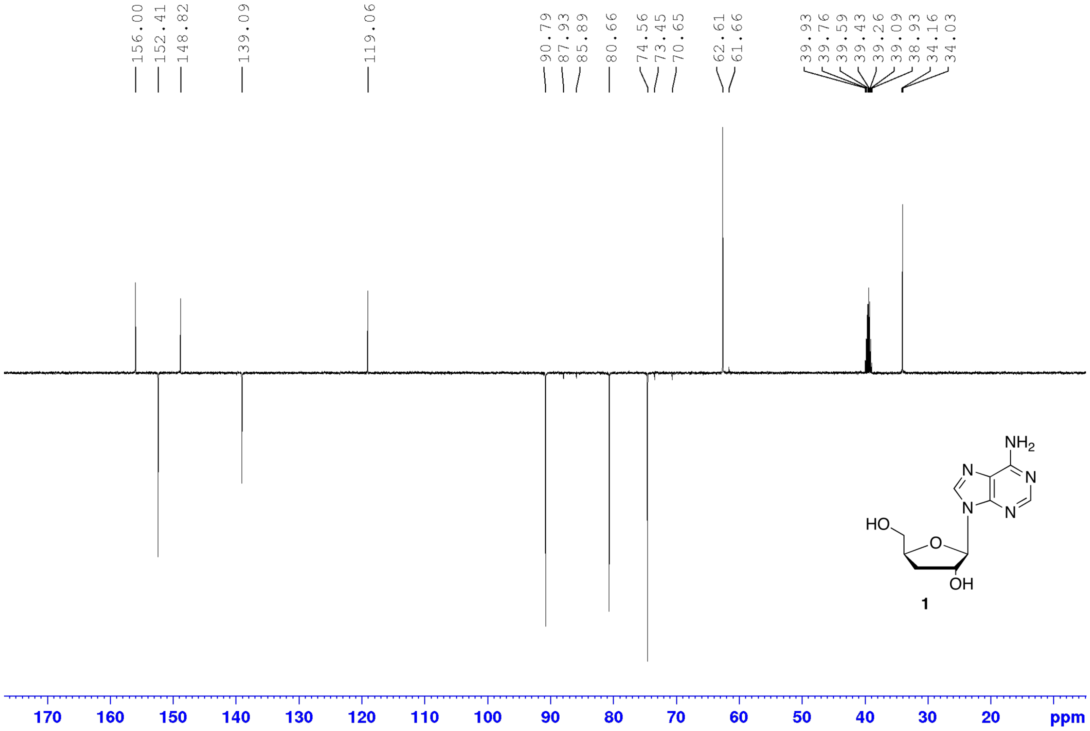

^{13}C-NMR (125MHz, DMSO-d$_6$)

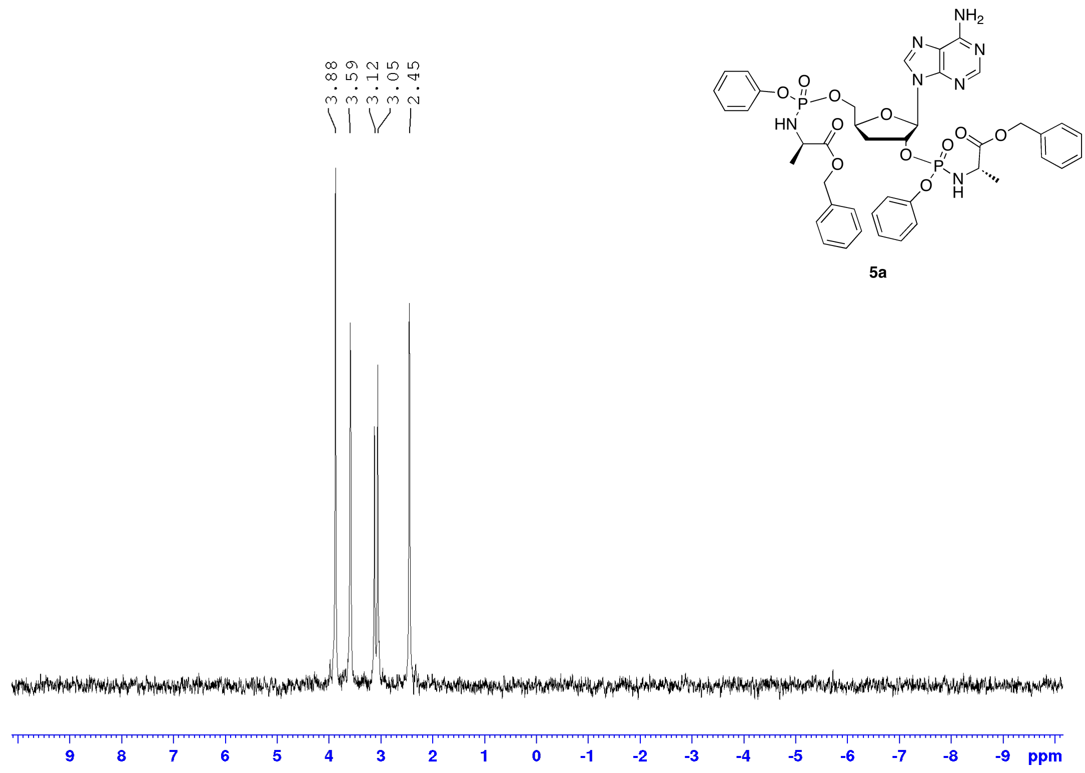

^{31}P-NMR (202MHz, CD_3OD)

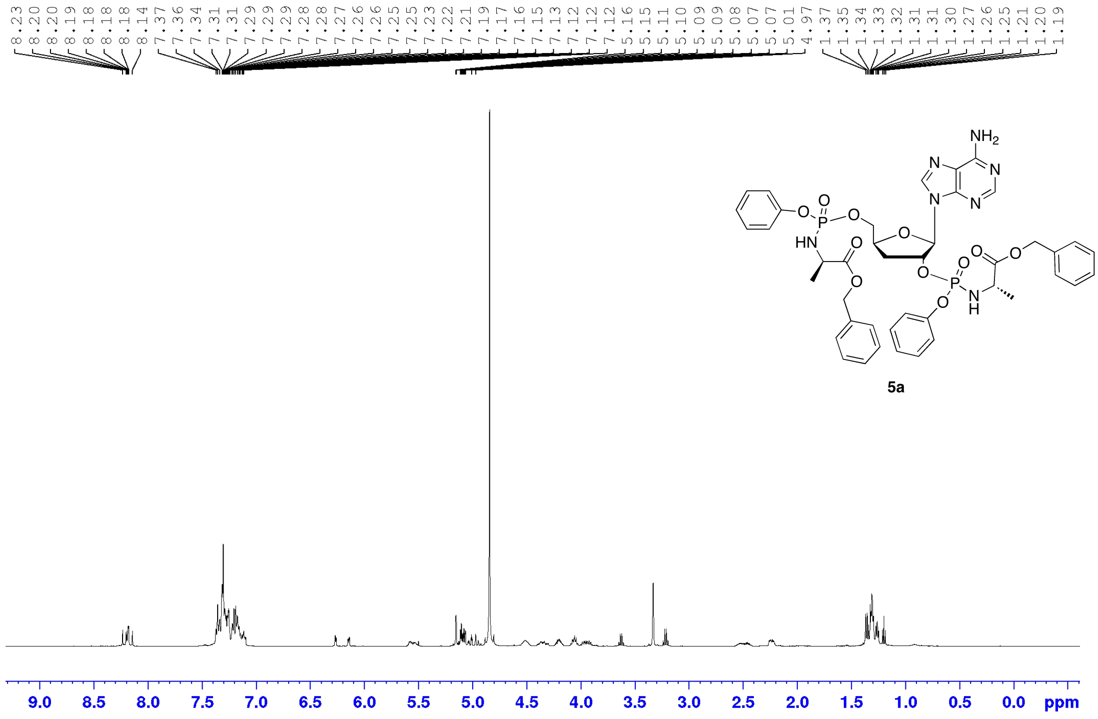

^{1}H-NMR (500MHz, CD_3OD)

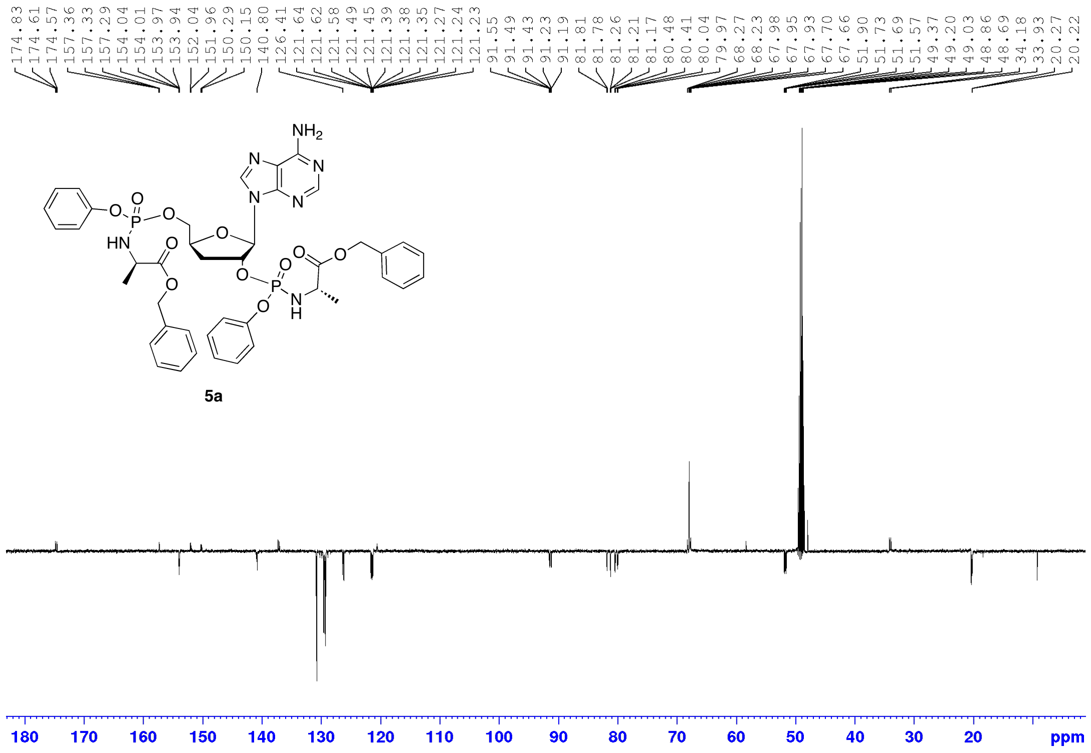

^{13}C-NMR (125MHz, CD_3OD)

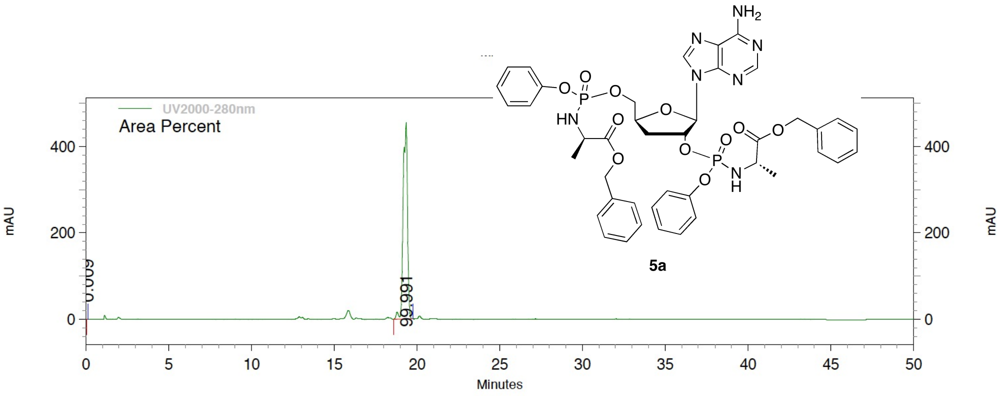

Retention Time	Area	Area %	Height	Height %
0.060	706	0.01	255	0.06
19.345	8202470	99.99	455030	99.94
Totals	8203176	100.00	455285	100.00

HPLC trace of **5a**

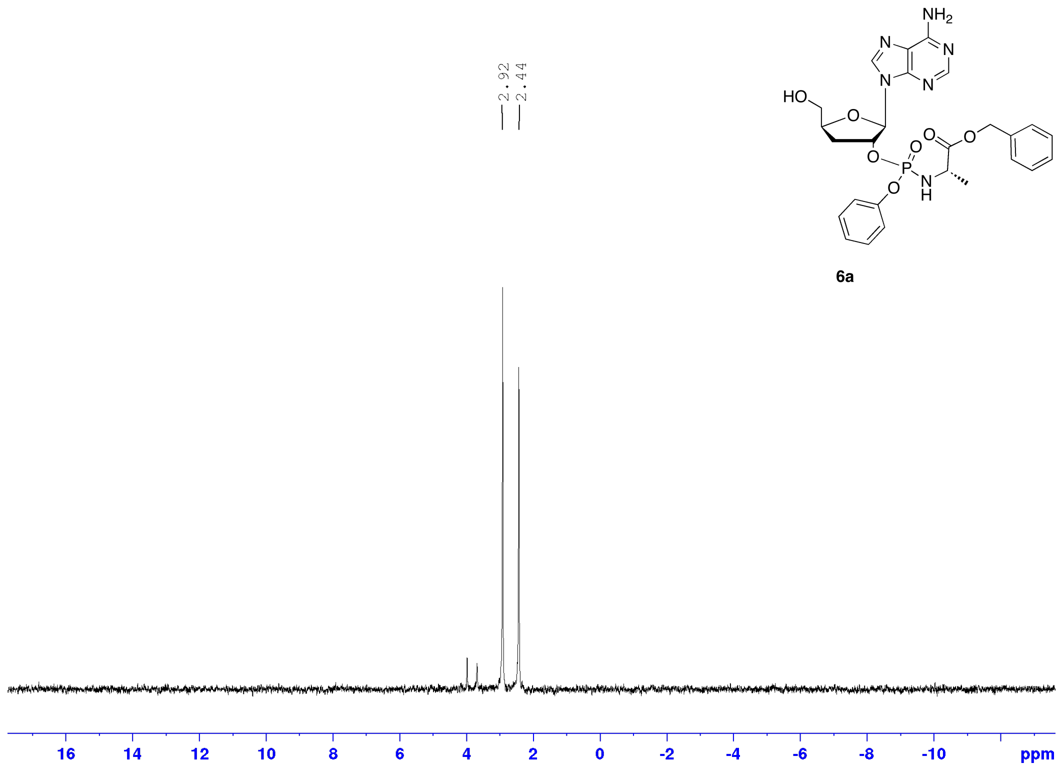

^{31}P-NMR (202MHz, CD_3OD)

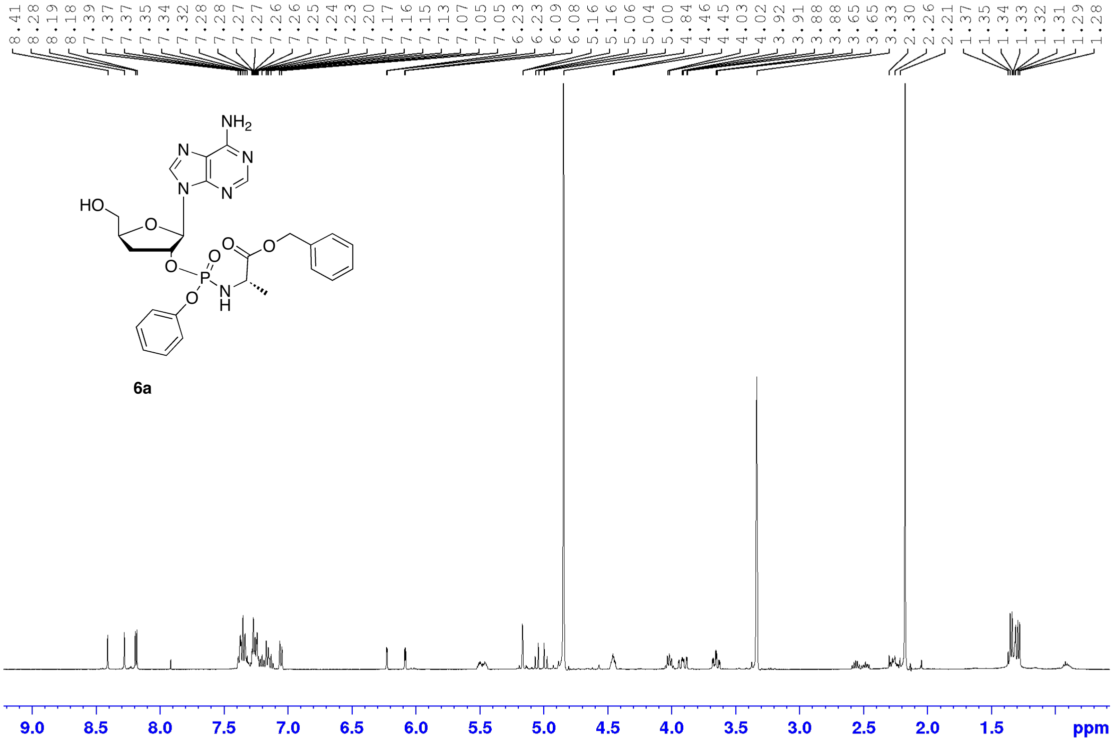

^{1}H-NMR (500MHZ, CD_3OD)

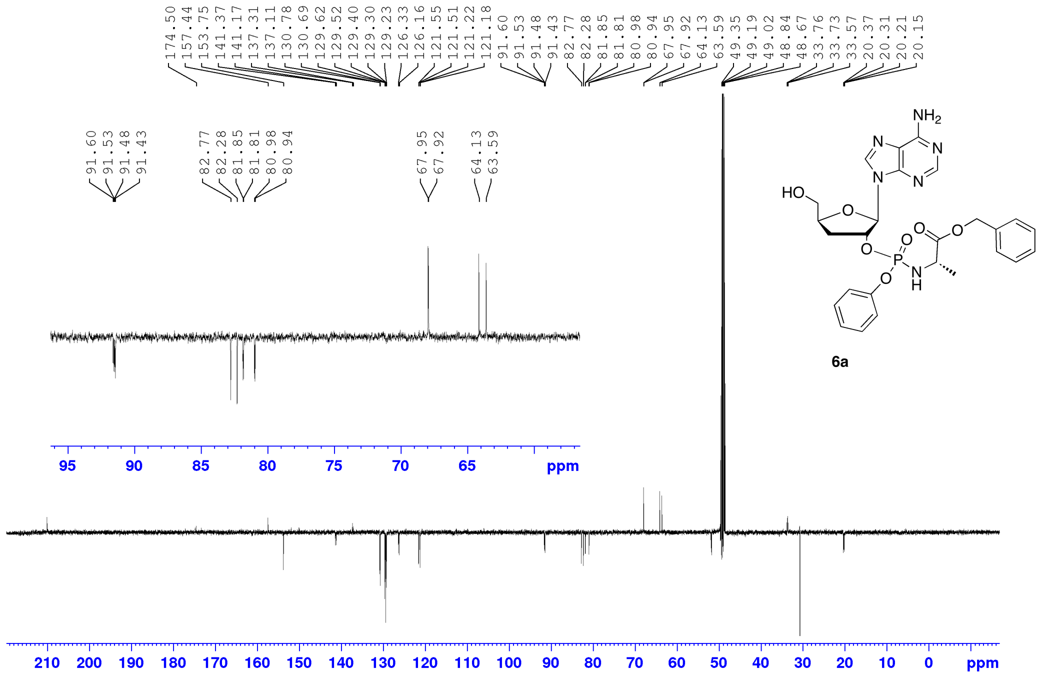

^{13}C-NMR (125MHz, CD_3OD)

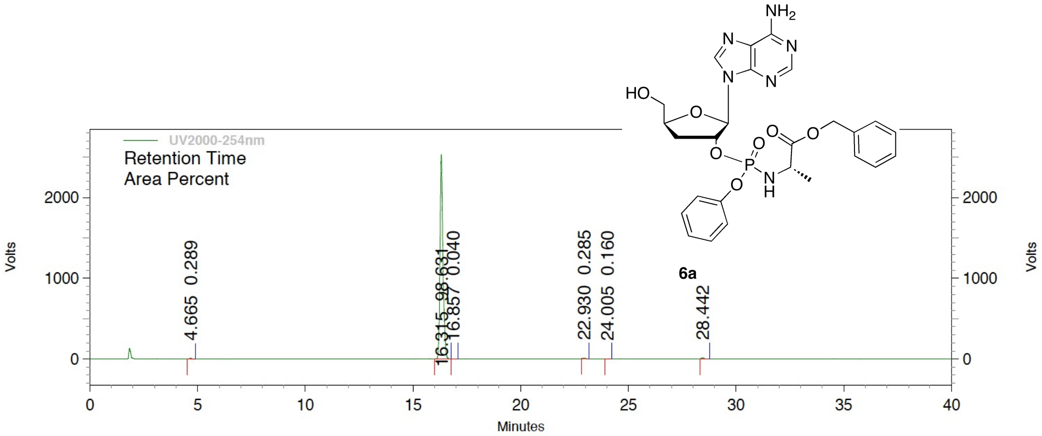

Retention Time	Area	Area %	Height	Height %
4.665	70142	0.29	8443	0.33
16.315	23899967	98.63	2530018	98.75
16.857	9599	0.04	795	0.03
22.930	68968	0.28	6775	0.26
24.005	38700	0.16	3959	0.15
28.442	144277	0.60	11976	0.47
Totals	24231653	100.00	2561966	100.00

HPLC trace of **6a**

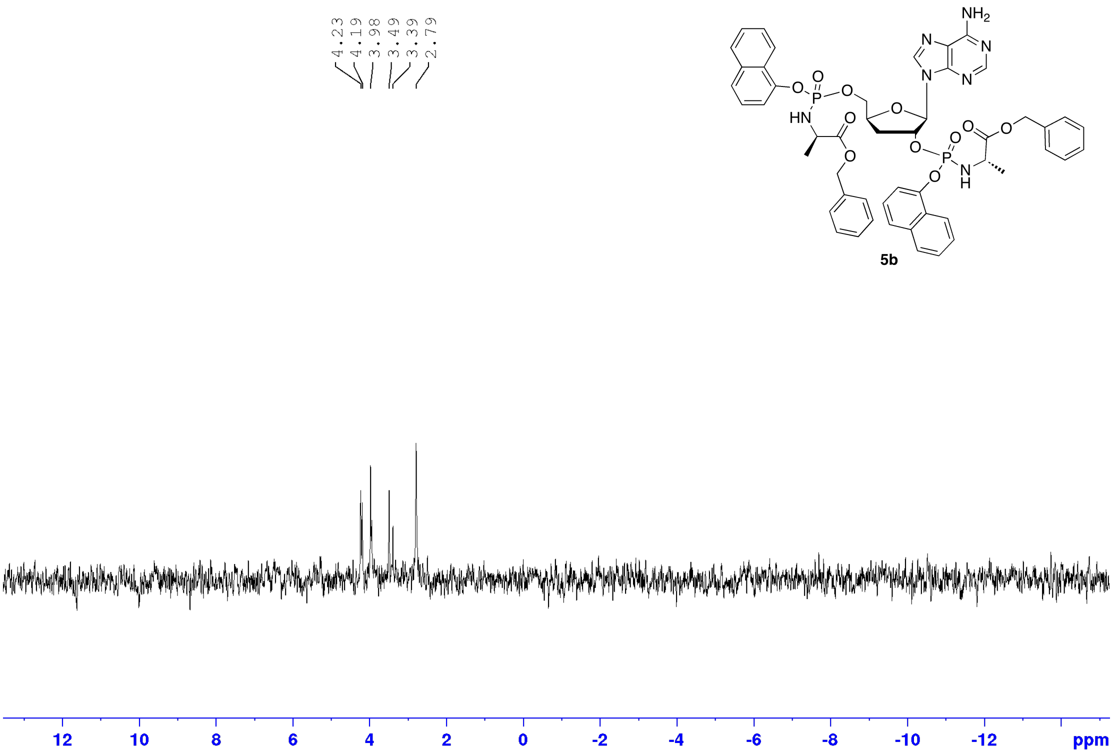

^{31}P-NMR (202MHz, CD_3OD)

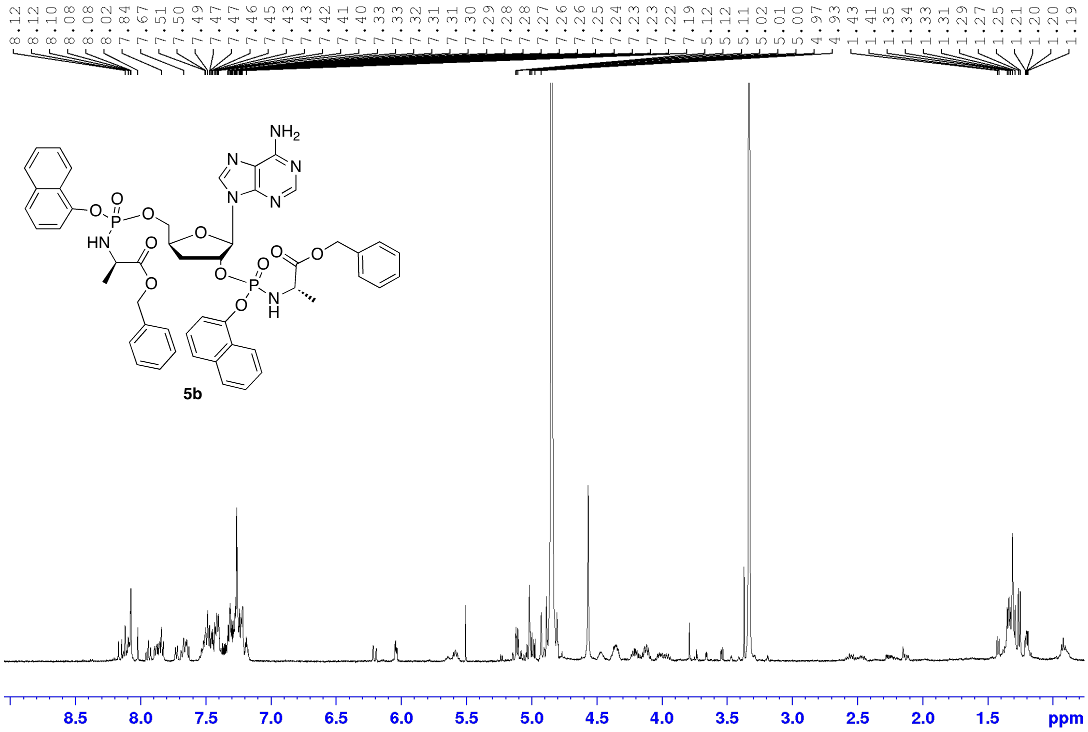

^{1}H-NMR (500MHz, CD_3OD)

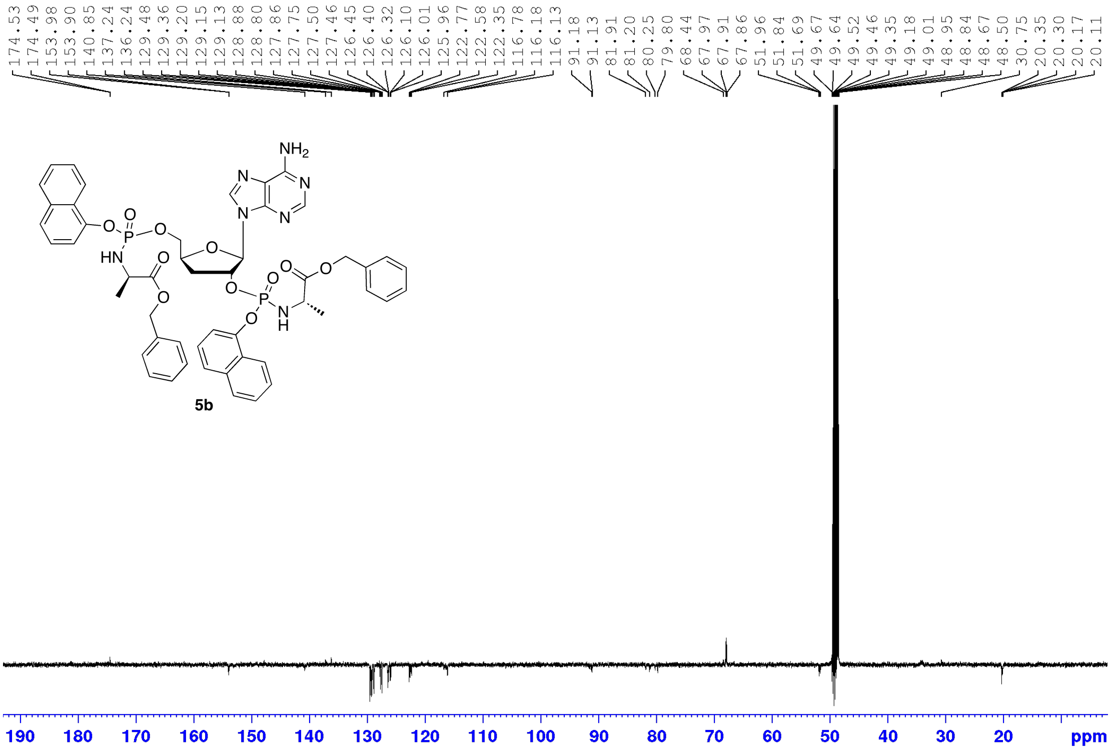

^{13}C-NMR (125MHz, CD_3OD)

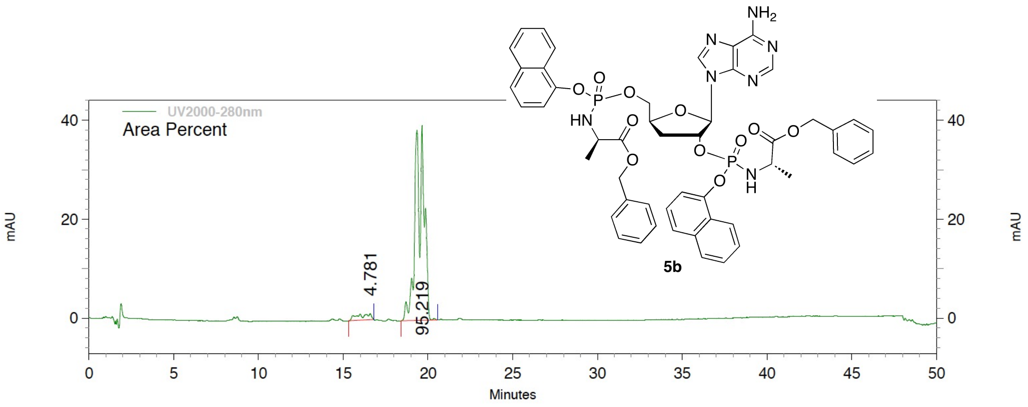

Retention Time	Area	Area %	Height	Height %
16.583	69760	4.78	1266	3.11
19.645	1389350	95.22	39404	96.89
Totals	1459110	100.00	40670	100.00

HPLC trace of **5b**

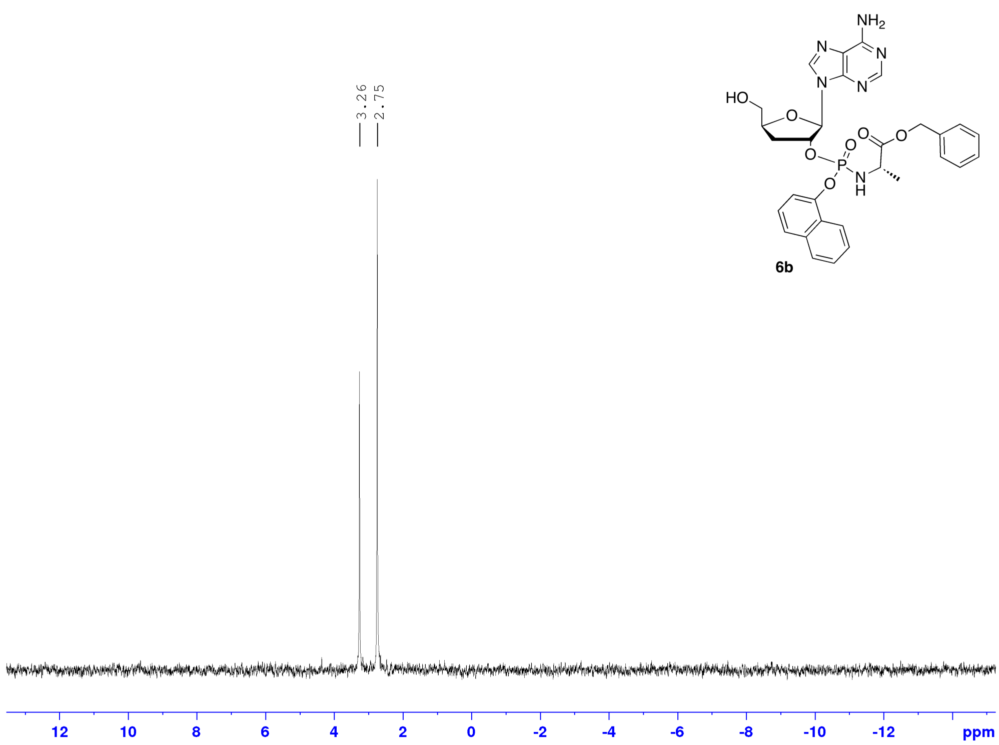

^{31}P-NMR (202MHz, CD_3OD)

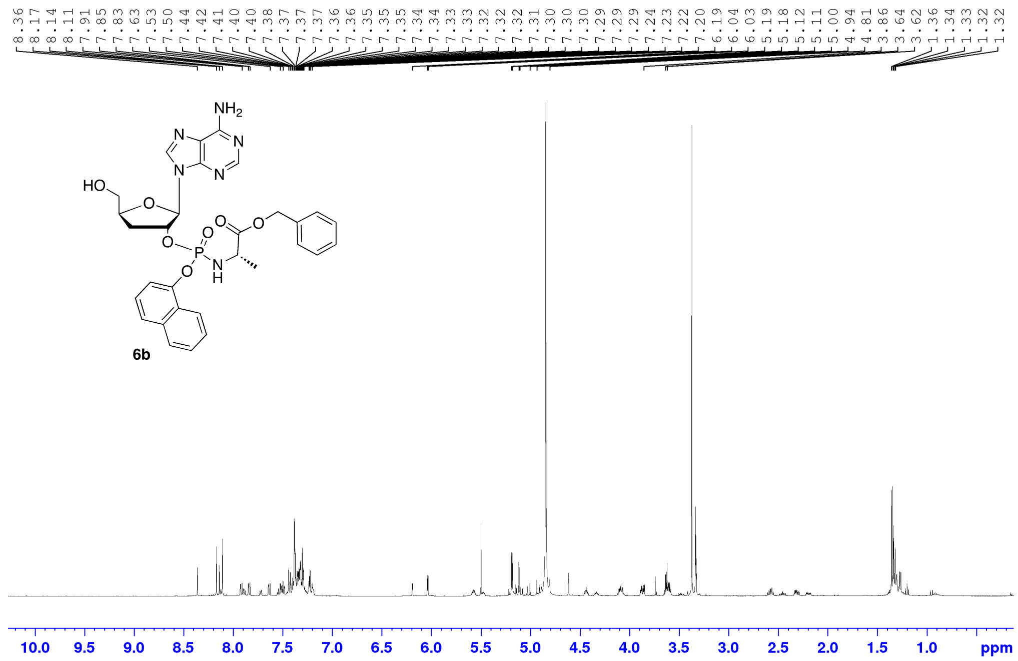

^{1}H-NMR (500MHz, CD_3OD)

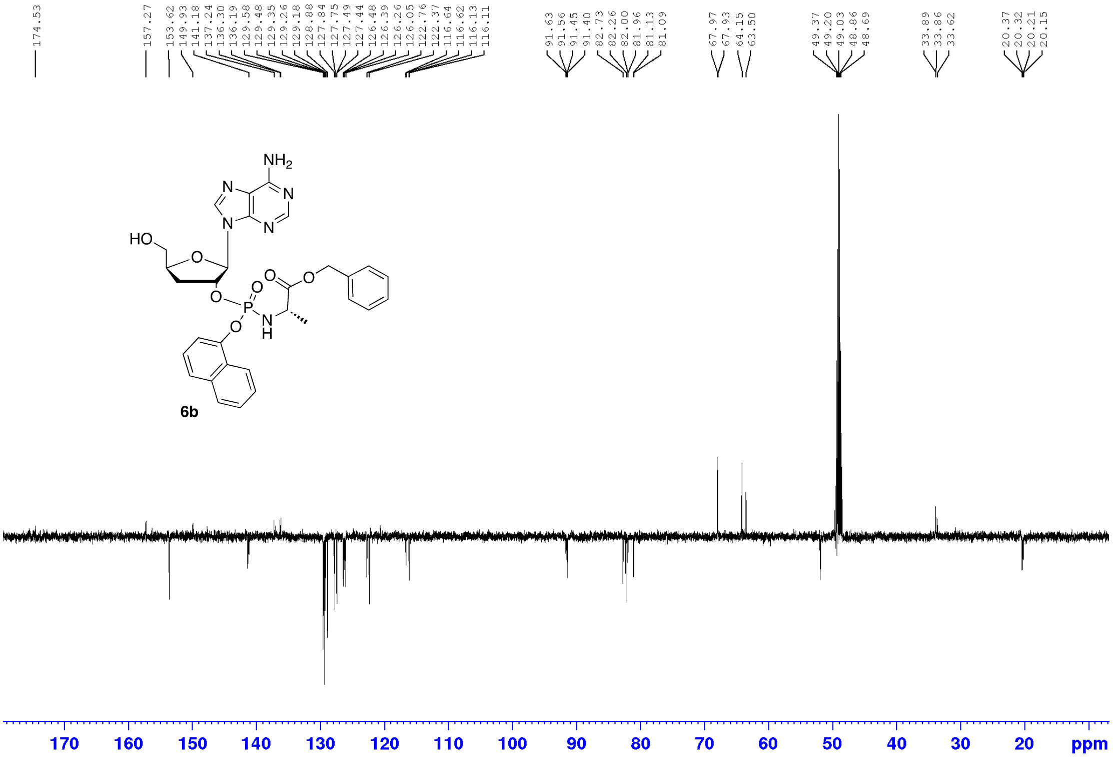

^{13}C-NMR (125MHz, CD_3OD)

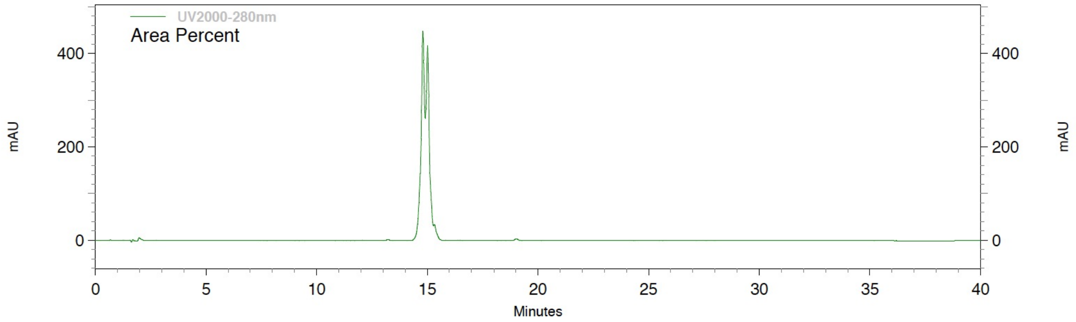

Retention Time	Area	Area %	Height	Height %
14.802	7699837	54.23	700699	52.08
15.012	6432929	45.31	632532	47.02
15.318	66060	0.47	12129	0.90
Totals	14198826	100.00	1345360	100.00

HPLC trace of **6b**

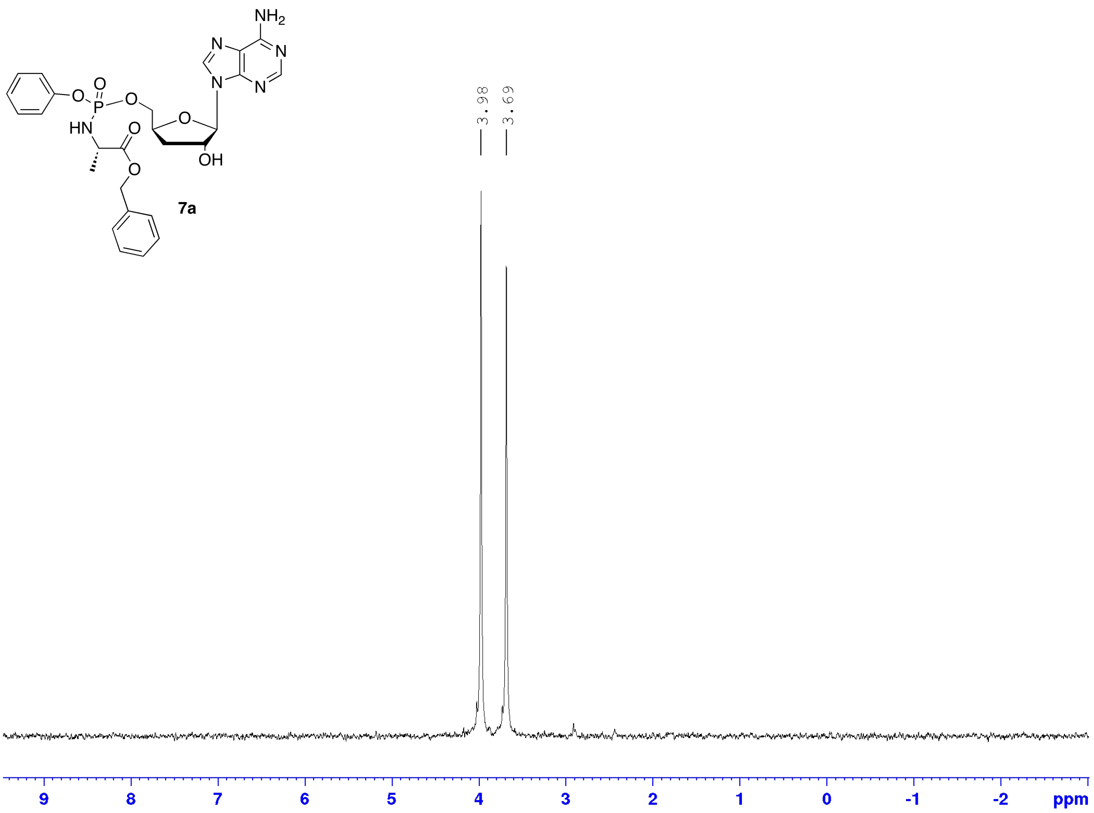

^{31}P-NMR (202MHz, CD_3OD)

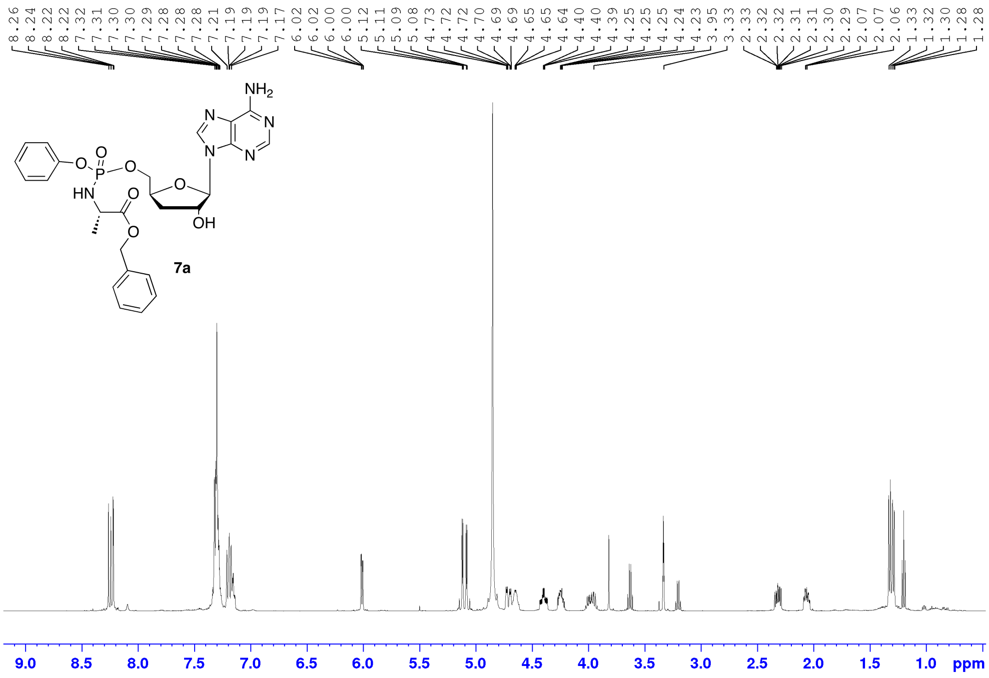

^{1}H-NMR (500MHz, CD_3OD)

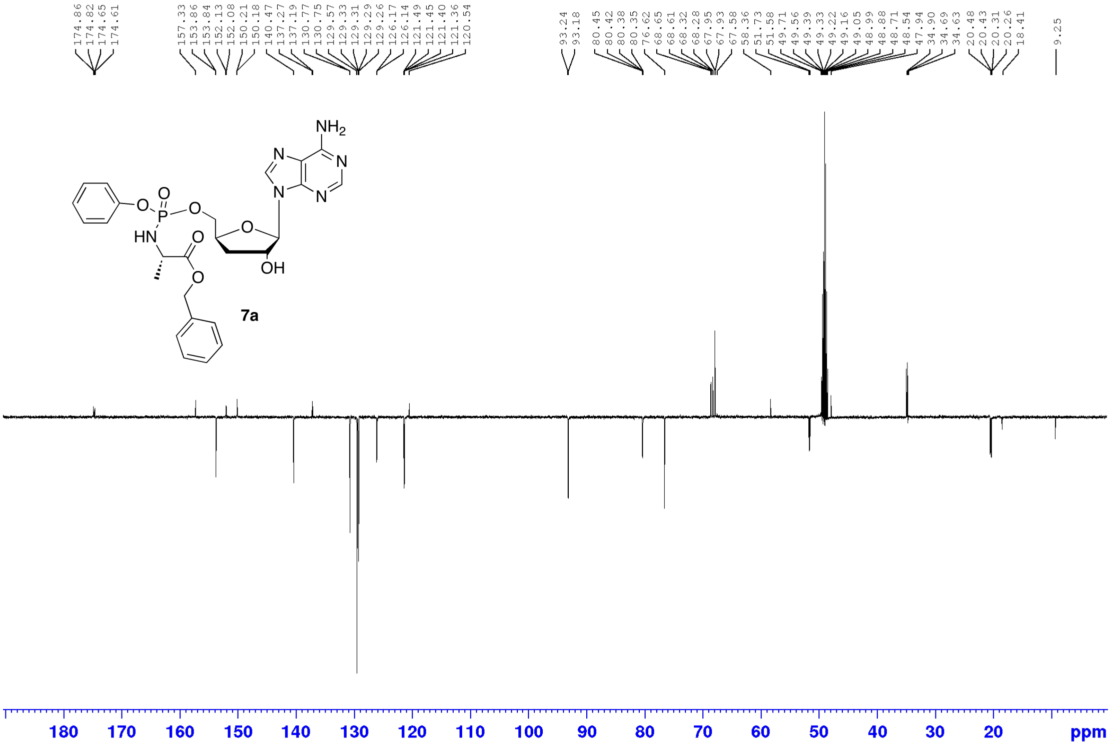

^{13}C-NMR (125MHz, CD_3OD)

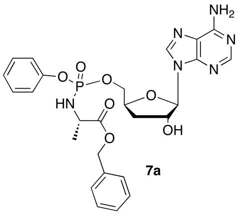

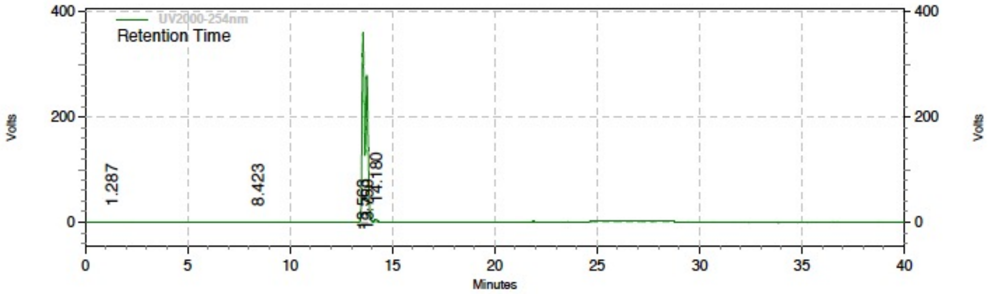

Retention Time	Area	Area %	Height	Height %
1.287	838	0.02	41	0.01
8.423	564	0.01	65	0.01
13.563	2729574	55.30	359289	56.05
13.750	2173608	44.03	277189	43.24
14.180	31514	0.64	4443	0.69
Totals	4936098	100.00	641027	100.00

HPLC trace of **7a**

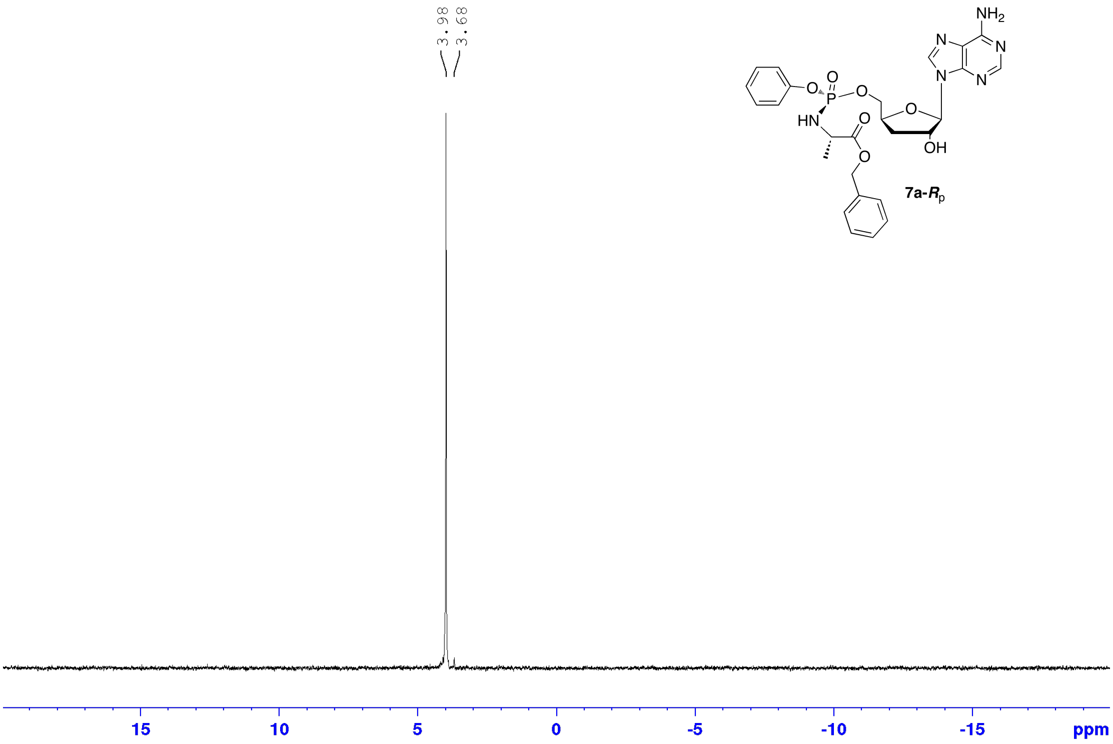

^{31}P-NMR (202MHz, CD_3OD)

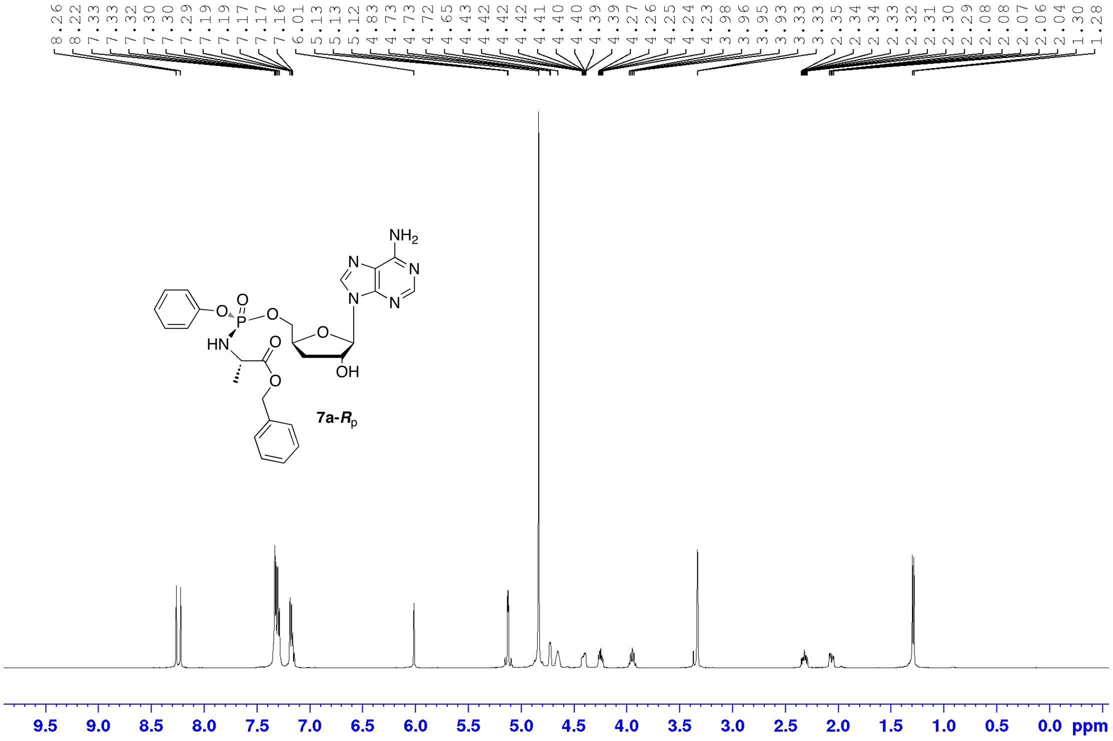

^{1}H-NMR (500MHz, CD_3OD)

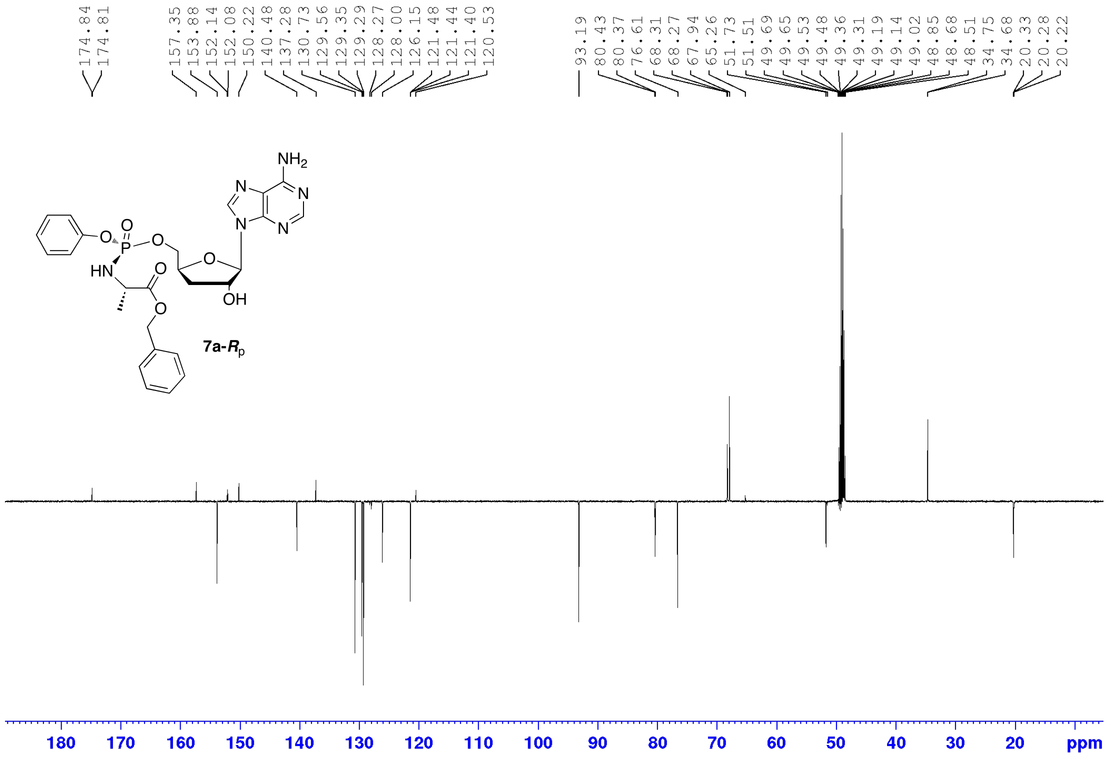

^{13}C-NMR (125MHz, CD_3OD)

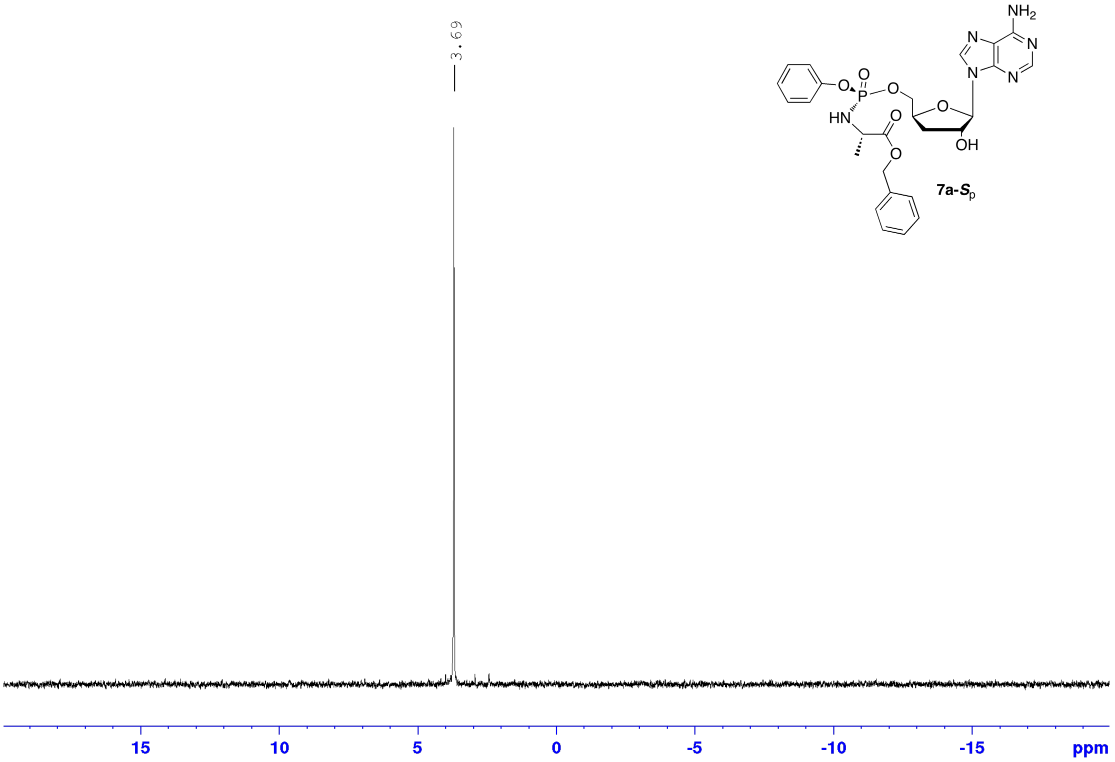

^{31}P-NMR (202MHz, CD_3OD)

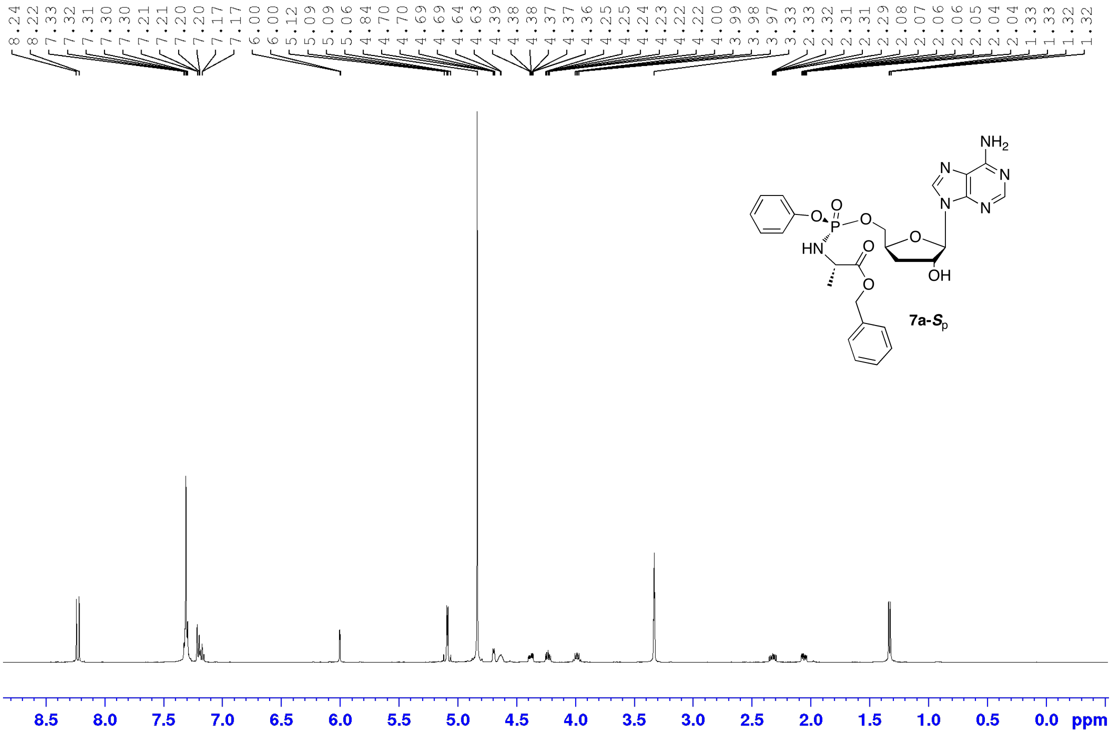

^{1}H-NMR (500MHz, CD_3OD)

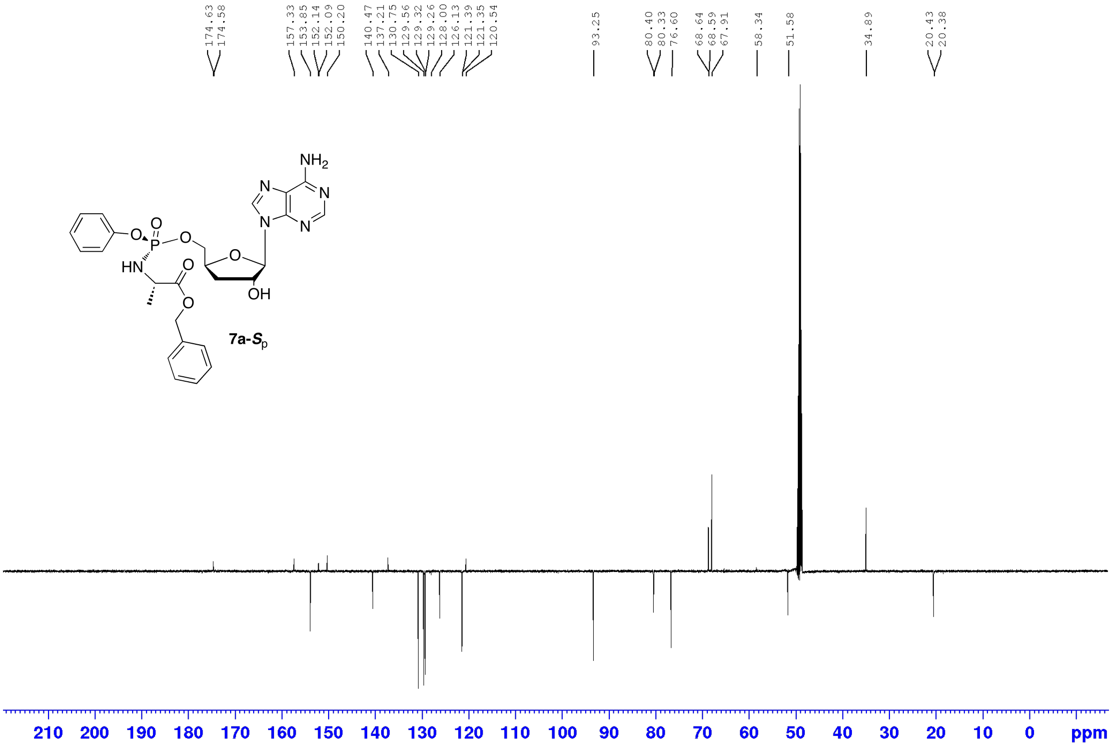

^{13}C-NMR (125MHz, CD_3OD)

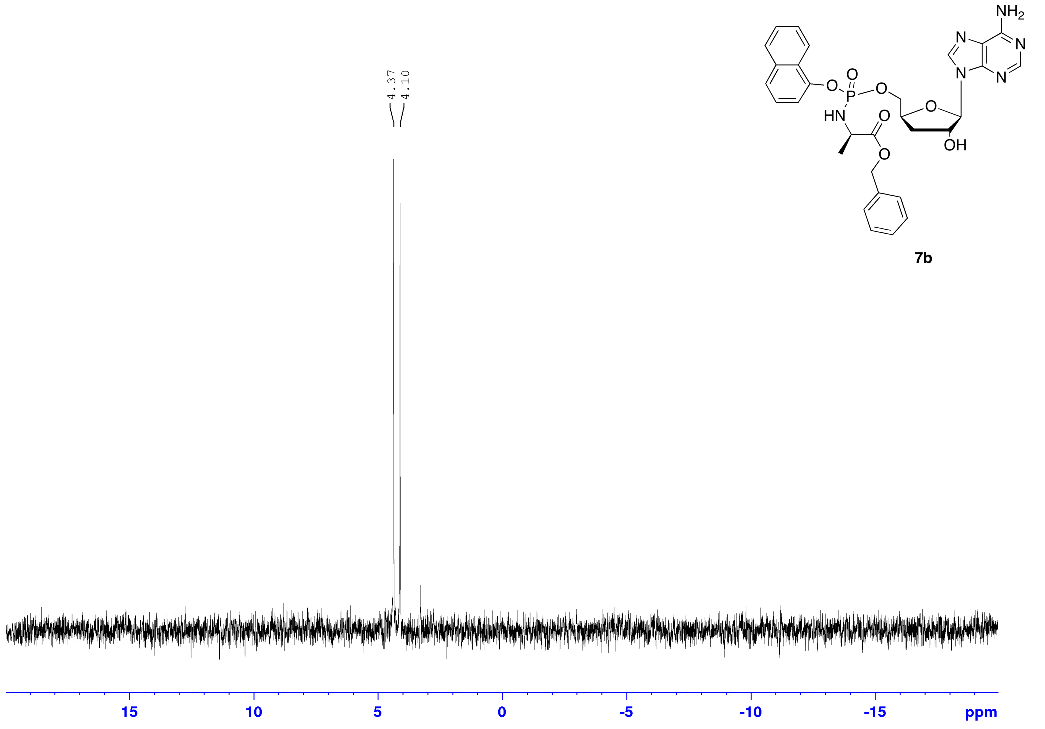

^{31}P-NMR (202MHz, CD_3OD)

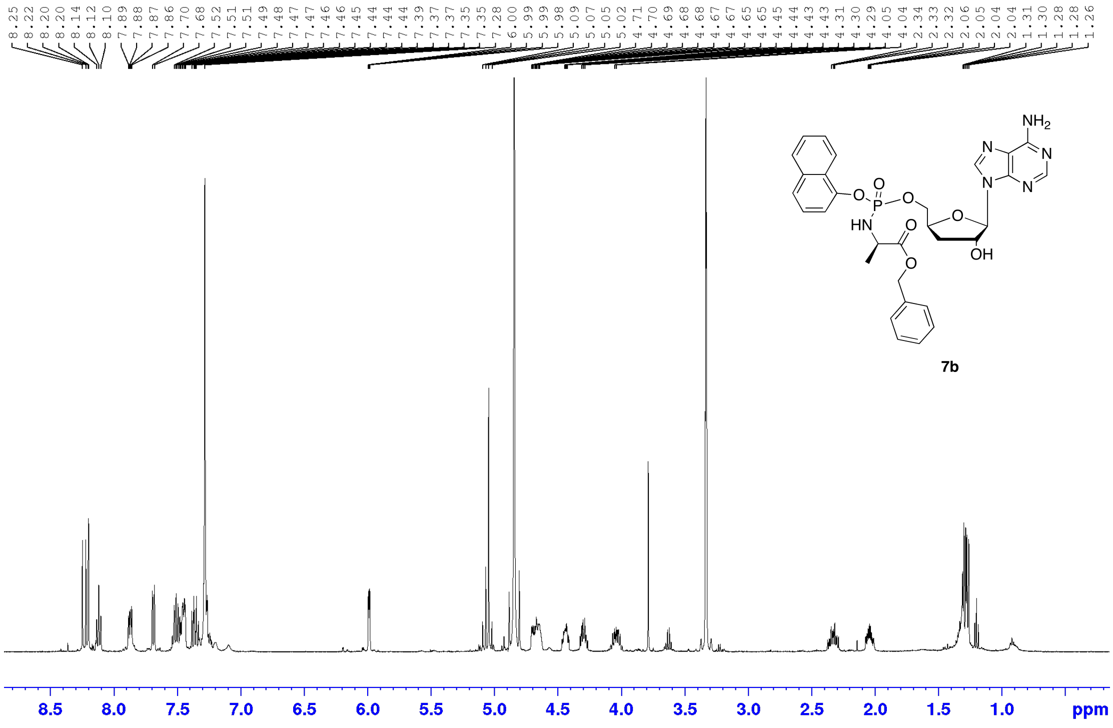

^{1}H-NMR (500MHz, CD_3OD)

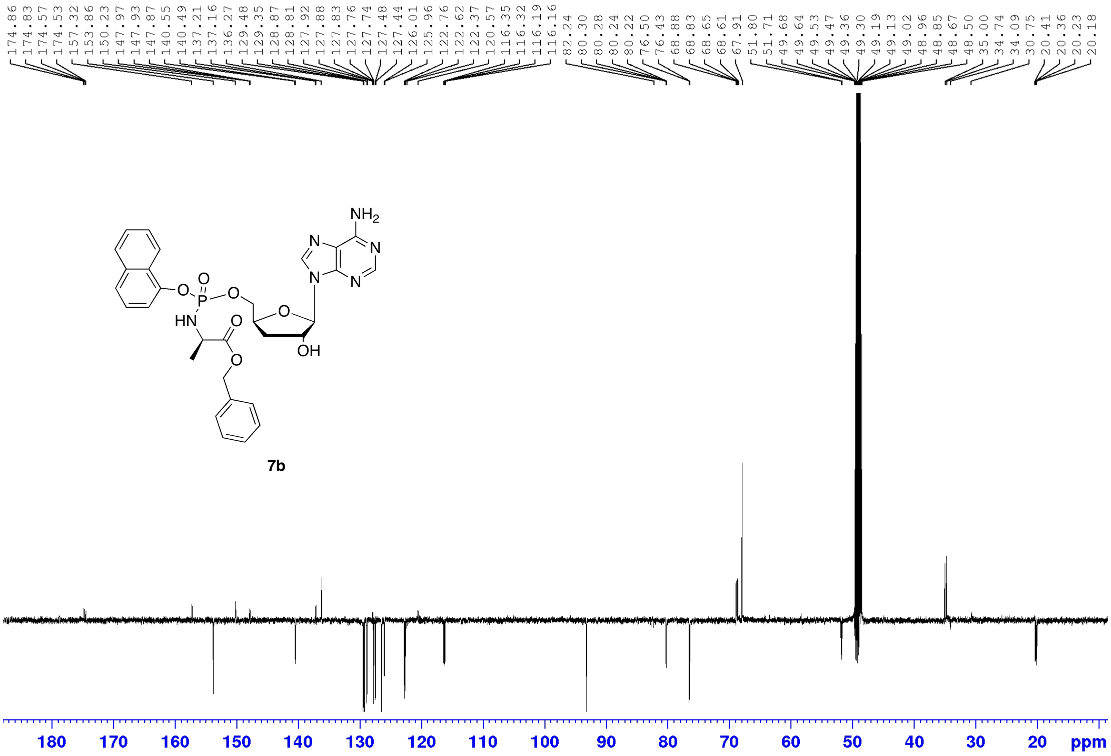

^{13}C-NMR (125MHz, CD_3OD)

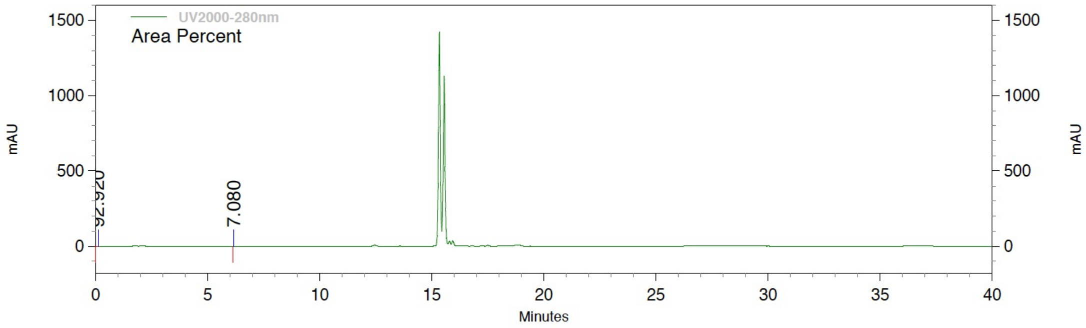

Retention Time	Area	Area %	Height	Height %
12.457	45355	0.18	10473	0.24
15.342	13617583	54.14	2392673	55.23
15.553	10777676	42.85	1866895	43.09
15.938	620990	2.47	55697	1.29
18.722	92459	0.37	6678	0.15
Totals	25154063	100.00	4332416	100.00

HPLC trace of **7b**

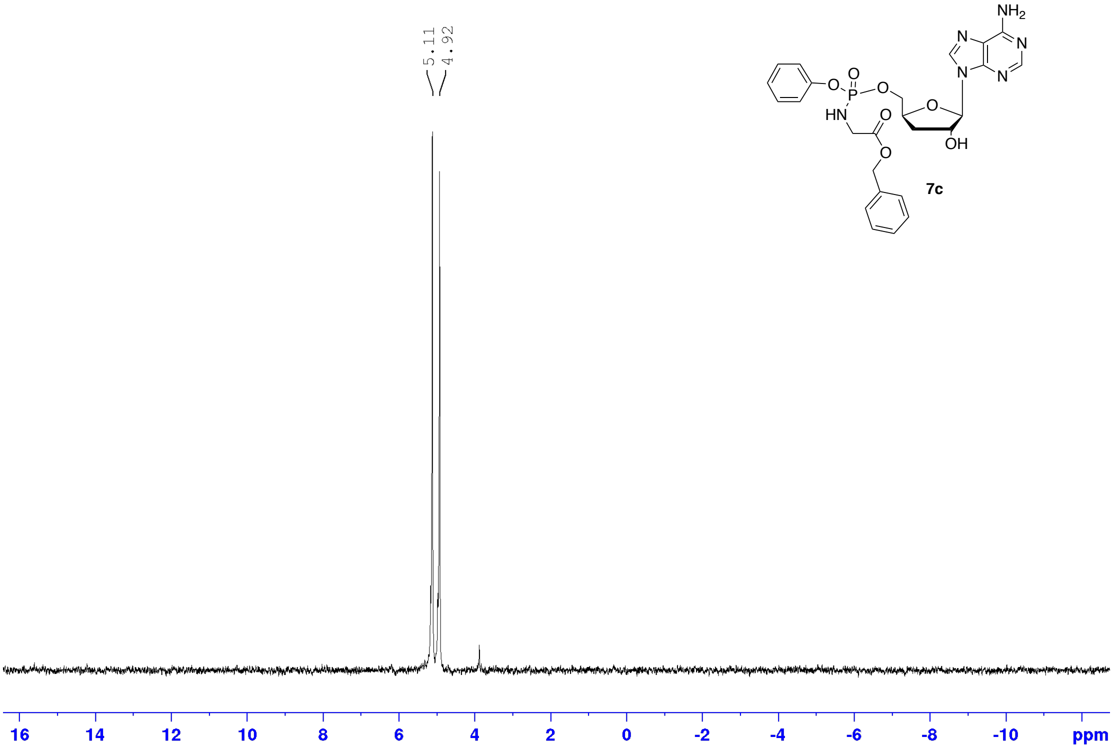

^{31}P-NMR (202MHz, CD_3OD)

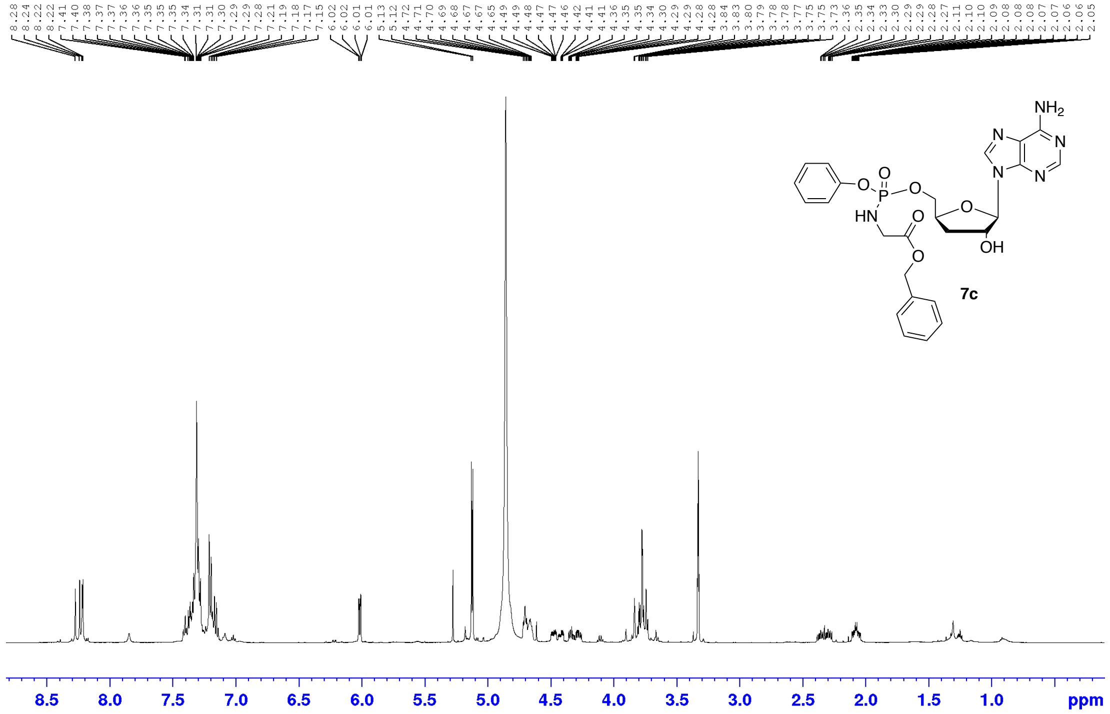

^{1}H-NMR (500MHz, CD_3OD)

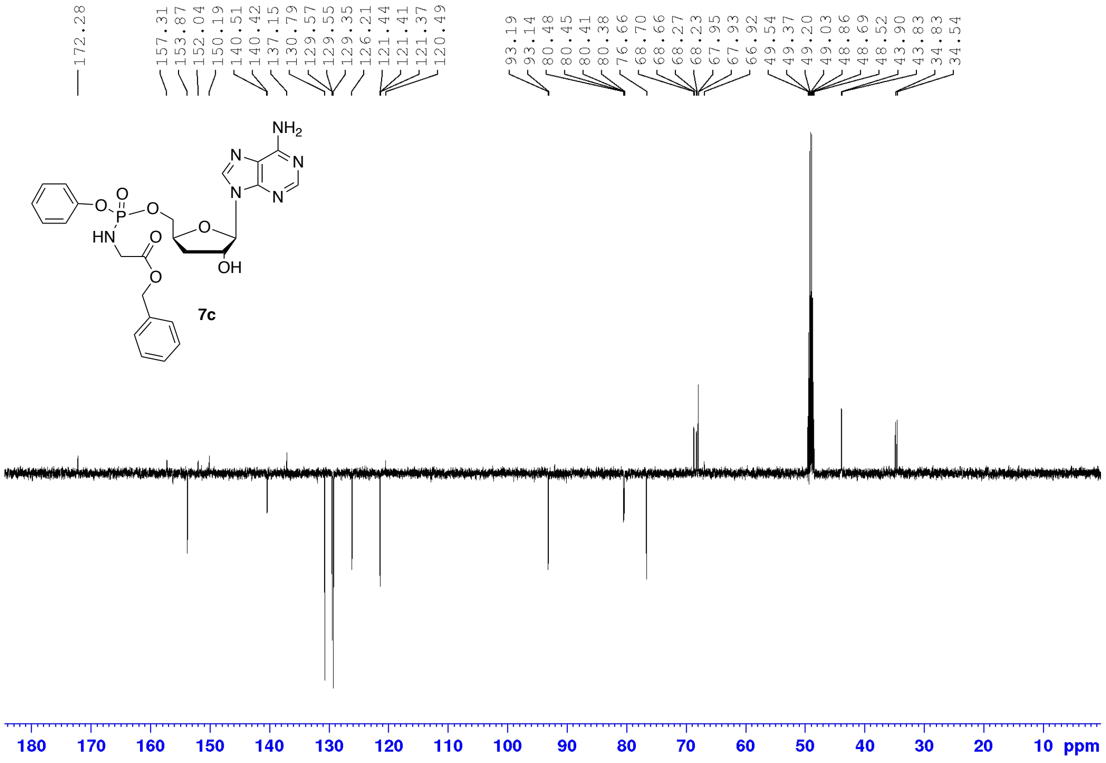

^{13}C-NMR (125MHz, CD_3OD)

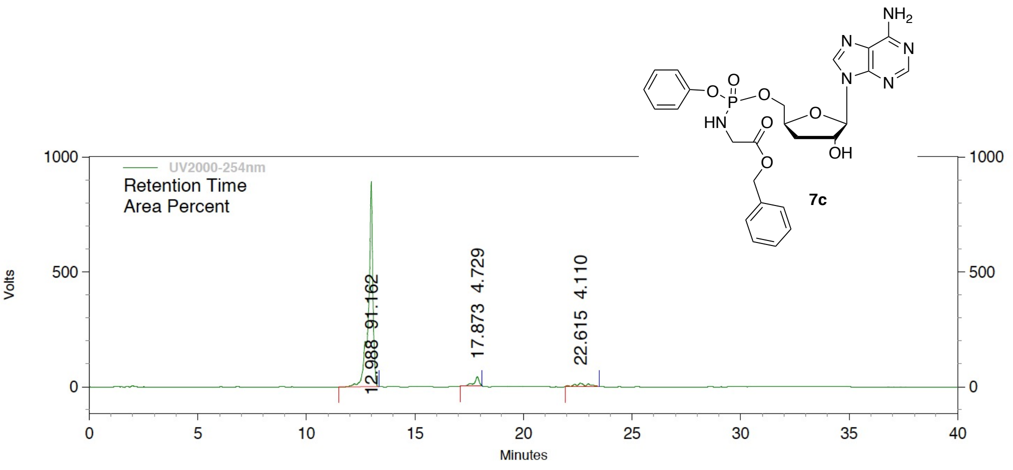

Retention Time	Area	Area %	Height	Height %
12.988	12567976	91.16	891967	94.27
17.873	651924	4.73	40345	4.26
22.615	566590	4.11	13853	1.46
Totals	13786490	100.00	946165	100.00

HPLC trace of **7c**

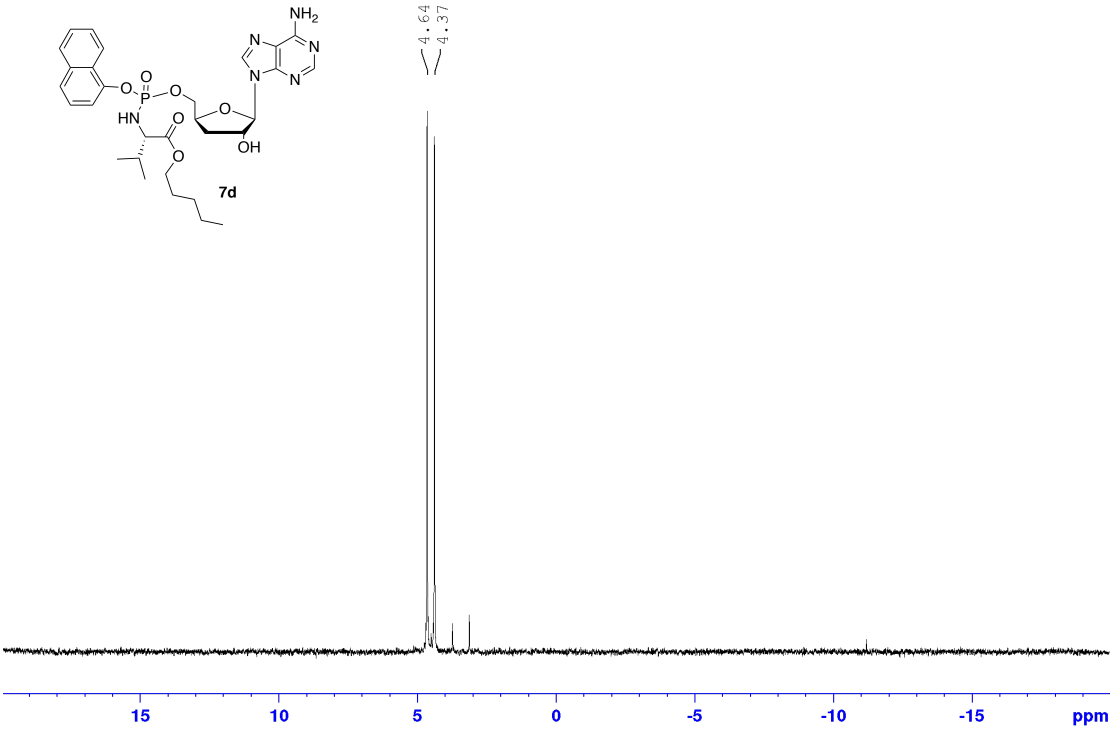

^{31}P-NMR (202MHz, CD_3OD)

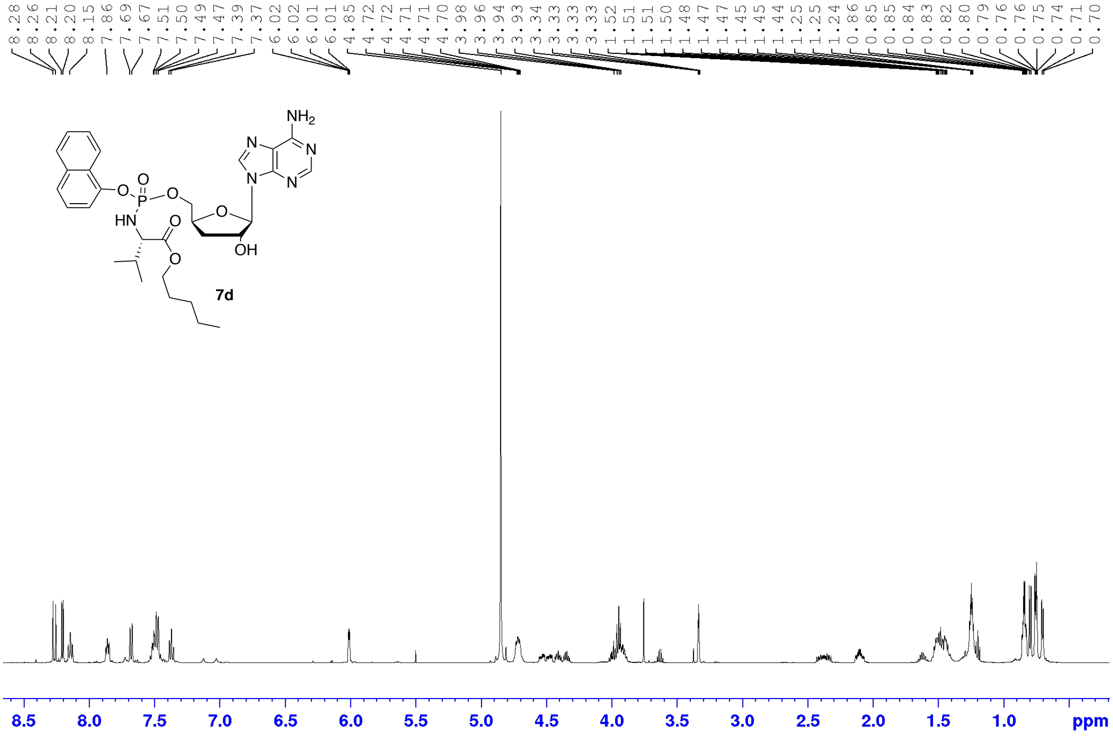

^{1}H-NMR (500MHz, CD_3OD)

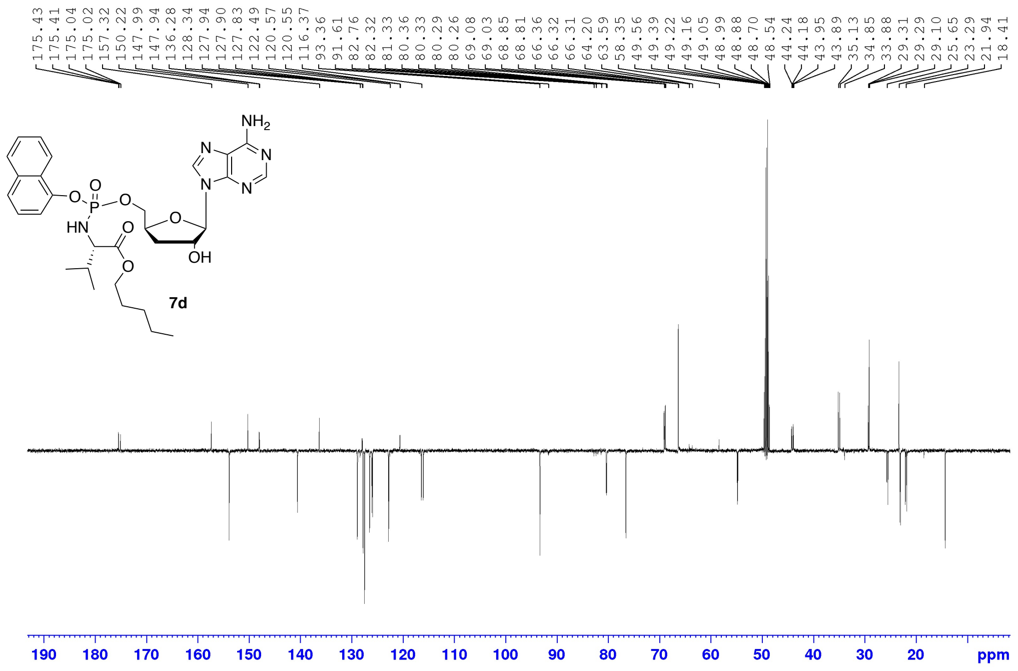

^{13}C-NMR (125MHz, CD_3OD)

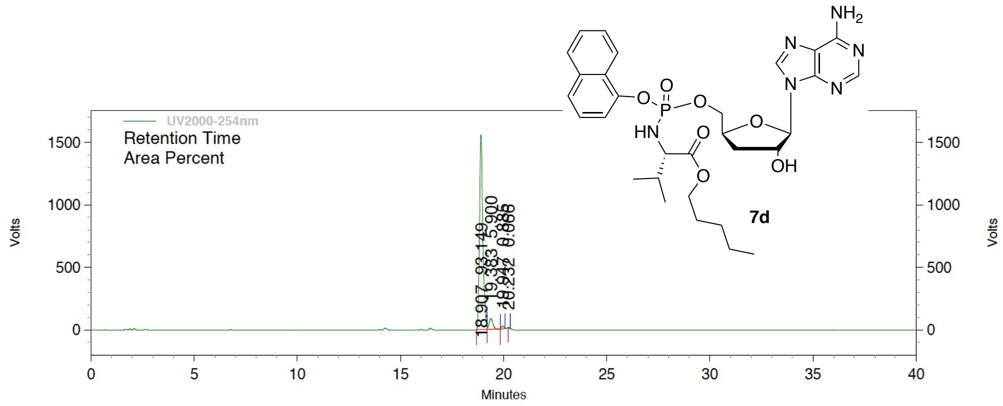

Retention Time	Area	Area %	Height	Height %
18.907	17512908	93.15	1557497	93.14
19.383	1109237	5.90	89391	5.35
19.947	166345	0.88	23018	1.38
20.232	12458	0.07	2354	0.14
Totals	18800948	100.00	1672260	100.00

HPLC trace of **7d**

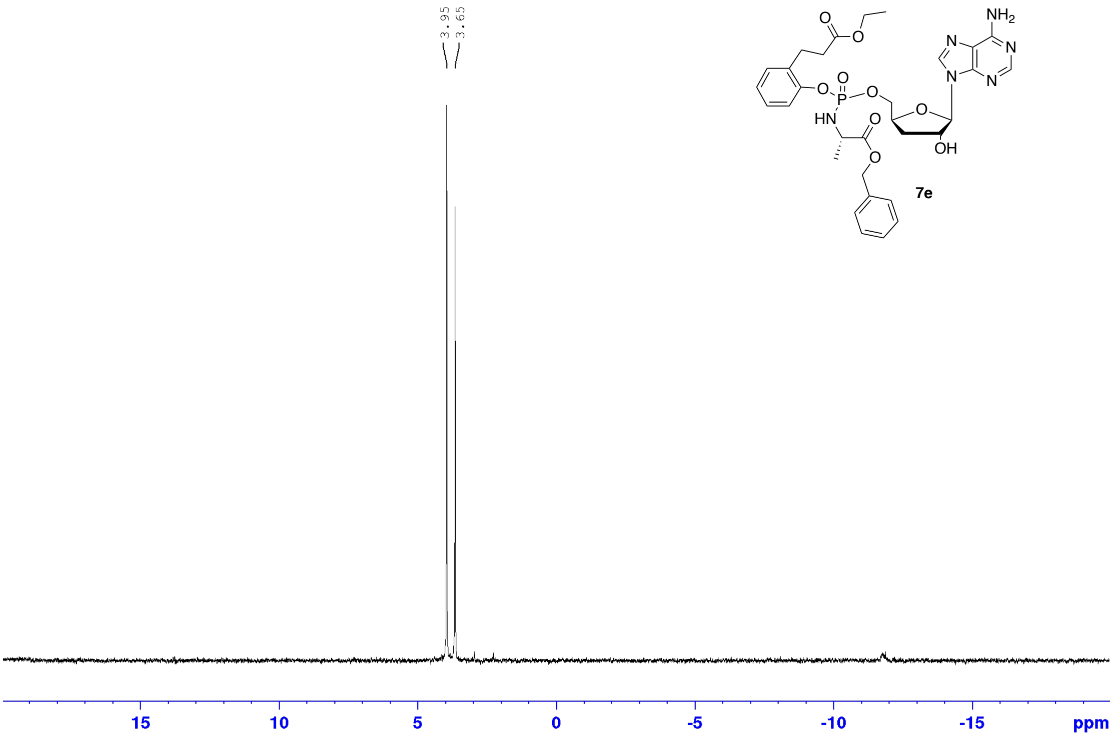

^{31}P-NMR (202MHz, CD$_3$OD)

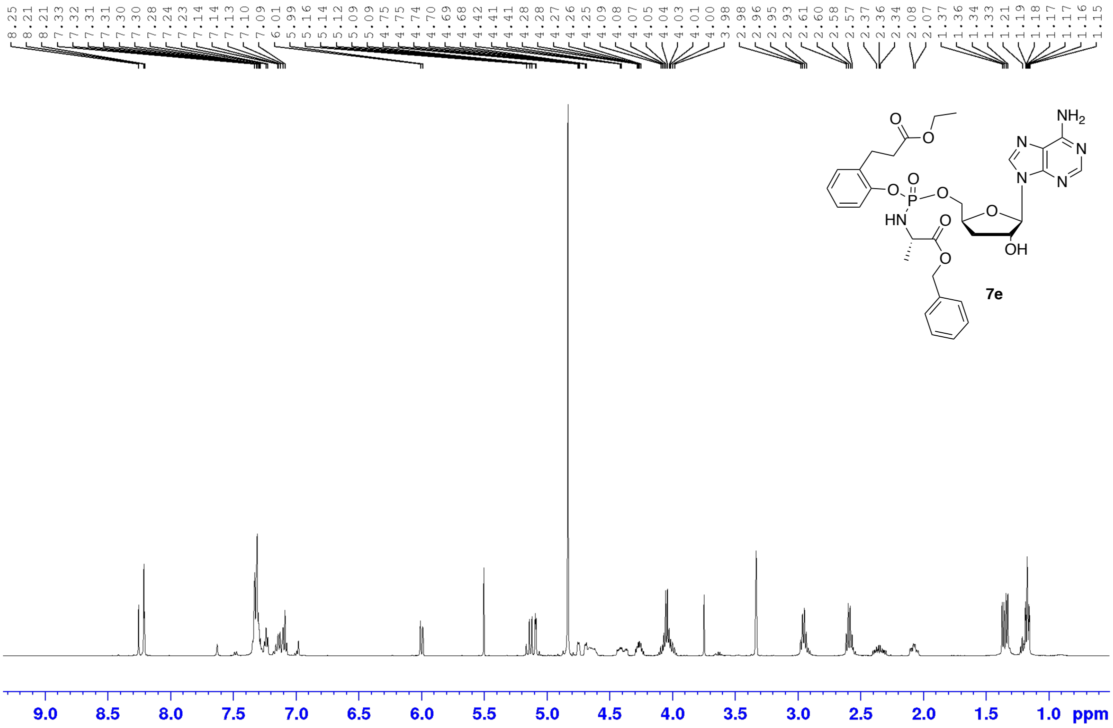

^{1}H-NMR (500MHz, CD$_3$OD)

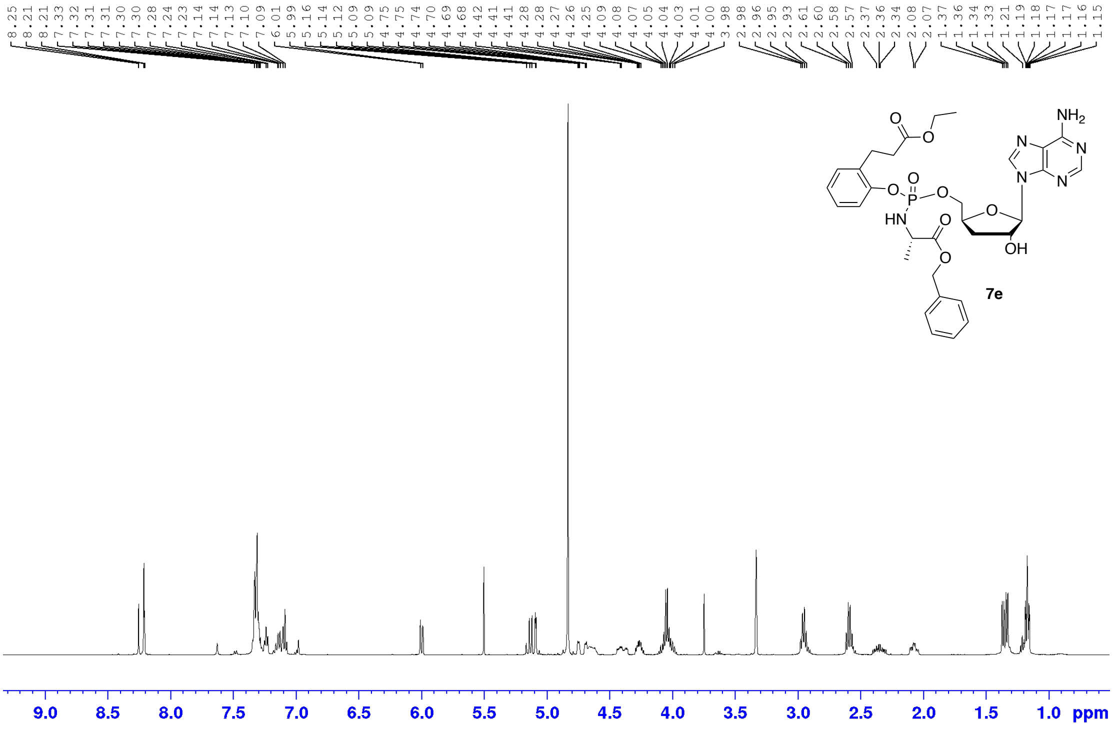

^{1}H-NMR (500MHz, CD_3OD)

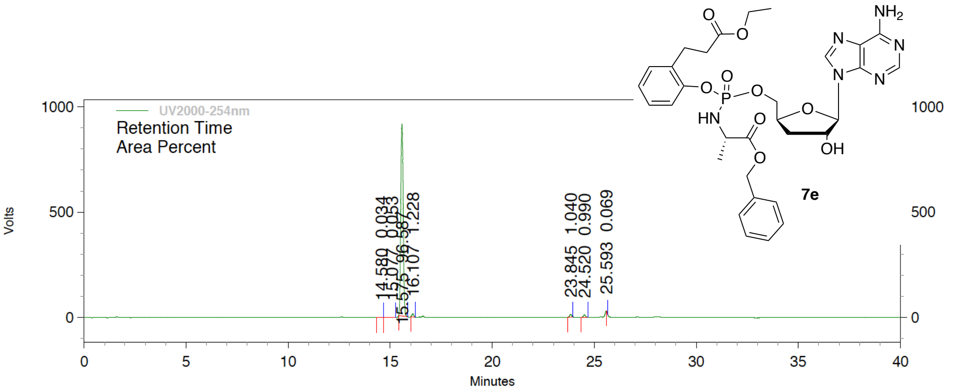

Retention Time	Area	Area %	Height	Height %
14.580	2675	0.03	372	0.04
15.077	4219	0.05	482	0.05
15.575	7642643	96.59	911469	95.66
16.107	97144	1.23	15127	1.59
23.845	82305	1.04	13421	1.41
24.520	78299	0.99	11948	1.25
25.593	5436	0.07	0	0.00
Totals	7912721	100.00	952819	100.00

HPLC trace of **7e**

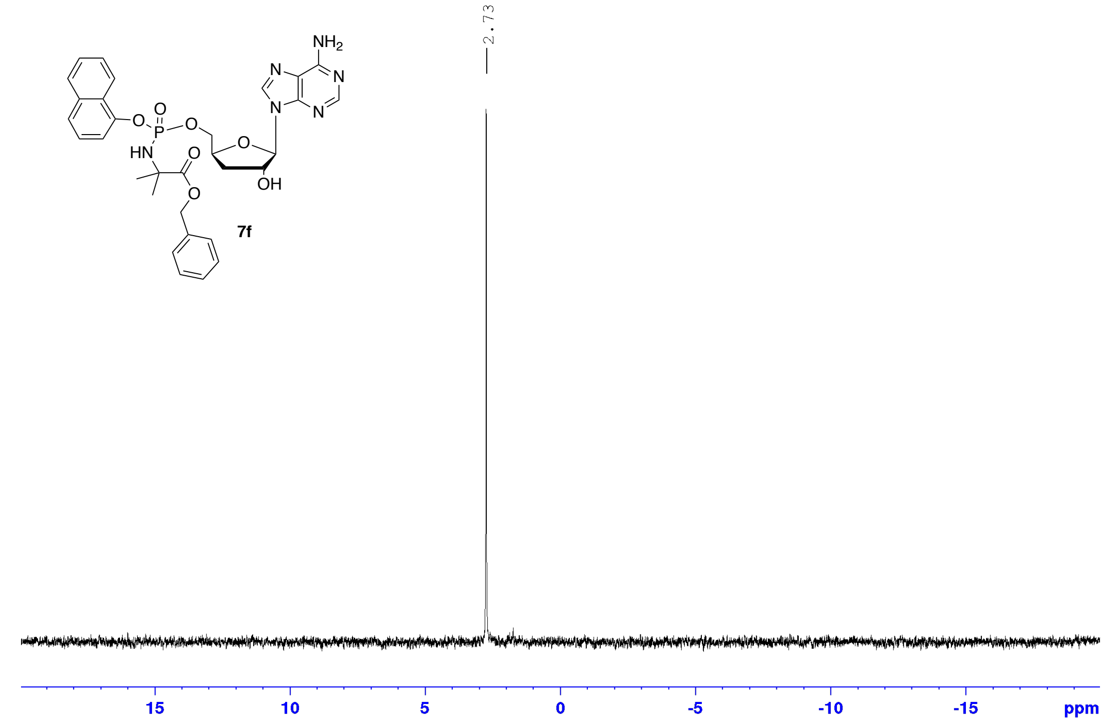

^{31}P-NMR (202MHz, CD_3OD)

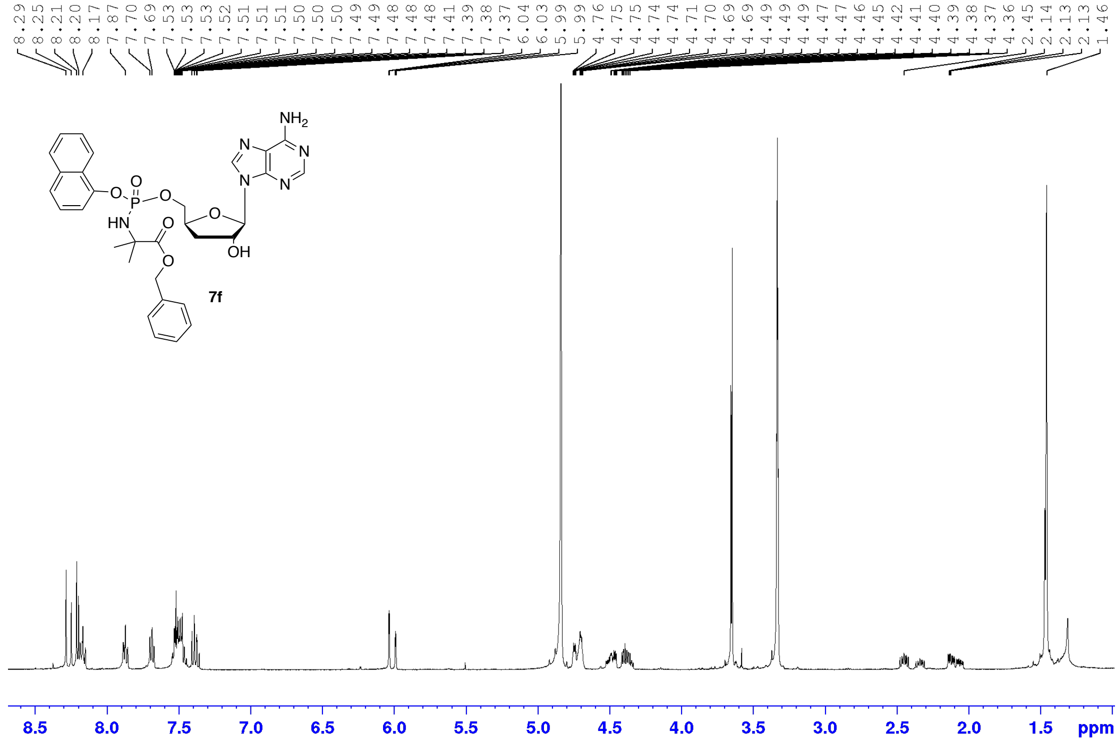

^{1}H-NMR (500MHz, CD_3OD)

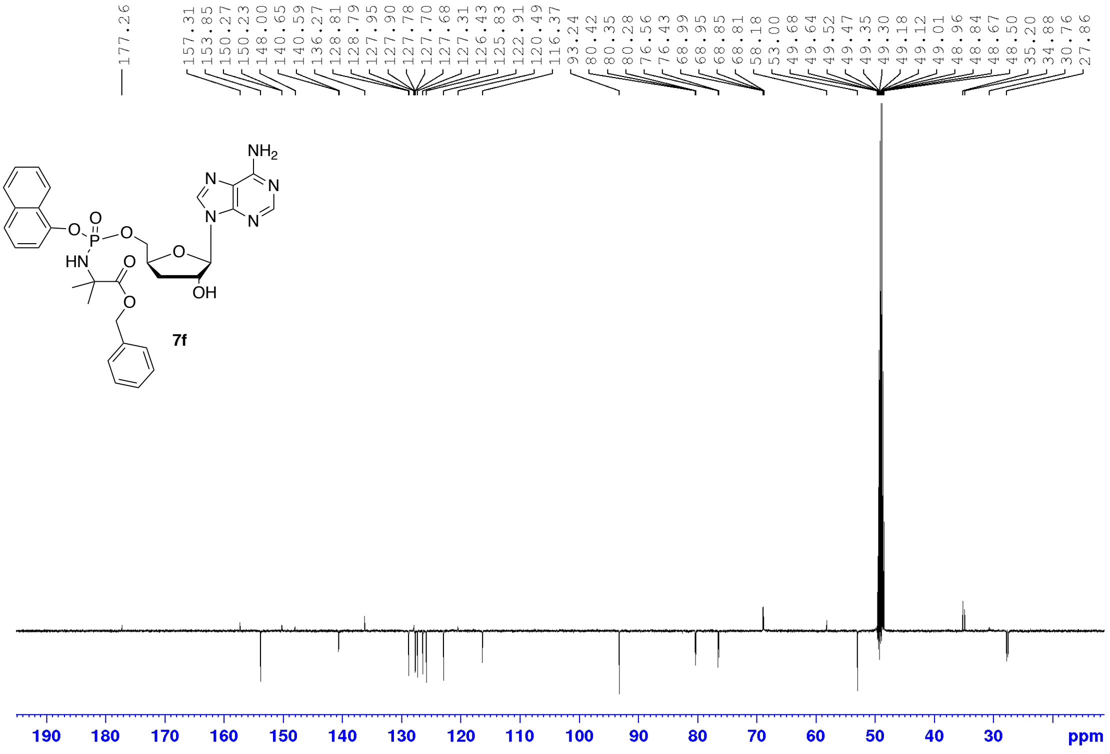

^{13}C-NMR (125MHz, CD_3OD)

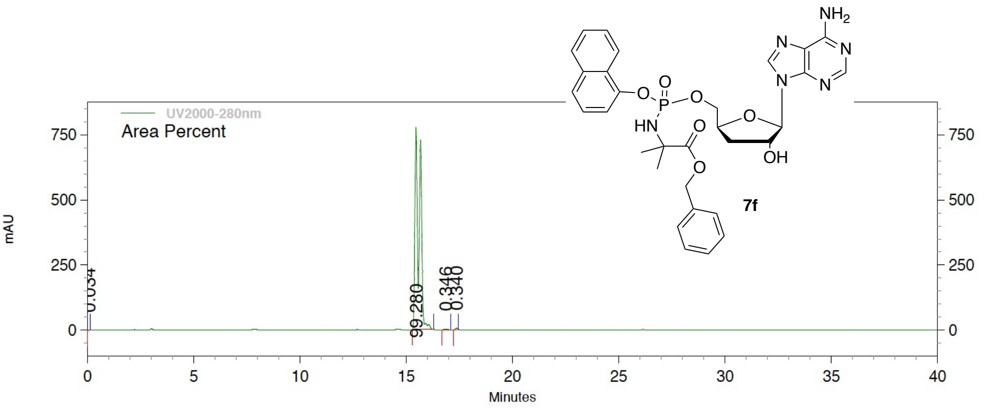

Retention Time	Area	Area %	Height	Height %
0.067	4043	0.03	780	0.10
15.458	11922506	99.28	776795	98.73
16.852	41506	0.35	3583	0.46
17.360	40880	0.34	5592	0.71
Totals	12008935	100.00	786750	100.00

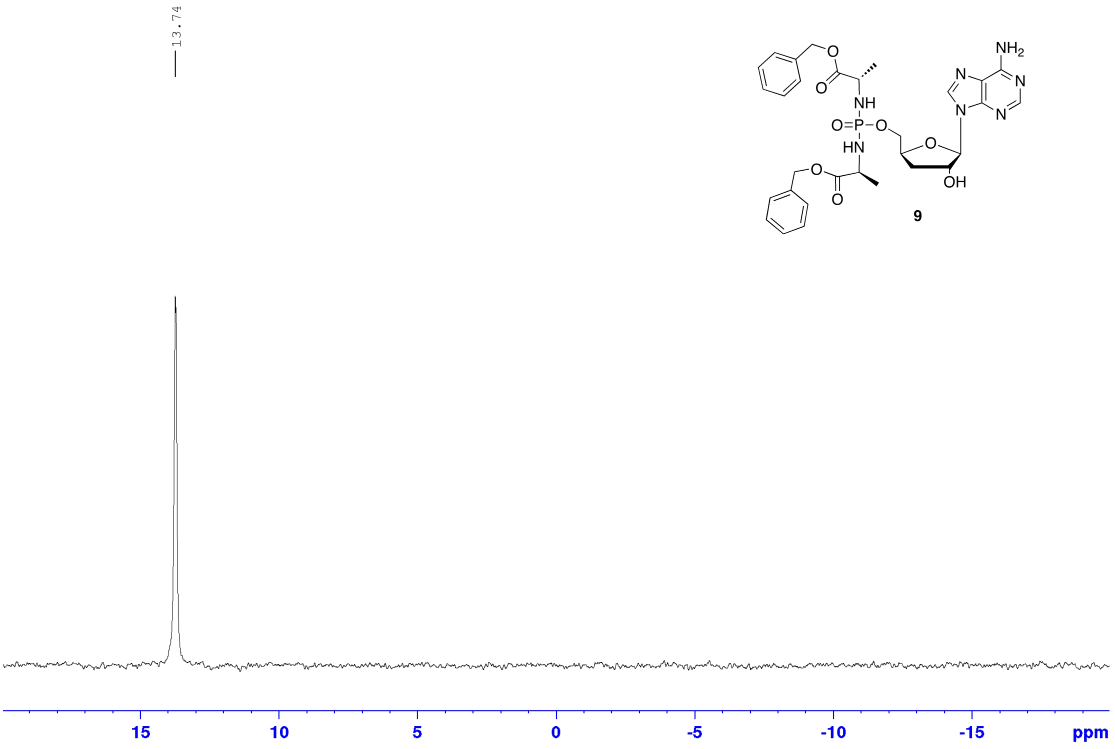

^{31}P-NMR (202MHz, CD_3OD)

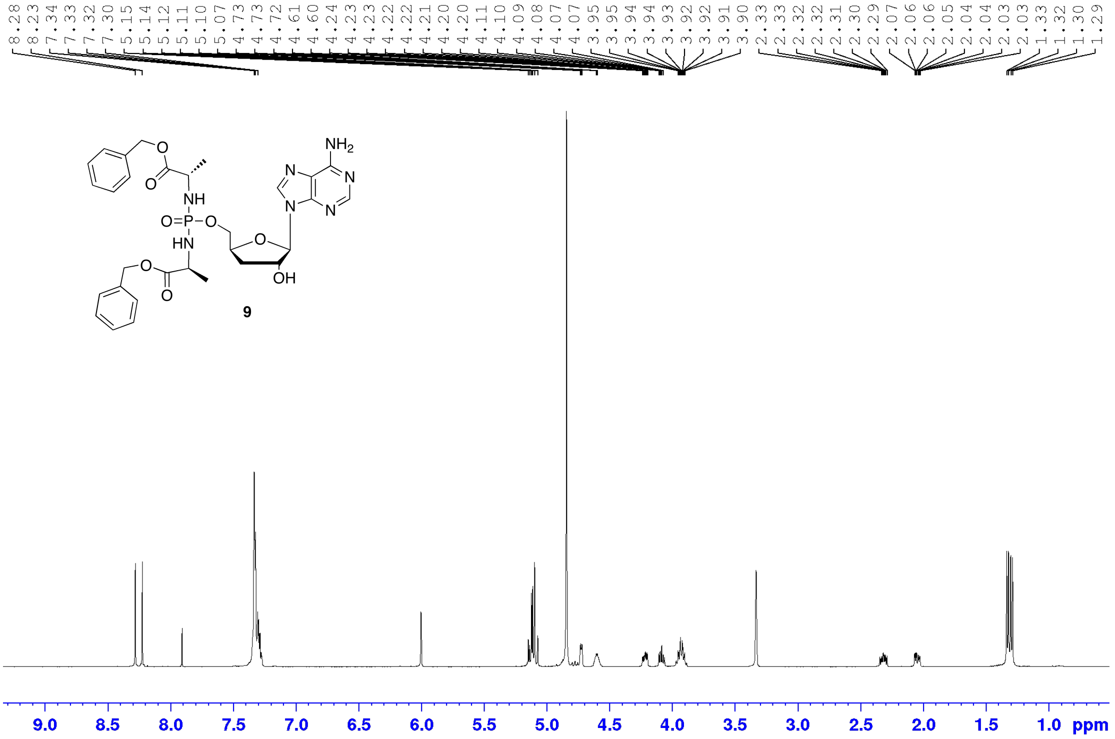

^{1}H-NMR (500MHz, CD_3OD)

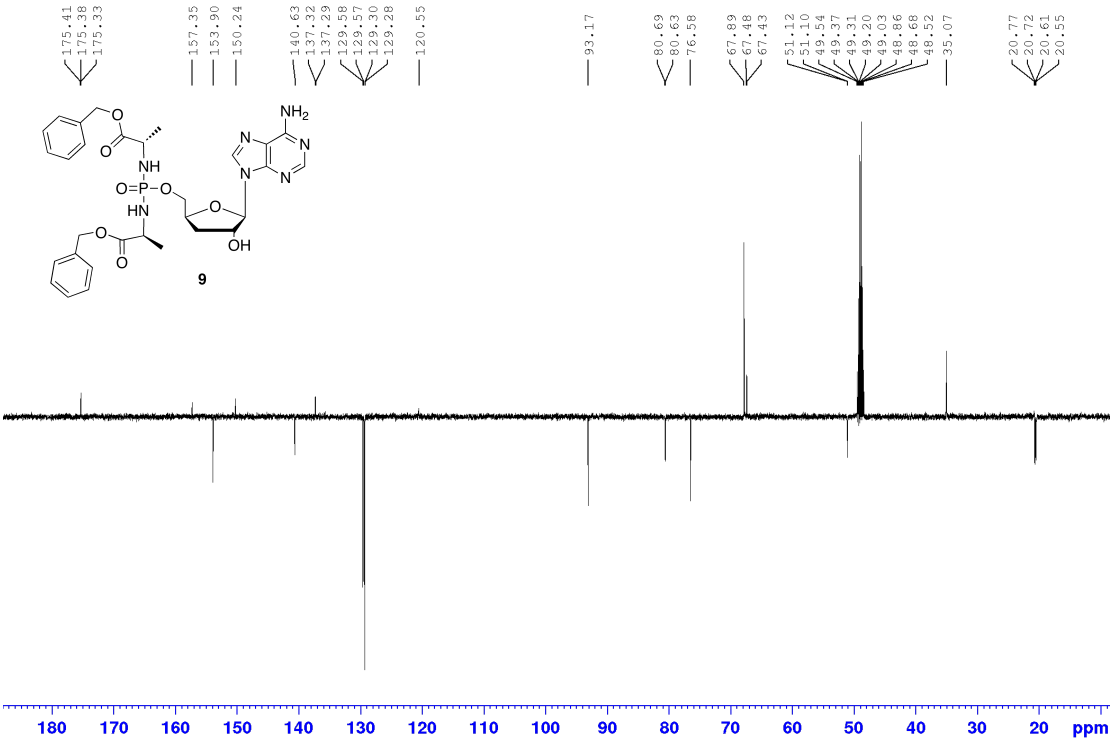

^{13}C-NMR (125MHz, CD_3OD)
